# Theoretical Characterization of New Frustrated Lewis Pairs for Responsive Materials

**DOI:** 10.3390/polym13101573

**Published:** 2021-05-14

**Authors:** Maialen Galdeano, Fernando Ruipérez, Jon M. Matxain

**Affiliations:** 1 POLYMAT, University of the Basque Country UPV/EHU, Joxe Mari Korta Center. Avda. Tolosa 72, 20018 Donostia, San Sebastián, Spain; maialen.galdeano@polymat.eu; 2 Polimero eta Material Aurreratuak: Fisika, Kimika eta Teknologia Saila, Kimika Fakultatea, Euskal Herriko Unibertsitatea UPV/EHU and Donostia International Physics Center (DIPC), P.K. 1072, 20080 Donostia, Euskadi, Spain

**Keywords:** self-healing polymers, dynamic bonds, reversible chemistry, frustrated Lewis pairs, triphenylborane derivatives, triphenylphosphine derivatives

## Abstract

In recent years, responsive materials including dynamic bonds have been widely acclaimed due to their expectation to pilot advanced materials. Within these materials, synthetic polymers have shown to be good candidates. Recently, the so-called frustrated Lewis pairs (FLP) have been used to create responsive materials. Concretely, the activation of diethyl azodicarboxylate (DEAD) by a triphenylborane (TPB) and triphenylphosphine (TPP) based FLP has been recently exploited for the production of dynamic cross-links. In this work, we computationally explore the underlying dynamic chemistry in these materials, in order to understand the nature and reversibility of the interaction between the FLP and DEAD. With this goal in mind, we first characterize the acidity and basicity of several TPB and TPP derivatives using different substituents, such as electron-donating and electron-withdrawing groups. Our results show that strong electron-donating groups increase the acidity of TPB and decrease the basicity of TPP. However, the FLP–DEAD interaction is not mainly dominated by the influence of these substituents in the acidity or basicity of the TPB or TPP systems, but by attractive or repulsive forces between substituents such as hydrogen bonds or steric effects. Based on these results, a new material is proposed based on FLP–DEAD complexes.

## 1. Introduction

Dynamic chemistry is gaining significance in polymer science and engineering, since a large variety of responsive materials have been developed with unique properties attributed to the nature of the dynamic bond, such as reprocessing, recycling or self-healing capacity [1]. These dynamic bonds must experience a fast reversible cleavage, so that an effective and well-planned structural design is decisive to adjust the dissociation and activation energies. This tailoring includes the use of both steric [2,3,4] and electronic effects [5,6]. The reversible response observed in these materials may be triggered by different stimuli, such as chemical, biological or physical, resulting in a change of one or more properties in the material. Further research in this field may lead to the development of dynamically controlled systems with diverse applications ranging from drug delivery and robotics to (bio)sensors and self-healing materials [7]. In this context, synthetic polymers have been recognized as good candidates to include dynamic features, since they can be chemically altered easily resulting in a responsive mechanism that can be readily manipulated.

Within responsive materials, self-healing polymers have been intensively explored because of their broad applications and the range of healing mechanisms available [8,9,10,11]. A self-healing material is defined as a material that has the ability to repair itself (either partially or totally) autonomously or in response to an external stimulus such as mechanical damage, heat or light [12,13]. Based on the principles of reversible chemistry, these materials are designed in such a way that the reorganization of the chemical bonds leads to a reconnection of the damaged parts, ultimately resulting in either a partial or complete recovery of the material. Many different chemistries have been explored to introduce healing functionality in polymeric materials, and two main approaches, depending on the nature of the reversible bond, can be devised: (i) materials based on dynamic covalent bonds [1,14,15,16,17,18,19,20,21], using the retro-Diels–Alder reaction [22,23,24], dichalcogenide bonds [25,26,27,28,29,30,31,32], siloxane chemistry [33] transesterification, [34], transcarbamoylation [35], transamidation [36] or alkoxyamine chemitry [37], for instance, and (ii) based on non-covalent interactions, such as π–π stacking [38,39,40], hydrogen bonds [41,42,43,44,45], metal–ion interactions [46,47,48,49,50,51] or ionomers [11,52].

In order to achieve a reversible self-healing material that is autonomous or needs weak stimulation, an appropriate choice of the dynamic bond is a key feature. In this sense, the design of new bonds that may serve for self-healing purposes is fundamental. Despite the large number of reversible chemistries available, it is still a challenge to develop self-healing materials based on dynamic bonds without changing the overall performance of the material [53]. For instance, certain dynamic bonds can only be applied to a particular polymer and, therefore, it would be desirable to identify new bonds that may expand the field and be easily integrated into a larger number of polymeric systems. Following this approach, Shaver and coworkers have explored a new type of self-healing material based on the reversible interaction between a frustrated Lewis pair (FLP) and a specific small molecule (diethyl azodicarboxylate, DEAD) [54]. For the first time, an experimental research team has taken the FLP concept into a new area by producing dynamic cross-linked networks that provide polymer gels with self-healing properties.

Stephan and coworkers introduced the idea of a frustrated Lewis pair in 2006 [55]. This is a recent paradigm for chemical reactivity based on impeded dative bonding between a Lewis acid and a Lewis base. In the last decade, it has been shown that the introduction of steric hindrance or a dissociative equilibrium results in free electron donors and acceptors that no longer have the capacity to form a dative bond, creating a FLP [56,57,58] and promoting activation of small molecules such as H${}_{2}$, CO${}_{2}$, NO or CO. Thus, Shaver and coworkers have designed a polymer containing FLP cross-links, that is, including electron donor and acceptor molecules that may interact reversibly with a small molecule to generate a responsive network. In particular, the monomers 4-styryl-diphenylborane and 4-styryl-dimesitylphosphine are incorporated in a polystyrene main chain. The diethyl azodicarboxylate (DEAD) reacts with boron and phosphorous atoms forming a gel. As the FLP interactions are dynamic, high temperatures (100 °C) may cleave the dative bonds [54,59].

Apart from this pioneering work, the introduction of FLPs into molecular materials has been, to our knowledge, scarcely considered [60,61]. Weak interaction energies have been calculated in the activation of small molecules by FLPs. For example, the binding energy of CO${}_{2}$ with the phosphine–borane pair is only of around 18 kcal/mol [62], which is in the range of a medium-strength hydrogen bond [63], and can be comparable to those present in a supramolecular responsive material. Thus, based on these relevant results, we believe it is very important to theoretically characterize the interactions present in the material proposed by Shaver and provide with new information in order to develop a brand new kind of responsive materials activated by small molecules, exploiting the dynamic nature of the FLP bonding.

**Figure 1:**
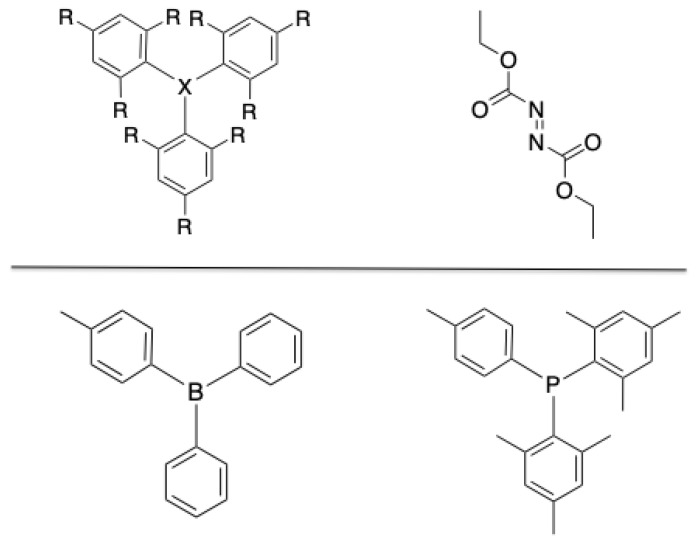
Top: Molecular models of the Lewis acids (X = B) and bases (X = P) substituted by electron-donating (R = CH${}_{3}$, NH${}_{2}$, OH, OCH${}_{3}$ and OCOCH${}_{3}$) and electron-withdrawing (R = F, CF${}_{3}$, CN, NO${}_{2}$ and SO${}_{3}$H) functional groups (left), and the linker molecule, DEAD (right). Bottom: Reference Lewis acid and base from the experimental work [54].

In this work, for the first time in the literature, a computational study of the interaction between a set of frustrated Lewis pairs with the small molecule used by Shaver (DEAD, see Figure 1, top-right panel) is performed. Concretely, Lewis acids and bases based on triphenylborane (TPB) and triphenylphosphine (TPP) derivatives, including both electron-donating (EDG) and electron-withdrawing (EWG) substituents in ortho and para positions of the phenyl rings (see Figure 1, top-left panel) have been considered. Hence, the main goal of this work is to achieve a better comprehension of the interactions between the mentioned species and unveil the relevant parameters to design and suggest new improved candidates for self-healing materials.

## 2. Materials and Methods

All geometry optimizations and vibrational frequency calculations were carried out within density functional theory (DFT) [64,65] using the Gaussian 16 program package [66]. Concretely, geometries were optimized in gas phase using the TPSS exchange-correlation functional [67], combined with the def2-TZVP basis set [68,69]. Dispersion interactions were considered using the empirical D3 version of Grimme’s dispersion with Becke–Johnson damping [70]. This level of theory was proposed by Schrimer and Grimme as the most appropriate for weak acid–base interactions, such as those existing in frustrated Lewis pairs [71]. After geometry optimizations, harmonic vibrational frequencies were obtained by analytical differentiation of gradients, at the same level of theory, to identify if the characterized structures were true minima. Such frequencies were then used to evaluate the zero-point vibrational energy (ZPVE) and the thermal (T = 298 K) vibrational corrections to the enthalpy. The interaction energies include the correction of the basis set superposition error (BSSE) by means of the counterpoise method [72,73].

Finally, the nature of the interaction was analyzed using the natural bonding orbital (NBO) [74,75,76] and the energy decomposition analysis (EDA) [77,78] methodologies. The EDA method, based on the energy partition of Morokuma [79], focuses on the instantaneous interaction energy, $\Delta E_{int}$, between two fragments (A and B) in a bond A-B, in the particular electronic reference state and in the frozen geometry of AB. This interaction energy is divided into three main components and the additional dispersion term, $\Delta E_{disp}$:

(1) $$\Delta E_{int}=\Delta E_{elstat}+\Delta E_{Pauli}+\Delta E_{orb}+\Delta E_{disp}$$

In order to obtain the contributions of the interaction energy, three steps corresponding to the bond formation process are followed. In the first step, the A and B fragments are brought from infinite separation to the position in the molecule. These fragments have frozen charge densities and the interaction between these charge densities at the equilibrium geometry of AB corresponds to the quasi-classical electrostatic interaction, $\Delta E_{elstat}$, which is usually attractive. The product wavefunction ($\psi_{A}\psi_{B}$) is normalized but violates the Pauli principle. Thus, in the second step, this product wavefunction is antisymmetrized and renormalized to provide an intermediate state, $\phi^{0}$ and the corresponding energy $E^{0}$. The energy difference between $E^{AB}$ and $E^{0}$ corresponds to the exchange Pauli repulsion term, $\Delta E_{Pauli}$. This terms involves the destabilizing interactions between electrons of the same spin on either fragment. The last step is the relaxation of the intermediate state, $\psi^{0}$, to define a final state, $\phi_{AB}$. The energy lowering of this step is related to the mixing of orbitals (charge transfer and polarization effects) and is regarded as the covalent contribution to the chemical bond ($\Delta E_{orb}$).

The EDA calculations were performed using the BP86 functional [80,81] with a triple-ζ quality basis set (ADF basis set TZP), using the program package ADF2017 [82].

## 3. Results and Discussion

First of all, in Section 3.1, the acidity and basicity of the TPB and TPP derivatives mentioned in the introduction are evaluated using different parameters. Recall that different EDG and EWG are considered in order to evaluate their influence. Afterwards, in Section 3.2, the interaction energies between these species and the DEAD linker molecule used experimentally by Shaver and coworkers [54] are computed and analyzed. Notice that the Cartesian coordinates of all optimized structures are given in the Supplementary Information.

### 3.1. Acidity of TPB Derivatives and Basicity of TPP Derivatives

**Figure 2:**
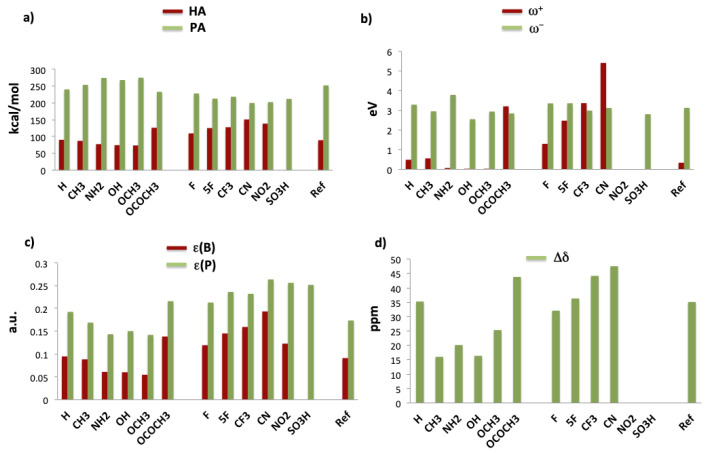
(**a**) Hydride affinity (HA, red bars, absolute values) and proton affinity (PA, green bars), in kcal/mol; (**b**) electroaccepting power ($\omega^{+}$·10, red bars) and electrodonating power ($\omega^{-}$, green bars), in eV; (**c**) boron empty orbital energy (ε(B), red bars) and phosphorous lone-pair orbital energy (ε(P), green bars), in a.u. (absolute values); (**d**) variation of the ${}^{31}$P NMR chemical shift (Δδ), in ppm.

In order to evaluate the acidity of the TPB derivatives and the basicity of TPP derivatives, several parameters were considered. Concretely, four parameters were used to calculate the acidity, namely, hydride affinity (HA), electroaccepting power ($\omega^{+}$), variation of the ${}^{31}$P NMR chemical shift (Δδ) and the boron empty orbital energy ($\varepsilon_{B}$). Similarly, the basicity of TPP derivatives were calculated by means of three parameters: proton affinity (PA), electrodonating power ($\omega^{-}$) and phosphorous lone-pair orbital energy ($\varepsilon_{P}$). The results are gathered in Table 1 and depicted in Figure 2.

First of all, the hydride affinity of TPB derivatives and proton affinity of TPP derivatives are analyzed as a function of the substituents. Hydride affinity is defined as the enthalpy change (ΔH) in the reaction between an acid (A) and a hydride anion (H${}^{-}$) in gas phase (see Equation (2)). Similarly, proton affinity is defined as the negative of enthalpy change (PA = −ΔH) in the reaction of a base (B) with a proton, taking into account the correction for the thermal energy of the proton as $\frac{5}{2}RT$ (see Equation (3)).

(2) $$A+H^{-}\to {\mathrm{AH}}^{-}$$

(3) $$B+H^{+}\to {\mathrm{BH}}^{+}$$

The numerical results are represented in Figure 2a. Regarding the hydride affinity, it can be observed that EDG-containing molecules show lower values than those with EWG substituents, while the reference molecule, formed by the unsubstituted TPB and TPP, shows an intermediate behavior. Since higher values of HA correspond to stronger acids, it can be concluded that EWG groups increase the acidity, while EDGs reduce it. Thus, the strongest acid is the CN-substituted TPB (HA = −150.90 kcal/mol). For R = SO${}_{3}$H, the most stable structure presents an intramolecular interaction between the empty orbital of boron with an oxygen of one of the SO${}_{3}$ groups, losing its acidic nature. It is remarkable the acidity of the TPB including acetoxy groups (OCOCH${}_{3}$), HA = −126.32 kcal/mol, in the same range of several EWG groups and even larger than that of the fluorine-containing TPB. The balance between σ-donor and π-acceptor character, as well as the coplanarity with the phenyl rings, makes OCOCH${}_{3}$ moiety a moderate EDG group, while fluorine can be considered both as a very weak EWG or a weak EDG group when is in the para position.

Contrary to hydride affinities, higher proton affinities are obtained for EDG substituents. Thus, the strongest Lewis bases correspond to TPP including amino (NH${}_{2}$) and methoxy (OCH${}_{3}$) groups (PA = 274.43 and 274.94 kcal/mol, respectively). These groups yielded the weakest TPB-based acids. The lowest value corresponds to CN group (199.95 kcal/mol) and, therefore, is the weakest base. Hence, according to hydride affinities of TPB and proton affinities of TPP, EWG lead to stronger acids and weaker bases, while EDG behave in the opposite way.

The acidity and basicity power may also be evaluated by means of the electroaccepting power ($\omega^{+}$) and electrodonating power ($\omega^{-}$) defined as in Equations (4) and (5) [83,84].

(4) $$\omega^{+}=\frac{A^{2}}{2(I-A)}$$

(5) $$\omega^{-}=\frac{I^{2}}{2(I-A)}$$

where *I* is the vertical ionization energy and *A* is the electron affinity of the considered species. Thus, larger $\omega^{+}$ values correspond to better electroaccepting power, which can be related to stronger acidity. Similarly, lower values of $\omega^{-}$ correspond to stronger basicity. The calculated values are represented in Figure 2b. Focusing on the electroaccepting power of TPB derivatives, one may observe that EWG substituents provide larger values than EDG substituents, in agreement with the hydride affinity. In the case of the TPB including NO${}_{2}$ groups, the calculation of the cation resulted in a molecule where one of the NO${}_{2}$ oxygens is attached to boron. Again, the strongest acid is the molecule substituted with CN groups. Regarding electrodonating power of TPP derivatives, it is observed that EDG groups present, in general, lower values, in agreement with the previously calculated proton affinities. However, the NH${}_{2}$-containing derivative shows a remarkably high value (3.792 eV), the largest value among all the calculated in this work, which is in disagreement with the trend observed in the proton affinity.

A third manner of considering the acidity and basicity power of different TPB and TPP derivatives is comparing their molecular orbital energies. Concretely, the acidity power may be related to the boron empty orbital energy that may accept electron density from a Lewis base. Lower energy values imply a more stable orbital and, therefore, better ability to accept electron density (stronger acidity). Besides, in TPP derivatives, the energy of the phosphorous lone-pair orbital ($\varepsilon_{P}$) may provide an insight into the basicity of these species. In Figure 2c, it is observed that, for boron orbital energies, EWG substituents yield to more negative values than EDG substitutents, following the same trend as the other parameters. Meanwhile, more positive phosphorus orbital energies values are observed for EDGs, which means that this orbital is more favorable to donate electron density and, therefore, these compounds are stronger electron donors (Lewis bases). One of the highest values corresponds to the amino group. Among the EWGs, the lowest value corresponds to cyanide, which would provide the weakest Lewis base, providing a similar picture as other previously considered parameters.

Finally, the acidity of TPB derivatives was calculated by means of the Gutmann–Beckett method [85], which is based in the variation of the ${}^{31}$P NMR chemical shift (Δδ) between free triethylphosphine oxide (Et${}_{3}$PO) and the adduct formed with a Lewis acid. The variation is caused by the interaction of the oxygen, which behaves as a Lewis base, with the Lewis acid, inducing a deshielding of the phosphorous atom and shifting Δδ to larger values. Thus, the larger is the shift, the greater is the Lewis acidity. The calculated values of Δδ are represented in Figure 2d. As expected, the same trend as in HA and $\omega^{+}$ is found, namely, EWG groups show larger variations of Δδ than the EDGs, and the largest shift is calculated for the CN-containing TBP. No stable Et${}_{3}$PO-LA adduct was found for the NO${}_{2}$ derivative.

Based on all these data, we may conclude that strong EDG groups would lead to stronger TPP Lewis basis and weaker TPB Lewis acid, while strong EWD would behave in the contrary way.

### 3.2. Interaction of Frustrated Lewis Pairs With DEAD

**Figure 3:**
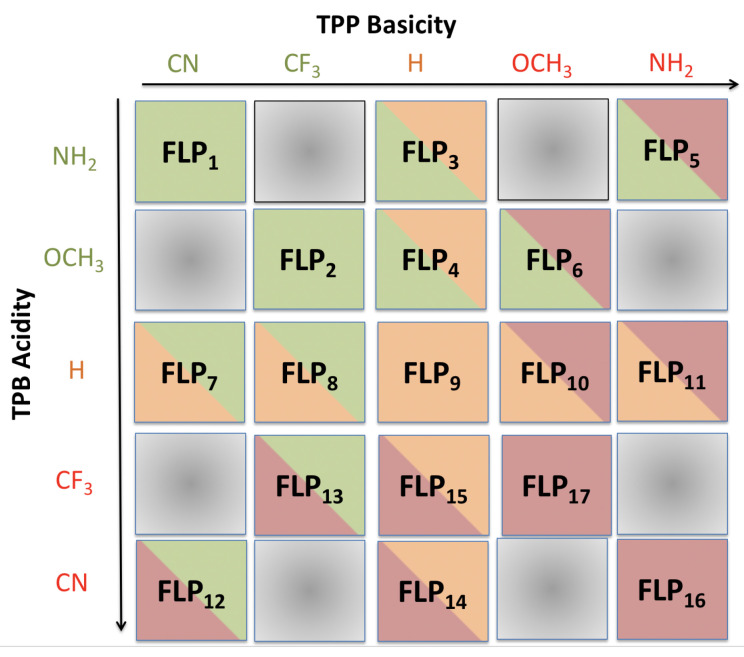
Combinations of the TPB- and TPP-derivatives to generate the 17 FLPs. Green, orange and red colors state for weak, moderate and strong acids and bases.

Once the acidity and basicity of the TBP and TPP derivatives have been evaluated, a set of frustrated Lewis pairs were defined to analyze their own interaction and the interaction with the DEAD molecule. In order to do so, first, the interaction enthalpies were calculated, and then the interaction nature was analyzed by means of natural bond orbital (NBO) and energy decomposition analysis (EDA). In particular, seventeen FLPs were studied, generated by the combination of five acids and five bases including the following substituents: CN and CF${}_{3}$ (strong acids and weak bases), H (moderate acid and base) and finally NH${}_{2}$ and OCH${}_{3}$ (weak acids and strong bases). The generated pairs are labeled in Figure 3. We are aware that the nucleophilic nitrogen of CN and the hydrogens of NH${}_{2}$ may react with Lewis acids and bases, respectively, destroying the FLP. Hence, the FLPs derived from the combination of these two substituents were calculated just for analysis purposes. Finally, the FLP used in the experimental material was also studied.

#### 3.2.1. Interaction Energies between FLPs and DEAD

In this subsection, both the interaction energy between the acids and bases to form the FLP (ΔH${}_{1}$) and between each FLP and the DEAD molecule (ΔH${}_{2}$) is studied. These interaction energies are defined as in Equation (6):

(6) $$\begin{array}{c} A+B\to \mathrm{AB}\;\Delta H_{1}\\ A+B+L\to \mathrm{ALB}\;\Delta H_{2} \end{array}$$

where ΔH${}_{1}$ = H${}_{\mathrm{AB}}$− (H${}_{A}$ + H${}_{B}$) and ΔH${}_{2}$ = H${}_{\mathrm{ALB}}$− (H${}_{A}$ + H${}_{B}$ + H${}_{L}$). H${}_{A}$, H${}_{B}$ and H${}_{L}$ correspond to the enthalpies of the acid, base and DEAD molecules isolated, H${}_{\mathrm{AB}}$ is the enthalpy of the FLP and H${}_{\mathrm{ALB}}$ is the enthalpy of the FLP–DEAD complex. The obtained results are collected in Table 2.

Let us focus first in the FLP interaction. In principle, frustrated Lewis pairs would show weak interaction and B-P distances longer than covalent values. Focusing on our reference, it can be seen that the B-P distance is 3.811 *Å*, with an interaction energy of −13.09 kcal/mol. Having these values as reference, it is observed that all ΔH${}_{1}$ values are in the range of −6 and −21 kcal/mol. Values larger than that of the reference correspond to FLP${}_{4}$, FLP${}_{5}$, FLP${}_{9}$ and FLP${}_{10}$. The last two show short bond distances, similar to those of covalent bonds. Hence, larger ΔH${}_{1}$ values (between −15 and −19 kcal/mol, approximately) are due to a low steric hindrance that allows an acid–base interaction, leading to regular, non-frustrated, LA-LB pairs. FLP${}_{5}$ shows by far the largest ΔH${}_{1}$ value, −21.41 kcal/mol, but with long B-P bond of 5.69 Å. This strong interaction is related to the hydrogen bond interaction between the amine groups of TPB and TPP derivatives. In FLP${}_{4}$, the B-P bond distance is slightly shorter and ΔH${}_{1}$ slightly larger than our reference, being the most similar FLP compared to our reference. The remaining FLPs show much larger B-P distances and smaller interaction energies, ranging between −6 and −11 kcal/mol, corresponding to weak non-covalent interactions between the bulky substituents and functional groups, indicative of very weakly interacting B-P pairs. Finally, it should be pointed out that we were not able to obtain the optimized structure of FLP${}_{16}$, despite several attempts. Based on these results, the FLP interaction energy is not directly related to the EDG or EWG nature of the substituent, but is more related to attractive or repulsive interactions between these substituents. Substituents such as amine or ciano groups may strongly interact with the neighbour groups or even B and P centers.

Regarding the interaction of the FLPs with DEAD molecule, we first focus on the reference model. According to the geometrical parameters given in Table 2, the linker molecule is located in between the Lewis acid and the Lewis base, leading to short B-N and P-N bond distances of 1.678 and 1.743, respectively. These bond lengths suggest the formation of dative covalent bonds between the linker and both Lewis acid and base. Moreover, the N-N bond length in the isolated linker is 1.243 Å, which is elongated to 1.425 Å in the complex, suggesting a change from double N=N to single N-N bond in the complex. The nature of these bonds are analyzed by means of NBO and EDA methods in the next subsection. The formation of the dative covalent bonds between the linker and the acid and base leads to an interaction energy, ΔH${}_{2}$ of −43.45 kcal/mol. Notice that this interaction energy is similar to others found in self-healing materials, like those based on diphenyl disulfide bonds [28].

Having a look at the results given in Table 2, we observe that several FLP–DEAD complexes are not formed. FLP${}_{9}$–DEAD and FLP${}_{10}$–DEAD have not been considered, since they are not frustrated Lewis pairs, as explained above. The combination of strong acids and strong bases, FLP${}_{1}$–DEAD and FLP${}_{2}$–DEAD, respectively, do not lead to converged structures. Finally, FLP${}_{13}$–DEAD optimized structure was not found. All attempts to minimize the optimal geometries of the mentioned FLP-linker complexes eventually failed, probably because of the combination of strong acids and bases is not favored in FLP${}_{1}$–DEAD and FLP${}_{2}$–DEAD complexes, and due to steric repulsions in FLP${}_{13}$–DEAD. Hence, hereafter only the remaining FLP–DEAD structures are considered for discussion.

Inspecting the B-N and P-N interatomic distances of optimized complexes provided in Table 2, apparently dative covalent bonds are formed between Lewis acids and Lewis bases with the linker with the exception of FLP${}_{3}$–DEAD and FLP${}_{5}$–DEAD complexes. In these two cases, B-N distances are too long for covalent bonds. The reason for this long interatomic distance is that the amine groups of the acid form hydrogen bonds with the DEAD linker, which are more favorable compared to the strong B-N interaction. Amine groups in the base are also able to form hydrogen bonds with DEAD, but in this case the formation of such bonds do not prevent the formation of B-N or P-N bonds. Finally, in the rest of FLP–DEAD complexes B-N, P-N and N-N distances similar to our reference species are found. Hence, the optimized complexes may be classified into three type of structures: (i) FLP–DEAD with only P-N covalent bond, and hydrogen bonds due to amine groups in the weak acid case, such as FLP${}_{3}$–DEAD and FLP${}_{5}$–DEAD complexes (ii) FLP–DEAD with B-N and P-N covalent bonds and hydrogen bonds, such as FLP${}_{11}$–DEAD and FLP${}_{16}$–DEAD complexes; (iii) FLP–DEAD with B-N and P-N covalent bonds but without hydrogen bonds, similar to the reference system bond pattern, for the remaining eight cases, namely: FLP${}_{n}$–DEAD, i = 4, 6, 7, 8, 12, 14, 15, 17.

Interaction energies of structures of type i and ii, which have hydrogen bonds between the amine substituents with the linker, are larger than the reference value. The formation of such hydrogen bonds is not desired in these materials, since they may prevent the correct function of the linker molecules. Hence, they should be discarded for the development of self healing materials. Hence, let us focus on the FLP–DEAD complexes of type iii, with the desired FLP–DEAD bond pattern. Among these complexes, we find the complexes with cyano groups. FLP${}_{14}$–DEAD complex, which contains the cyano group in the Lewis acid, is the strongest one, with ΔH${}_{2}$ value of −56.72 kcal/mol. This interaction energy is reduced drastically to −6.25 kcal/mol and 0.39 kcal/mol in the cases of FLP${}_{7}$–DEAD and FLP${}_{12}$–DEAD complexes, respectively, where the cyano group is found in the Lewis base (or in both like in FLP${}_{12}$–DEAD complex). Clearly, the influence of the EDG is very different in the Lewis acid or in the Lewis base, as expected from the results of Section 3.1. However, in addition to this influence, the lone pairs of cyano groups may lead to other non-desired interactions, such as the interaction with the empty orbital of the Lewis base, preventing in this way the proper interaction between the FLP and the linker. Hence, we no longer consider them moving forward. Among the remaining five complexes, only those containing methoxy groups as substituents in the Lewis acid have favorable ΔH${}_{2}$ values. The FLP${}_{4}$–DEAD complex is the most promising one, with an interaction energy of around −32 kcal/mol. The other remaining FLP–DEAD complexes have very weak or repulsive interactions, and hence must be discarded for the development of self-healing materials. In the next subsection, the bonding patterns of these five FLP–DEAD complexes are analyzed, in order to understand the electronic reasons for such different behaviors.

#### 3.2.2. Natural Bond Orbital (NBO) and Energy Decomposition Analysis (EDA)

In this subsection, a detailed analysis of the bonding pattern in FLP pairs, separated DEAD linker and the bonding between these FLPs and DEAD molecule is carried out by means of the NBO methodology. In Table 3, the calculated occupation numbers of key bonding orbitals and lone-pairs are given for FLPs and FLP–DEAD complexes. In addition, the EDA method, based on the energy decomposition scheme of Morokuma [79] was used to provide further information about the nature of the interactions between the FLPs and DEAD, in particular the acid–DEAD (B-N) and base–DEAD (P-N) interactions. As it was explained in the computational details, with this methodology, it is possible to obtain the electrostatic (ΔE${}_{elstat}$), exchange (ΔE${}_{Pauli}$) and covalent (ΔE${}_{orb}$) contributions to the total bonding energy for a specific interaction. Besides, the steric energy, or Heitler–London interaction energy, is defined as the sum of the Pauli repulsion and electrostatic attraction terms: ΔE${}_{steric}$ = ΔE${}_{Pauli}$ + ΔE${}_{elstat}$. A positive value of ΔE${}_{steric}$ is related to a covalent character of the interaction [86,87]. The results are collected in Table 4.

**Figure 4:**
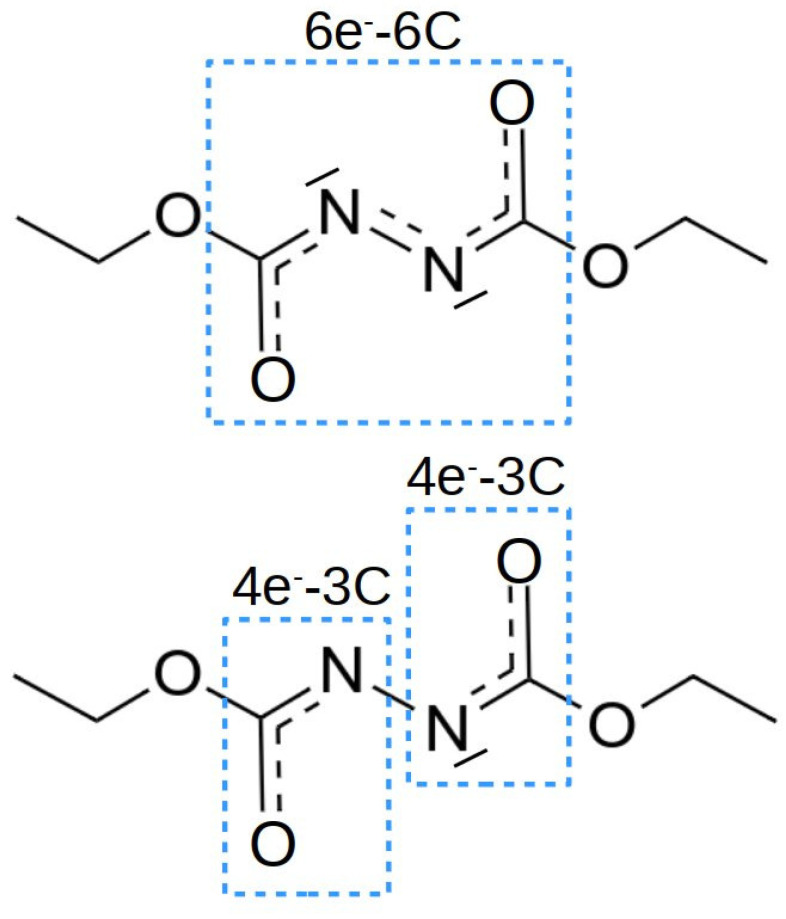
Electronic Lewis structure of the DEAD molecule isolated (top) and after complexation with the FLP (bottom).

NBO analysis provides localized orbitals that help in rationalizing the bonding within molecules and the interaction between different molecules. According to the Lewis acid and base nature, one would expect an empty orbital located at boron in the Lewis acid, and a lone-pair located at phosphorus, in the Lewis base. Inspecting the occupation numbers given in Table 3 for the the FLPs, it is observed that the occupation of the empty boron orbital, $LP_{\sigma }^{B}$, and of the lone-pair orbital in phosphorous, $LP_{\sigma }^{P}$, are slightly affected by the nature of the substituent in the benzyl rings. These electronic configurations lead to an almost trigonal planar geometry for the Lewis acid and a trigonal pyramidal geometry for the Lewis base. As expected, no $\sigma_{B-P}$ bond exists between the acid and the base and, therefore, weak interaction energies are calculated for the FLPs. Let us focus now on the electronic structure of the isolated DEAD species. The schematic representation of the electronic structure provided by NBO is depicted in the top of Figure 4. The isolated molecule shows a conjugated π system with six electrons in six centers. The carbonyl (C=O) and azo (N=N) groups possess a double bond, according to the localized bonds and orbitals provided by NBO. In addition, both nitrogens have lone pairs of σ symmetry perpendicular to the π system, in the directions where the Lewis acid and base should interact to form the complex, which may favor this interaction.

This electronic structure favors the interaction with the Lewis acid via a dative bond between the nitrogen lone pair and the boron empty orbital, but hinders the interaction with the lone pair of phosphorous. Hence, in order to form a stable complex, the electronic structure of the linker must be reorganized. The NBO analysis of the FLP–DEAD interactions show that, in this reorganization process, the two electrons of the nitrogen σ lone pair directed towards the phosphorous lone pair move to the π system, in such a way that this σ orbital now is empty and able to accept electron density from the P atom. The π system, therefore, now contains eight electrons and, as a consequence, the double bond in the azo group is broken, producing two 3-center 4-electron (3c-4e) systems, which resemble peptidic bonds (see Figure 4 bottom), and stabilizing the complex. This feature is clearly observed both in the elongation of the N-N bond distance from the isolated molecule, R${}_{e}$ = 1.254 Å, to the complex, in the range of 1.400–1.450 Å, and in the formation of B-N and N-P bonds, with bond distances between 1.600 and 1.800 Å (see Table 2).

According to this electronic reorganization, the interpretation of the interaction of Lewis acids and bases with DEAD molecule may be carried out. The occupation numbers of the formed B-N and P-N bonds, along with other relevant lone pairs is collected in Table 3. Focusing on the reference case, where both fragments show moderate acidity and basicity, the formation of covalent bonds is observed according to the occupation numbers of the bonding orbitals. Notice that no σ lone-pair orbitals are found for B and P. On the other hand, the π lone pairs are occupied in both N atoms. These orbitals are interacting with the carbonyl π system, leading to (3c-4e) bonds. The occupation numbers of all studied cases are very similar and are in agreement with calculated B-N, N-N and N-P bond distances in all complexes (see Table 2), which suggest that the type of bond in all cases is similar. Hence, from the NBO results we can conclude that, in general, the bonding interactions between the Lewis acid and Lewis bases and DEAD molecule are polar covalent bonds, with no clear influence of the substituents in the calculated occupation numbers that could explain the calculated differences in the interaction energies.

Energy decomposition analysis provides an alternative way to analyze the interaction of FLP–DEAD species. It is observed that all ΔE${}_{steric}$ values are positive and, therefore, all the interactions can be regarded as covalent. Inspecting the percentage contributions to the total attractive interactions (ΔE${}_{elstat}$ + ΔE${}_{orb}$ + ΔE${}_{disp}$) for the B-N bond, both the term related to the mixing of the orbitals (covalent character), ΔE${}_{orb}$, and the term corresponding to the electrostatic interaction, ΔE${}_{elstat}$, show similar values of around 40%. This means that the B-N bond is a polarized covalent bond in all complexes. Considering the P-N bond, remarkably higher values of the ΔE${}_{steric}$ term are calculated. This may indicate a larger covalent character of the P-N bond. Inspecting the percentage contributions to the total attractive interactions, the contribution of the ΔE${}_{orb}$ is also notably larger than the ΔE${}_{elstat}$ term in all cases, around 55% and 35%, respectively. Besides, the dispersion term is also lower. This suggests that the P-N bond is a less polarized covalent bond than B-N.

## 4. Conclusions

In this work, we have computationally studied complexes of several frustrated Lewis pairs with a small molecule (diethyl azodicarboxylate, DEAD) as potential candidates for new dynamic bonds useful in self-healing materials. Inspired by the experimental work of Shaver and coworkers [54], we have designed a set of FLPs using Lewis acids and bases based on triphenylborane (TPB) and triphenylphosphine (TPP), in order to understand the nature of the interaction with the DEAD molecule and the influence of the substituents in the acid and the base.

First of all, the acidity and basicity of the isolated TPB and TPP derivatives have been analyzed by means of different parameters. For Lewis acids, hydride affinity (HA), electroaccepting power ($\omega^{+}$), variation of the ${}^{31}$P NMR chemical shift (Δδ) and boron empty orbital energy ($\epsilon_{B}$) were analyzed, while for Lewis bases the studied criteria were proton affinity (PA), electrodonating power ($\omega^{-}$) and phosphorous lone-pair orbital energy ($\epsilon_{P}$). For the acids, all criteria show that electron withdrawing substituents in the phenyl rings lead to stronger Lewis acids, while for bases, electron-donating groups were those leading to stronger Lewis bases.

From the previous set, three TPB and TPP derivatives corresponding to strong, moderate and weak acids and bases were chosen and combined to generate 17 FLPs, together with the reference from the experimental work. The acid–base interaction energy to form the FLP (ΔH${}_{1}$) as well the interaction of all the FLPs with DEAD (ΔH${}_{2}$) were calculated. Based on the obtained structures and interaction energies, substituents with the capacity to form hydrogen bonds or donor–acceptor bonds should be discarded, since they can easily break the proper FLP–DEAD interaction.

Finally, both NBO and EDA analyses provide complementary pictures of the interaction within FLP–DEAD patterns. According to NBO, the DEAD species undergoes an electronic configuration rearrangement, so that dative covalent bonds are formed between TPB–DEAD and TPP–DEAD fragments, leading to polar covalent bonds. The polarity of these bonds is observed to be larger for the formed B-N bond rather than the P-N bond. Compared to the reference system, similar bond nature are calculated for the rest of the systems, like in the NBO analysis. These small differences between these complexes cannot explain the large calculated differences in FLP–DEAD interaction energies. Hence, these differences may be attributed to the steric repulsion between substituents, and not to substantial changes in the bond patterns. Concretely, in this work only FLP${}_{4}$-DEAD system is proposed to be a real alternative to the reference material. Other alternatives, such as linker substitution or the use of other type of FLPs should be considered for further improvements.

## Figures and Tables

**Table 1:** Acidity of triphenylborane (TPB) and basicity of triphenylphosphine (TPP) substituted with electron-donating (EDG) and electron withdrawing (EWG) groups (R). Acidity is estimated by hydride affinity (HA), in kcal/mol, electroaccepting power ($\omega^{+}$), in eV, variation of the ${}^{31}$P NMR chemical shift (Δδ), in ppm and boron empty orbital energy ($\varepsilon_{B}$), in a.u. Basicity is estimated by proton affinity (PA), in kcal/mol, electrodonating power ($\omega^{-}$), in eV and phosphorus lone-pair orbital energy ($\varepsilon_{P}$), in a.u.

	R	TPB acidity				TPP basicity		
		**HA**	$\omega^{+}$	Δδ	$\varepsilon_{B}$	**PA**	$\omega^{-}$	$\varepsilon_{P}$
	H	−90.48	0.049	35.28	−0.0948	240.27	3.293	−0.1925
	CH${}_{3}$	−87.42	0.056	16.04	−0.0885	253.88	2.957	−0.1690
EDG	NH${}_{2}$	−77.66	0.007	20.15	−0.0610	274.43	3.792	−0.1435
	OH	−74.67	0.002	16.39	−0.0602	267.92	2.555	−0.1504
	OCH${}_{3}$	−73.89	0.003	25.35	−0.0547	274.94	2.944	−0.1421
	OCOCH${}_{3}$	−126.32	0.321	43.85	−0.1385	232.98	2.847	−0.2160
	F	−109.56	0.130	32.10	−0.1195	228.16	3.360	−0.2130
	5F ^*a*^	−125.43	0.248	36.34	−0.1452	212.87	3.364	−0.2363
	CF${}_{3}$	−127.89	0.337	44.21	−0.1595	218.54	2.992	−0.2321
EWG	CN	−150.90	0.542	47.56	−0.1935	199.95	3.126	−0.2636
	NO${}_{2}$	−138.57		—	−0.1229	202.58	—	−0.2562
	SO${}_{3}$H	—	—	—	—	212.11	2.810	−0.2517
Reference		−89.45	0.034	35.13	−0.0913	252.13	3.132	−0.1736

The reference system corresponds to the unsubstituted TPB and TPP. *^a^* 5F stands for fluorine substitution in all carbons of the aromatic ring.

**Table 2:** Interaction energies between Lewis acids and bases to form the FLPs (ΔH${}_{1}$) and between the FLPs and DEAD molecule (ΔH${}_{2}$), in kcal/mol. Bond distances (R${}_{e}$), in Å. Red, orange and green groups stand for strong, moderate and weak acids or bases, respectively.

	Acid	Base	FLP		FLP–DEAD			
			**ΔH${}_{1}$**	**R${}_{e}$(B-P)**	**ΔH${}_{2}$**	**R${}_{e}$(B-N)**	**R${}_{e}$(N-N)**	**R${}_{e}$(N-P)**
FLP${}_{1}$	NH${}_{2}$	CN	−8.27	5.691	—	—	—	—
FLP${}_{2}$	OCH${}_{3}$	CF${}_{3}$	−9.33	6.396	—	—	—	—
FLP${}_{3}$	NH${}_{2}$	H	−10.81	5.861	−43.54	3.586	1.416	1.703
FLP${}_{4}$	OCH${}_{3}$	H	−15.74	3.430	−32.37	1.700	1.414	1.721
FLP${}_{5}$	NH${}_{2}$	NH${}_{2}$	−21.41	5.687	−48.58	4.176	1.432	1.731
FLP${}_{6}$	OCH${}_{3}$	OCH${}_{3}$	−11.40	5.860	−4.95	1.709	1.436	1.763
FLP${}_{7}$	H	CN	−9.23	5.455	−6.25	1.702	1.421	1.718
FLP${}_{8}$	H	CF${}_{3}$	−6.09	5.689	28.18	1.723	1.425	1.722
FLP${}_{9}$	H	H	−18.90 ^*a*^	2.112	—	—	—	—
FLP${}_{10}$	H	OCH${}_{3}$	−14.95 ^*a*^	2.267	—	—	—	—
FLP${}_{11}$	H	NH${}_{2}$	−10.80	5.418	−57.47	1.694	1.428	1.789
FLP${}_{12}$	CN	CN	−9.58	7.171	0.39	1.663	1.450	1.786
FLP${}_{13}$	CF${}_{3}$	CF${}_{3}$	−8.95	7.820	—	—	—	—
FLP${}_{14}$	CN	H	−9.31	5.686	−56.72	1.647	1.433	1.752
FLP${}_{15}$	CF${}_{3}$	H	−9.25	6.123	14.13	1.654	1.437	1.814
FLP${}_{16}$	CN	NH${}_{2}$	—	—	−50.87	1.646	1.446	1.879
FLP${}_{17}$	CF${}_{3}$	OCH${}_{3}$	−9.29	6.212	26.17	1.687	1.448	1.877
Reference			−13.09	3.811	−43.45	1.678	1.425	1.743

^*a*^ Regular acid–base pair (not FLP).

**Table 3:** Orbital occupancies of empty boron (LP${}_{\sigma }^{B}$) and phosphorous lone-pair (LP${}_{\sigma }^{P}$) orbitals of the FLPs. In addition, occupancies of selected bonding orbitals of the FLP–DEAD complexes ($\sigma_{B-N}$ and $\sigma_{N-P}$), along with selected lone pairs of boron (LP${}_{\sigma }^{B}$) and nitrogen atom bonded to boron (LP${}_{\sigma }^{N(B)}$, LP${}_{\pi }^{N(B)}$) and phosphorous (LP${}_{\pi }^{N(P)}$). Red, orange and olive groups stand for strong, moderate and weak acids or bases, respectively.

	Acid	Base	FLP		FLP–DEAD			
			**LP** ${}_{\sigma }^{B}$	**LP** ${}_{\sigma }^{P}$	$\sigma_{\mathrm{BN}}$	$\sigma_{\mathrm{NP}}$	**LP** ${}_{\pi }^{N(B)}$	**LP** ${}_{\pi }^{N(P)}$
FLP${}_{4}$	OCH${}_{3}$	H	0.302	1.774	1.958	1.965	1.666	1.722
FLP${}_{6}$	OCH${}_{3}$	OCH${}_{3}$	0.283	1.844	1.955	1.959	1.667	1.729
FLP${}_{8}$	H	CF${}_{3}$	0.271	1.781	1.949	1.959	1.630	1.732
FLP${}_{15}$	CF${}_{3}$	H	0.212	1.870	1.964	1.948	1.690	1.774
FLP${}_{17}$	CF${}_{3}$	OCH${}_{3}$	0.235	1.855	1.953	1.937	1.690	1.776
Reference			0.282	1.810	1.933	1.962	1.690	1.740

**Table 4:** Interaction energy (ΔE${}_{int}$), Pauli repulsion (ΔE${}_{Pauli}$), electrostatic interaction (ΔE${}_{elstat}$), steric energy (ΔE${}_{st}$), orbital attraction (ΔE${}_{orb}$) and dispersion energy (ΔE${}_{disp}$), in kcal/mol, for the B-N (acid–DEAD) and P-N (base–DEAD) bonds. Values in brackets are the percentage contributions to the total attractive interactions: ΔE${}_{elstat}$ + ΔE${}_{orb}$ + ΔE${}_{disp}$.

	Acid	Base	ΔE${}_{\mathrm{int}}$	ΔE${}_{\mathrm{Pauli}}$	ΔE${}_{\mathrm{elstat}}$	ΔE${}_{\mathrm{st}}$^*a*^	ΔE${}_{\mathrm{orb}}$	ΔE${}_{\mathrm{disp}}$
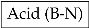								
FLP${}_{4}$	OCH${}_{3}$	H	−68.05	215.88	-118.41 (41.7%)	97.47	−114.11 (40.2%)	−51.44 (18.2%)
FLP${}_{6}$	OCH${}_{3}$	OCH${}_{3}$	−67.68	211.41	−109.07 (39.1%)	102.34	−116.10 (41.6%)	−53.95 (19.3%)
FLP${}_{8}$	H	CF${}_{3}$	−55.75	161.49	−92.39 (42.5%)	69.10	−88.28 (40.6%)	−36.58 (16.8%)
FLP${}_{15}$	CF${}_{3}$	H	−96.11	229.26	−139.18 (42.8%)	90.08	−138.56 (42.6%)	−47.64 (14.6%)
FLP${}_{17}$	CF${}_{3}$	OCH${}_{3}$	−107.17	216.50	−133.17 (41.1%)	83.34	−138.50 (42.8%)	−52.02 (16.1%)
Reference			−73.34	175.17	−107.01 (43.1%)	68.16	−105.35 (42.4%)	−36.16 (14.6%)
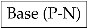								
FLP${}_{4}$	OCH${}_{3}$	H	−119.91	592.84	−282.42 (39.6%)	310.43	−393.95 (55.2%)	−36.39 (5.1%)
FLP${}_{6}$	OCH${}_{3}$	OCH${}_{3}$	−86.04	521.46	−235.17 (38.7%)	286.29	−327.17 (53.9%)	−45.19 (7.4%)
FLP${}_{8}$	H	CF${}_{3}$	−86.68	636.17	−303.47 (41.9%)	332.70	−376.63 (52.1%)	−42.77 (5.9%)
FLP${}_{15}$	CF${}_{3}$	H	−134.95	485.87	−230.11 (37.1%)	255.76	−357.59 (57.6%)	−33.13 (5.4%)
FLP${}_{17}$	CF${}_{3}$	OCH${}_{3}$	−109.05	408.32	−188.80 (36.5%)	219.51	−283.62 (54.8%)	−44.97 (8.7%)
Reference			−139.90	549.10	−256.80 (37.3%)	292.30	−396.57 (57.6%)	−35.65 (5.2%)

^*a*^ΔE${}_{st}$ = ΔE${}_{Pauli}$ + ΔE${}_{elstat}$

## Supplementary Materials

The Cartesian coordinates of all optimized structures are available online at https://www.mdpi.com/article/10.3390/polym13101573/s1.
