# Supplementary material for: Theoretical Characterization of New Frustrated Lewis Pairs for Responsive Materials
Source: Polymers (Basel). 2021 May 14;13(10):1573. doi: 10.3390/polym13101573 (PMC8155995; doi:10.3390/polym13101573)
Supplement: Supplementary file 1 [file polymers-13-01573-s001.zip › polymers-1219144-supplementary.pdf]

Supporting information for

Theoretical characterization of new dynamic bonds for responsive materials based on frustrated Lewis pairs

Maialen Galdeano,[1] Fernando Ruipérez[1,*] and Jon M. Matxain[2,*]

[1]POLYMAT, University of the Basque Country UPV/EHU,
Jose Mari Korta Center, Avda. Tolosa 72, 20018 Donostia – San Sebastián, Spain
[2]Polimero eta Material Aurreratuak: Fisika, Kimika eta Teknologia Saila, Kimika Fakultatea,
Euskal Herriko Unibertsitatea UPV/EHU and Donostia International Physics Center (DIPC),
P.K. 1072, 20080 Donostia, Euskadi, Spain

email: jonmattin.matxain@ehu.eus; fernando.ruiperez@polymat.eu

Content: cartesian coordinates of all species, along with the calculated total energies and enthalpy values. All structures are minima of the Potential Energy Surface, and hence no imaginary frequencies are reported.

Index

1. TPB derivatives. Cartesian coordinates……………………………….1
2. TPP derivatives. Cartesian coordinates……………………………….41
3. FLP cartesian coordinates……………………………………………….66
4. FLP-DEAD cartesian coordinates……………………………………….98

1. TPB derivatives. Cartesian coordinates

1. TPB. R = CF_3
E_{elec} = -3755.179087 a.u.; **H =** -3754.822177 a.u.

C	-3.276526	-3.031922	0.011280
C	-3.325704	-1.948150	-0.851908
C	-2.301459	-1.001775	-0.860048
C	-1.176335	-1.091179	0.008839
C	-1.174450	-2.219117	0.878373
C	-2.198056	-3.166564	0.871722
B	0.000285	-0.000315	0.009424
C	1.533352	-0.473797	0.009368
C	2.019010	-1.493114	-0.858918
C	3.350945	-1.906269	-0.850677
C	4.264801	-1.320482	0.011716
C	3.841787	-0.318645	0.871384
C	2.509150	0.093062	0.878296
C	-0.356397	1.563939	0.009123
C	0.283325	2.493978	-0.859450
C	-0.024734	3.854142	-0.851123
C	-0.988483	4.352858	0.011646
C	-1.644325	3.485715	0.871676
C	-1.334830	2.125769	0.878382
C	1.352434	2.134140	-1.890434
C	-2.141617	1.338155	1.910034
H	0.489376	4.526539	-1.526909
H	-2.389797	3.871382	1.556150

C	-1.351406	5.818649	-0.018295
C	1.172505	-2.239149	-1.889532
C	2.230653	1.185569	1.910006
H	3.676205	-2.687916	-1.526226
H	4.548580	0.134416	1.555596
C	5.715597	-1.739359	-0.018427
C	-2.523341	0.104354	-1.890810
C	-0.089522	-2.524196	1.910474
H	-4.164799	-1.838905	-1.528025
H	-2.159967	-4.004754	1.556525
C	-4.365424	-4.078123	-0.018899
F	0.162291	-1.507338	-2.407809
F	0.619417	-3.369064	-1.371368
F	1.928842	-2.636347	-2.946744
F	5.857050	-3.041361	-0.369780
F	6.315276	-1.571914	1.185806
F	6.420107	-1.003890	-0.920995
F	2.411360	2.430695	1.391678
F	0.983704	1.151784	2.428131
F	3.078974	1.086964	2.967345
F	0.897964	-3.304281	1.392812
F	0.505478	-1.427377	2.427855
F	-0.599705	-3.208442	2.968189
F	-5.564079	-3.547993	-0.366677
F	-4.518137	-4.683720	1.184288
F	-4.083251	-5.054160	-0.924216
F	-3.245546	-0.351527	-2.948181
F	-3.224615	1.148745	-1.372651
F	-1.383997	0.612683	-2.408840
F	-3.309893	0.871374	1.391494
F	-1.488308	0.275449	2.428249
F	-2.481034	2.121900	2.967253
F	2.607614	2.222292	-1.372992
F	1.225334	0.892745	-2.407590
F	1.316483	2.986631	-2.948543
F	-0.295667	6.592289	-0.372560
F	-1.793462	6.254582	1.186866
F	-2.342947	6.060378	-0.918464

2. TPB. R = CH_3

E_{elec} = -1074.270401 a.u.; **H =** -1073.725803 a.u.

C	-0.435826	-4.403091	-0.003143
C	0.408936	-3.761404	0.908233
C	0.574027	-2.375611	0.912065
C	-0.156207	-1.567101	0.000077
C	-1.028415	-2.213710	-0.911941
C	-1.137039	-3.608006	-0.910760
B	0.000045	0.000328	0.000760
C	-1.279537	0.919100	0.000266
C	-2.344452	0.691067	0.912749
C	-3.462378	1.526528	0.908755
C	-3.596397	2.578348	-0.003155
C	-2.557397	2.788228	-0.910926
C	-1.403923	1.997462	-0.911958
C	1.435753	0.648728	-0.000069

C	1.771400	1.684708	0.912435
C	3.054304	2.234159	0.908594
C	4.031995	1.823737	-0.003351
C	3.693397	0.819691	-0.911518
C	2.431431	0.216978	-0.912615
C	0.778839	2.209235	1.930261
C	2.171753	-0.875848	-1.929871
H	3.297158	3.009916	1.634280
H	4.435304	0.490222	-1.638037
C	5.405326	2.450228	0.000531
C	-2.301657	-0.430436	1.930998
C	-0.327274	2.319806	-1.928476
H	-4.255658	1.348362	1.634251
H	-2.643329	3.595687	-1.637160
C	-4.826373	3.453403	0.000348
C	1.525026	-1.777945	1.929267
C	-1.846105	-1.442650	-1.928492
H	0.960056	-4.359434	1.633496
H	-1.793789	-4.085917	-1.636748
C	-0.579370	-5.905731	0.000743
H	-3.074908	-0.285310	2.692070
H	-2.466562	-1.404595	1.457827
H	-1.333811	-0.489286	2.439860
H	-4.768830	4.223473	-0.774700
H	-5.732303	2.860555	-0.175441
H	-4.950757	3.953613	0.968496
H	-0.715478	2.999443	-2.693759
H	0.537009	2.797780	-1.454291
H	0.047949	1.422945	-2.432122
H	1.291253	2.806238	2.691330
H	0.018279	2.839498	1.456534
H	0.243220	1.401011	2.439192
H	2.954426	-0.879140	-2.695165
H	2.153332	-1.863721	-1.456502
H	1.207528	-0.751712	-2.433546
H	6.043192	2.015048	-0.774552
H	5.345826	3.531319	-0.174908
H	5.900417	2.307068	0.968714
H	-2.240467	-2.118892	-2.693639
H	-2.692345	-0.933220	-1.454536
H	-1.257175	-0.669302	-2.432357
H	1.786231	-2.519945	2.690467
H	2.450863	-1.434522	1.454948
H	1.093074	-0.909777	2.438015
H	-1.275776	-6.240777	-0.773715
H	0.386657	-6.394358	-0.175645
H	-0.949817	-6.263149	0.969248

3. TPB. R = CN

E_{elec} = -1550.838094 a.u.; **H =** -1550.549129 a.u.

C	3.978715	1.902343	-0.000263
C	3.714007	0.833153	-0.865517
C	2.456801	0.222468	-0.847370
C	1.415721	0.676978	0.000272
C	1.715947	1.772734	0.847683

C	2.980639	2.367782	0.865289
B	-0.000065	0.000132	0.000338
C	-1.294159	0.887707	0.000322
C	-1.421125	2.016892	-0.846894
C	-2.578779	2.800059	-0.864954
C	-3.637237	2.494075	-0.000090
C	-3.541282	1.396622	0.864969
C	-2.393408	0.599189	0.847288
C	-0.121650	-1.564391	0.000183
C	-1.035558	-2.238954	-0.847556
C	-1.135066	-3.633083	-0.865576
C	-0.341420	-4.396706	-0.000134
C	0.560495	-3.764845	0.865460
C	0.677264	-2.372054	0.847728
H	-1.826898	-4.120139	-1.543626
H	1.168909	-4.352939	1.543387
H	-2.654462	3.643037	-1.542645
H	-4.355070	1.163348	1.542420
H	4.481649	0.477310	-1.543531
H	3.185909	3.188790	1.543090
C	-0.362994	2.364949	-1.747517
N	0.493738	2.659446	-2.475462
C	-4.816317	3.302375	-0.000353
N	-5.774527	3.959243	-0.000570
C	-2.336838	-0.513296	1.747893
N	-2.302905	-1.418566	2.475872
C	0.724544	2.279698	1.748793
C	5.268413	2.518992	-0.000544
C	2.228659	-0.867581	-1.748286
N	2.055189	-1.756560	-2.476462
N	6.316499	3.020146	-0.000745
N	-0.076265	2.702845	2.476990
C	1.611848	-1.766775	1.748893
C	-1.865399	-1.496505	-1.748682
C	-0.451993	-5.821961	-0.000327
N	2.378472	-1.284642	2.477220
N	-0.541859	-6.980221	-0.000503
N	-2.548154	-0.901586	-2.477062

4. TPB. R = 5F

E_{elec} = -2209.480848 a.u.; **H =** -2209.300739 a.u.

C	-1.768735	1.763723	-0.731042
C	-1.441899	0.610328	0.000182
C	-2.497387	0.041832	0.731211
C	-3.780619	0.575266	0.753887
C	-4.055867	1.716389	0.000038
C	-3.045106	2.313320	-0.753847
B	-0.000247	0.000210	0.000019
C	0.192305	-1.553390	0.000034
C	1.211915	-2.183095	0.731891
C	1.391714	-3.561070	0.754722
C	0.541762	-4.370124	0.000222
C	-0.480134	-3.793411	-0.754349
C	-0.642545	-2.413238	-0.731705
F	2.053465	-1.448063	1.488033

F	0.706192	-5.695817	0.000355
F	-1.638200	-1.906184	-1.487878
F	-2.282031	-1.055193	1.486407
F	-5.286145	2.236870	-0.000095
F	-0.831461	2.372748	-1.486587
C	1.249049	0.943592	-0.000222
C	1.285347	2.141236	0.731951
C	2.389223	2.985426	0.754680
C	3.514437	2.653643	-0.000362
C	3.525157	1.480472	-0.755214
C	2.410793	0.650190	-0.732318
F	4.580656	3.458435	-0.000360
F	0.228507	2.502628	1.488723
F	2.468904	-0.465658	-1.488436
F	-4.752220	0.009830	1.485417
F	-3.315799	3.404277	-1.485574
F	-1.289001	-4.573439	-1.486581
F	2.366606	-4.119688	1.487112
F	4.604773	1.169609	-1.487791
F	2.386286	4.108803	1.487407

5. TPB. R = F

E_{elec} = -1613.835736 a.u.; **H =** -1613.613252 a.u.

C	0.647979	4.338789	-0.000282
C	-0.394641	3.822837	0.758697
C	-0.571523	2.448058	0.721817
C	0.231101	1.547311	0.000169
C	1.261890	2.174017	-0.721627
C	1.494387	3.540470	-0.758978
B	-0.000077	-0.000118	0.000356
C	1.224445	-0.974017	0.000341
C	2.405622	-0.729394	0.722356
C	3.507852	-1.569866	0.759246
C	3.433702	-2.730530	-0.000165
C	2.319483	-3.064129	-0.759439
C	1.252249	-2.179665	-0.722103
C	-1.455811	-0.573597	0.000296
C	-1.834793	-1.718423	0.722709
C	-3.113863	-2.252564	0.759609
C	-4.081759	-1.608158	-0.000151
C	-3.813314	-0.476651	-0.759726
C	-2.513657	0.005183	-0.722376
H	-3.344002	-3.128410	1.353430
H	-4.578853	0.006886	-1.353726
H	4.381570	-1.330943	1.352730
H	2.283539	-3.968967	-1.353297
H	-1.038538	4.460108	1.351973
H	2.296428	3.961631	-1.352316
F	4.491456	-3.571468	-0.000449
F	2.480966	0.394848	1.477233
F	0.174098	-2.505974	-1.477420
F	2.084159	1.403138	-1.475957
F	0.847604	5.675266	-0.000427
F	-1.582896	1.951399	1.476712
F	-2.256929	1.102028	-1.477678

F	-0.898995	-2.345473	1.478040
F	-5.339001	-2.103513	-0.000380

6. TPB. R = H

E_{elec} = -720.230338 a.u.; **H =** -719.941721 a.u.

B	0.000003	-0.000024	0.001862
C	-1.469505	-0.540172	0.000896
C	-2.511168	0.159767	-0.646447
C	-1.810712	-1.748418	0.647226
C	-3.817368	-0.325609	-0.662160
H	-2.282401	1.091408	-1.158619
C	-3.120375	-2.224445	0.661180
H	-1.033680	-2.310368	1.160102
C	-4.126176	-1.516600	-0.000930
H	-4.596795	0.224544	-1.183488
H	-3.358634	-3.148637	1.181813
H	-5.146616	-1.891657	-0.001648
C	0.266938	1.542703	0.000783
C	1.394044	2.094901	-0.646325
C	-0.608992	2.442295	0.646947
C	1.626816	3.468791	-0.661879
H	2.086529	1.431011	-1.158493
C	-0.366426	3.814511	0.661022
H	-1.484297	2.050333	1.159598
C	0.749649	4.331676	-0.000779
H	2.493054	3.868762	-1.183043
H	-1.047776	4.482917	1.181555
H	0.935069	5.402930	-0.001366
C	1.202532	-1.002597	0.000861
C	1.117145	-2.254861	-0.646129
C	2.419553	-0.693823	0.647048
C	2.190683	-3.143268	-0.661912
H	0.195873	-2.522741	-1.158098
C	3.486732	-1.589892	0.660923
H	2.517710	0.260143	1.159798
C	3.376628	-2.814961	-0.000994
H	2.103951	-4.093440	-1.183073
H	4.406297	-1.333982	1.181373
H	4.211712	-3.511094	-0.001711

7. TPB. R = NH_2

E_{elec} = -1218.755348 a.u.; **H =** -1218.307758 a.u.

C	1.932823	-3.994693	0.005971
C	2.432896	-2.970647	0.818186
C	1.829692	-1.706308	0.815152
C	0.687536	-1.415084	-0.003355
C	0.209423	-2.494852	-0.817950
C	0.824130	-3.753140	-0.813278
B	-0.000053	0.000014	-0.004554
C	-1.569499	0.111831	-0.003445
C	-2.392766	-0.730961	0.815810
C	-3.789299	-0.620957	0.818899
C	-4.426054	0.323357	0.005771
C	-3.662480	1.161995	-0.814221
C	-2.265371	1.065313	-0.818567

C	0.881470	1.303105	-0.003551
C	0.562445	2.438283	0.814477
C	1.356055	3.592668	0.817392
C	2.493434	3.671046	0.005856
C	2.838738	2.589794	-0.812880
C	2.056050	1.428564	-0.817680
N	-0.497191	2.375046	1.707244
N	2.386799	0.424357	-1.717876
H	1.104529	4.418463	1.480719
H	3.709295	2.656833	-1.463162
N	3.315896	4.797499	0.060313
N	-1.807632	-1.615701	1.709562
N	-1.560827	1.853141	-1.719299
H	-4.378542	-1.250768	1.483226
H	-4.155986	1.881391	-1.465493
N	-5.812890	0.472156	0.059905
N	2.303996	-0.756764	1.708060
N	-0.825148	-2.279138	-1.718681
H	3.273490	-3.165707	1.481929
H	0.447507	-4.540457	-1.464058
N	2.497528	-5.270049	0.060239
H	3.354017	0.402072	-2.014394
H	1.999371	-0.492438	-1.511442
H	-0.864714	3.263241	2.022437
H	-1.210405	1.681922	1.498880
H	3.890004	4.954069	-0.759528
H	2.864995	5.646687	0.379488
H	3.256919	-0.882073	2.023556
H	2.059936	0.207317	1.499574
H	-1.327315	-3.105789	-2.016019
H	-1.426179	-1.485899	-1.511834
H	2.346685	-5.845166	-0.759975
H	3.458309	-5.303879	0.379725
H	-2.392870	-2.377652	2.026182
H	-0.851018	-1.887274	1.500594
H	-2.025295	2.701515	-2.016745
H	-0.573394	1.976855	-1.512101
H	-6.235495	0.889645	-0.760682
H	-6.322633	-0.342676	0.380093

8. TPB. R = NO_2

E_{elec} = -2561.838074 a.u.; **H =** -2561.511658 a.u.

C	-3.257096	-3.049239	0.000822
C	-3.389019	-1.915954	-0.783038
C	-2.346782	-0.997486	-0.782497
C	-1.175689	-1.101348	-0.000136
C	-1.149006	-2.276565	0.782365
C	-2.134493	-3.255679	0.783985
B	0.000183	-0.000157	-0.000697
C	1.541868	-0.467628	-0.000786
C	2.037913	-1.532825	-0.784086
C	3.354534	-1.975940	-0.784768
C	4.269605	-1.295693	0.000160
C	3.886615	-0.221252	0.784435
C	2.545879	0.142425	0.782801

C	-0.365766	1.568691	-0.000248
C	0.310999	2.531487	-0.780827
C	0.036246	3.893216	-0.781442
C	-1.012994	4.344882	0.000431
C	-1.754174	3.475369	0.782014
C	-1.398408	2.132520	0.780781
N	1.374998	2.119467	-1.733512
N	-2.170262	1.291978	1.733226
H	0.613620	4.575269	-1.393402
H	-2.573566	3.831471	1.394261
N	-1.350625	5.793092	0.000796
N	1.149689	-2.244757	-1.739813
N	2.203075	1.228477	1.738053
H	3.656930	-2.815128	-1.399078
H	4.604104	0.308339	1.399084
N	5.692518	-1.727821	0.000894
N	-2.520011	0.128384	-1.737470
N	-0.036282	-2.523681	1.736332
H	-4.267400	-1.757584	-1.396563
H	-2.034208	-4.142420	1.397729
N	-4.342952	-4.065277	0.001463
O	1.464513	-3.383285	-2.078019
O	0.155363	-1.633597	-2.141746
O	6.465491	-1.099709	0.723015
O	5.986276	-2.680094	-0.720463
O	3.097513	2.000559	2.075121
O	1.037047	1.272889	2.140710
O	0.186574	-3.684866	2.070669
O	0.584210	-1.536274	2.140905
O	-3.663792	0.425583	-2.073887
O	-1.493983	0.683701	-2.140786
O	-4.185336	-5.049159	0.723000
O	-5.314800	-3.842993	-0.719351
O	2.207831	2.960622	-2.063192
O	1.341055	0.955104	-2.141880
O	-3.289462	1.677630	2.062676
O	-1.624570	0.262833	2.141475
O	-2.283130	6.147944	0.720528
O	-0.671152	6.524099	-0.718541

9. TPB. R = OCH_3

E_{elec} = -1751.534491 a.u.; **H =** -1750.936926 a.u.

C	2.443797	-3.671111	0.069522
C	2.816638	-2.593515	0.876639
C	2.034668	-1.437984	0.858141
C	0.871458	-1.305247	0.064171
C	0.547885	-2.426022	-0.723579
C	1.309780	-3.604403	-0.741140
B	-0.001253	0.000302	0.062763
C	-1.568062	-0.102360	0.063527
C	-2.265280	-1.046028	0.854048
C	-3.657005	-1.144825	0.872126
C	-4.403406	-0.279886	0.068112
C	-3.778163	0.671466	-0.739075
C	-2.376695	0.741445	-0.721262

C	0.694162	1.408253	0.062433
C	0.228645	2.483635	0.854944
C	0.840919	3.737360	0.873206
C	1.961866	3.950451	0.067121
C	2.469807	2.933510	-0.742400
C	1.827707	1.685793	-0.724671
O	-0.852057	2.200825	1.649812
O	2.254380	0.657898	-1.527922
H	0.493679	4.550445	1.498448
H	3.324757	3.106955	-1.381150
O	2.503859	5.211165	0.138699
O	-1.479018	-1.840850	1.647508
O	-1.701537	1.628175	-1.522181
H	-4.186351	-1.854260	1.495905
H	-4.356823	1.325782	-1.376297
O	-5.765912	-0.443113	0.139323
O	2.326569	-0.360731	1.654460
O	-0.555561	-2.283577	-1.527331
H	3.693802	-2.697930	1.502975
H	1.034679	-4.431796	-1.380495
O	3.266694	-4.769280	0.141464
C	-2.102502	-2.873160	2.406073
H	-1.284535	-3.404010	2.896188
H	-2.781461	-2.460374	3.164794
H	-2.657189	-3.565448	1.758066
C	-2.443307	2.618811	-2.226020
H	-1.694913	3.255305	-2.701336
H	-3.085865	2.170900	-2.997226
H	-3.057101	3.220431	-1.541682
C	-6.578332	0.432070	-0.644277
H	-7.608882	0.143198	-0.431224
H	-6.422514	1.481154	-0.359374
H	-6.375950	0.309772	-1.716860
C	-1.038873	-3.419788	-2.236112
H	-1.962431	-3.089363	-2.714660
H	-0.326177	-3.749996	-3.005074
H	-1.255441	-4.254006	-1.554778
C	3.529092	-0.385961	2.418082
H	3.577315	0.586447	2.911214
H	3.508298	-1.182500	3.174477
H	4.408802	-0.517899	1.773433
C	2.917803	-5.909331	-0.644965
H	3.682843	-6.657453	-0.430700
H	1.930621	-6.299738	-0.363716
H	2.925752	-5.671341	-1.717189
C	3.481397	0.804054	-2.235059
H	3.655185	-0.161611	-2.713006
H	3.414055	1.586256	-3.004407
H	4.311784	1.031703	-1.552676
C	-1.429435	3.256458	2.412758
H	-2.297150	2.814278	2.905334
H	-0.728890	3.635166	3.169626
H	-1.752461	4.084954	1.767673
C	3.667106	5.476087	-0.646550
H	3.934353	6.512302	-0.432735

H	4.497101	4.814860	-0.363831
H	3.458023	5.363585	-1.718928

10. TPB. R = $OCOCH_3$

E_{elec} = -2772.273871 a.u.; **H =** -2771.580281 a.u

C	-0.012421	2.640684	0.257624
C	-0.632039	1.404695	0.551197
C	-1.949237	1.526254	1.058704
C	-2.622319	2.733060	1.178317
C	-1.970869	3.903098	0.796401
C	-0.647188	3.871501	0.367517
B	0.022557	0.018131	0.217091
C	-0.909807	-1.184308	-0.183956
C	-0.769255	-2.446161	0.420072
C	-1.681858	-3.482105	0.255691
C	-2.764207	-3.282045	-0.594359
C	-2.929236	-2.076325	-1.274385
C	-2.011336	-1.059230	-1.053825
C	1.579182	-0.187219	0.260887
C	2.358809	0.249269	1.352387
C	3.739523	0.101236	1.408024
C	4.380554	-0.559251	0.365395
C	3.665189	-1.066597	-0.715717
C	2.293799	-0.839841	-0.764810
H	-3.642140	2.762560	1.545568
H	-0.121102	4.785251	0.114433
H	-1.577433	-4.413381	0.800539
H	-3.783111	-1.931615	-1.926224
H	4.313854	0.491016	2.240259
H	4.180886	-1.621777	-1.489436
O	-2.222956	0.194890	-1.626652
C	-2.302562	0.426716	-2.994308
O	-2.580970	1.550143	-3.336216
C	-1.989580	-0.715956	-3.925930
H	-1.187130	-1.353862	-3.548043
H	-2.879590	-1.338540	-4.063202
H	-1.714987	-0.292160	-4.892112
O	-3.662477	-4.317449	-0.795635
O	0.267927	-2.607804	1.346155
C	-4.881438	-4.367889	-0.092139
C	1.349144	-3.445735	1.094215
O	2.217244	-3.484084	1.929815
C	1.349415	-4.229633	-0.193727
O	-5.609434	-5.292231	-0.327258
C	-5.138432	-3.248390	0.882230
H	-6.046376	-3.481892	1.437186
H	-4.298484	-3.097111	1.565938
H	-5.271981	-2.304142	0.343689
H	0.689160	-5.097783	-0.097113
H	0.994157	-3.630877	-1.035333
H	2.366681	-4.579359	-0.369555
O	1.534305	-1.327658	-1.828508
O	1.698636	0.919892	2.371040
O	5.748261	-0.770988	0.435745
C	1.876483	-1.249062	-3.175614

C	1.725653	0.508848	3.709744
C	6.647234	0.126476	-0.167671
O	1.191188	-1.886716	-3.935978
C	3.010560	-0.349491	-3.600301
H	3.187259	0.478246	-2.911366
H	3.928167	-0.940743	-3.687287
H	2.765171	0.025424	-4.595486
O	1.201890	1.246578	4.504305
C	2.345311	-0.824931	4.028773
H	2.112061	-1.584848	3.278142
H	3.435051	-0.734234	4.079426
H	1.979563	-1.128207	5.010097
O	7.817194	-0.124209	-0.079469
C	6.036537	1.313253	-0.868003
H	5.357910	1.864025	-0.210624
H	6.842517	1.960556	-1.211346
H	5.436174	0.985908	-1.722460
O	-2.655698	0.375035	1.399579
O	1.329342	2.691105	-0.133451
O	-2.634863	5.112989	0.901372
C	-2.649475	-0.135281	2.698305
C	1.722820	2.452930	-1.432085
C	-3.334916	5.641994	-0.199180
O	-3.912418	6.680300	-0.036963
C	-3.271510	4.844449	-1.478213
O	-3.171650	-1.207072	2.868420
C	-2.019536	0.725884	3.757749
O	2.906853	2.473495	-1.672868
C	0.637376	2.160066	-2.435919
H	0.281274	1.135240	-2.282829
H	-0.231172	2.811272	-2.318073
H	1.052391	2.248854	-3.439186
H	-1.032807	1.088163	3.460184
H	-2.647001	1.608090	3.927703
H	-1.945821	0.150316	4.679299
H	-3.566065	3.802345	-1.323956
H	-3.925463	5.317446	-2.209589
H	-2.245578	4.829649	-1.860714

11. TPB. R = OH

E_{elec} = -1397.602005 a.u.; **H =** -1397.268809 a.u

C	-3.057009	-3.185023	0.009654
C	-1.928932	-3.308346	0.815958
C	-0.967254	-2.296221	0.787815
C	-1.083248	-1.132243	-0.002531
C	-2.250896	-1.062216	-0.787846
C	-3.226268	-2.064722	-0.804169
B	0.000261	-0.000593	-0.003125
C	-0.438441	1.503536	-0.002006
C	-1.501901	1.985091	0.791584
C	-1.898521	3.323737	0.819590
C	-1.231296	4.238560	0.009763
C	-0.178965	3.824844	-0.807242
C	0.202713	2.479211	-0.790495
C	1.522210	-0.372368	-0.002002

C	2.471184	0.310071	0.789593
C	3.828914	-0.015221	0.817813
C	4.287576	-1.052254	0.010321
C	3.403052	-1.758938	-0.804724
C	2.046769	-1.417064	-0.788419
O	2.002488	1.315291	1.600413
O	1.170098	-2.089921	-1.605898
H	4.528048	0.515277	1.459208
H	3.764649	-2.557477	-1.452415
O	5.632120	-1.337069	0.055234
O	-2.136439	1.077317	1.604613
O	1.222030	2.055164	-1.609564
H	-2.705902	3.664794	1.462570
H	0.330639	4.536521	-1.456658
O	-1.656417	5.545580	0.054486
O	0.138290	-2.394928	1.597512
O	-2.394764	0.034219	-1.604381
H	-1.819421	-4.179830	1.456370
H	-4.099097	-1.977044	-1.451010
O	-3.977003	-4.206073	0.054461
H	-2.838320	1.539881	2.089499
H	-1.099484	6.072712	-0.540530
H	1.591219	2.826500	-2.067826
H	-3.248928	-0.029952	-2.059962
H	0.089206	-3.235262	2.080368
H	-4.713460	-3.985368	-0.538001
H	1.653802	-2.796006	-2.062779
H	2.754189	1.693256	2.083954
H	5.810207	-2.084279	-0.538106

12. TPB. R = SO_3H

E_{elec} = -6336.398847 a.u.; **H =** -6335.946055 a.u

C	2.747928	-0.523970	1.082149
C	1.567995	-0.847777	0.354639
C	1.839618	-1.639659	-0.804664
C	3.126288	-1.812656	-1.319152
C	4.220581	-1.319321	-0.626653
C	4.045873	-0.748938	0.621511
B	0.115671	-0.143607	0.673728
C	-1.249076	-0.889025	0.089228
C	-2.032894	-0.457804	-1.003655
C	-3.307414	-0.948801	-1.302784
C	-3.824604	-1.978813	-0.537858
C	-3.081638	-2.511683	0.507105
C	-1.836181	-1.964012	0.812075
S	-1.493432	0.745393	-2.232173
S	-5.442240	-2.637178	-0.907820
S	-1.218250	-2.814340	2.304880
S	0.614703	-2.681111	-1.670033
S	5.853582	-1.487743	-1.320818
S	2.753586	0.107966	2.774178
C	-0.001984	1.509340	0.492894
C	0.603867	2.527363	-0.271284
C	-0.004138	3.768562	-0.447734
C	-1.231879	4.035955	0.163096

C	-1.737146	3.186283	1.136886
C	-1.070605	1.974913	1.284419
S	-2.180069	5.450922	-0.372560
S	2.315816	2.375311	-0.810416
S	-1.212293	0.925730	2.683233
H	3.288282	-2.344613	-2.247253
H	4.897694	-0.467910	1.229522
H	-3.879992	-0.544328	-2.130129
H	-3.479663	-3.336649	1.087685
H	0.448895	4.524455	-1.078205
H	-2.613316	3.453098	1.718518
O	0.349950	-2.717939	2.393377
O	-1.525413	-4.208551	2.168034
O	-1.839369	-2.089570	3.435698
H	0.694939	-1.922411	2.893575
O	-6.180900	-1.625427	-1.610557
O	-5.096464	-3.766071	-2.020505
O	-5.935395	-3.308542	0.271891
H	-4.979206	-4.620191	-1.561237
O	-1.941379	0.021724	-3.589678
O	-0.054220	0.856712	-2.243954
O	-2.329727	1.921443	-2.134190
H	-1.332659	-0.746473	-3.702498
O	4.119327	0.345690	3.164469
O	6.814081	-1.363988	-0.250438
O	1.554289	-3.686404	-2.517667
O	1.985815	1.506588	2.671877
O	5.837266	-2.589535	-2.244051
O	-0.066794	-3.480886	-0.680182
O	1.926801	-0.757810	3.593874
H	2.488602	2.087174	2.040403
O	5.955650	-0.164170	-2.251391
H	6.300782	0.567767	-1.704177
O	-0.110662	-1.977515	-2.701390
H	1.807216	-4.431588	-1.937351
O	-0.894086	1.593954	3.905869
O	-0.221590	-0.154036	2.240969
O	-1.345756	6.619608	-0.423080
O	-2.402251	5.051057	-1.919047
O	3.085411	2.681818	0.384928
O	2.615016	1.198966	-1.580010
O	-2.599484	0.233266	2.735435
H	-2.424716	-0.751940	3.075096
O	2.510144	3.661858	-1.758686
H	2.358119	3.375638	-2.681857
O	-3.433458	5.417968	0.340523
H	-2.877789	4.195385	-1.984512

13. H^-

E_{elec} = -0.505923 a.u.; **H =** -0.503562 a.u

H.	0.000000	0.000000	0.000000

14. TPB hydride. R = CF_3

E_{elec} = -3755.895195 a.u.; **H =** -3755.529546 a.u

C	-0.847513	-2.467308	0.923671

C	-0.974991	-1.318706	0.079159
C	-2.031638	-1.431881	-0.870661
C	-2.841787	-2.565733	-0.985496
C	-2.642013	-3.662853	-0.165045
C	-1.642597	-3.605112	0.796225
B	0.000322	-0.000539	0.393855
C	-0.653977	1.503245	0.080486
C	-0.225586	2.474822	-0.870291
C	-0.804031	3.742582	-0.985709
C	-1.853652	4.117592	-0.164540
C	-2.301062	3.223565	0.798144
C	-1.711809	1.966819	0.925950
C	0.960202	2.295711	-1.807705
F	0.908481	3.165595	-2.868915
C	-2.518165	5.451257	-0.328634
F	-3.604157	5.393701	-1.161239
C	-2.243328	1.173432	2.118820
F	-3.384326	1.741670	2.635686
C	-2.468690	-0.314819	-1.807767
F	-3.197361	-0.793955	-2.868456
C	-3.466911	-4.903785	-0.328859
F	-2.878353	-5.814502	-1.165594
C	0.106873	-2.532459	2.115300
F	0.184629	-3.805009	2.631530
C	1.629978	-0.185710	0.080564
C	2.257515	-1.042499	-0.870124
C	3.644657	-1.175167	-0.985191
C	4.494070	-0.453715	-0.163732
C	3.943289	0.380765	0.798723
C	2.560217	0.498788	0.926059
C	5.981245	-0.545435	-0.328209
F	6.473329	0.418176	-1.167997
C	1.510238	-1.980310	-1.807753
F	0.390092	-1.480204	-2.358947
C	2.138619	1.356528	2.118318
F	3.201254	2.060324	2.635411
F	-3.301292	0.570685	-1.176986
F	-1.474271	0.401408	-2.361719
F	1.388287	-2.196244	1.841855
F	-0.310404	-1.756539	3.149271
F	-4.697761	-4.649129	-0.855035
F	-3.668625	-5.555671	0.851591
F	1.084592	1.076150	-2.360821
F	2.143027	2.575775	-1.177090
F	-2.591784	-0.105026	1.846718
F	-1.361700	1.148724	3.151897
F	-1.685472	6.389986	-0.859680
F	-2.978264	5.953572	0.852552
F	2.289085	-2.367427	-2.870353
F	1.164781	-3.146332	-1.178262
F	1.206351	2.297933	1.845199
F	1.675525	0.606226	3.151604
F	6.377832	-1.739465	-0.851550
F	6.647228	-0.389892	0.851287
H	-3.635877	-2.589812	-1.720514

H	-1.497777	-4.445564	1.462027
H	-0.429230	4.442144	-1.721592
H	-3.100873	3.518010	1.464659
H	4.063355	-1.849483	-1.720995
H	4.597935	0.926271	1.465403
H	-0.000566	-0.001319	1.593894

15. TPB hydride. R = CH_3
E_{elec} = -1074.922920 a.u.; **H =** -1074.368678 a.u

C	-2.371999	-0.271100	0.803206
C	-1.572122	0.353792	-0.188937
C	-2.220659	1.352012	-0.970430
C	-3.566223	1.682569	-0.770038
C	-4.345380	1.048439	0.199266
C	-3.719979	0.075822	0.978218
B	-0.000628	0.000599	-0.542290
C	1.091628	1.184431	-0.189648
C	2.280511	1.245379	-0.971123
C	3.241005	2.243978	-0.770524
C	3.082758	3.235966	0.198869
C	1.927331	3.182576	0.977308
C	0.951408	2.190115	0.802052
C	2.560708	0.215661	-2.044078
C	4.108898	4.331132	0.381410
C	-0.232033	2.230843	1.747314
C	-1.469861	2.110825	-2.043024
C	-5.807534	1.387013	0.381266
C	-1.813837	-1.314778	1.749144
C	0.479362	-1.536957	-0.189155
C	-0.060477	-2.597638	-0.971105
C	0.325920	-3.928220	-0.770649
C	1.264101	-4.286024	0.199206
C	1.793402	-3.258184	0.978462
C	1.420086	-1.917284	0.803311
C	1.701533	-5.721653	0.381658
C	-1.092582	-2.326714	-2.044139
C	2.044495	-0.912265	1.749719
H	-4.016089	2.462547	-1.387675
H	-4.293413	-0.423667	1.761791
H	4.141553	2.242274	-1.387968
H	1.782327	3.929273	1.760709
H	-0.124405	-4.707759	-1.388510
H	2.512160	-3.505187	1.762481
H	3.553000	0.374160	-2.484537
H	2.510742	-0.802907	-1.643581
H	1.806763	0.265645	-2.839301
H	4.083508	4.733908	1.401048
H	5.124112	3.964437	0.185826
H	3.929157	5.170736	-0.305911
H	-0.027957	2.910753	2.584067
H	-1.146410	2.567378	1.246208
H	-0.456782	1.240822	2.155242
H	-2.104343	2.889902	-2.483723
H	-0.563805	2.578337	-1.641896

H	-1.134494	1.433570	-2.838113
H	-2.504877	-1.479352	2.585496
H	-1.645486	-2.274649	1.248427
H	-0.845130	-1.011845	2.157652
H	-6.143485	1.163708	1.400978
H	-5.999458	2.449079	0.184905
H	-6.443575	0.809930	-0.305816
H	2.528744	-1.428564	2.588199
H	2.794827	-0.289060	1.250582
H	1.298349	-0.222370	2.155019
H	-1.450238	-3.265748	-2.484644
H	-1.950432	-1.775343	-1.643517
H	-0.673214	-1.698074	-2.839308
H	2.064686	-5.900164	1.400864
H	0.876993	-6.418668	0.187493
H	2.517869	-5.985000	-0.306735
H	-0.000821	0.000492	-1.769659

16. TPB hydride. R = CN
E_{elec} = -1551.591233 a.u.; **H =** -1551.293166 a.u

C	-2.438871	0.879847	-0.944222
C	-1.581361	0.003222	-0.227563
C	-2.231265	-0.822048	0.721744
C	-3.616416	-0.790077	0.934062
C	-4.418488	0.088089	0.199313
C	-3.820484	0.933854	-0.743403
B	-0.000134	0.000119	-0.610547
C	0.787800	-1.370860	-0.227604
C	1.827122	-1.521200	0.722060
C	2.491989	-2.736763	0.934398
C	2.132734	-3.870356	0.199372
C	1.101531	-3.775240	-0.743608
C	0.457573	-2.551698	-0.944492
C	2.243372	-0.436126	1.562100
N	2.628790	0.392012	2.281856
C	2.807127	-5.112264	0.409665
N	3.356843	-6.123387	0.577754
C	-0.603050	-2.529760	-1.910054
N	-1.474149	-2.572876	-2.678994
C	-1.499837	-1.725610	1.561342
N	-0.975614	-2.473974	2.280750
C	-5.831210	0.124914	0.409594
N	-6.981730	0.154320	0.577678
C	-1.889473	1.787771	-1.909383
N	-1.491162	2.564008	-2.677983
C	0.793383	1.367896	-0.227787
C	0.403580	2.343778	0.721072
C	1.124131	3.527210	0.933326
C	2.286090	3.782119	0.199021
C	2.719632	2.840912	-0.743221
C	1.981721	1.671610	-0.944037
C	3.024696	4.986950	0.409268
N	3.625725	5.968445	0.577319
C	-0.744914	2.162684	1.560411

N	-1.655353	2.083246	2.279576
C	2.493318	0.741408	-1.908763
N	2.966329	0.007924	-2.677011
H	-4.063705	-1.440739	1.677774
H	-4.427366	1.625926	-1.317276
H	3.278867	-2.798903	1.678372
H	0.805783	-4.646785	-1.317666
H	0.784222	4.240221	1.676706
H	3.622730	3.019975	-1.316765
H	-0.000195	0.000110	-1.830696

17. TPB hydride. R = 5F

E_{elec} = -2210.192388 a.u.; **H =** -2210.004197 a.u

C	0.352125	-2.248671	0.676372
C	-0.282505	-1.550640	-0.351164
C	-1.226290	-2.301258	-1.057281
C	-1.519223	-3.636344	-0.790435
C	-0.846224	-4.287918	0.238477
C	0.098319	-3.586062	0.978956
B	0.003731	0.002334	-0.799453
C	1.491059	0.532832	-0.352315
C	2.612143	0.112667	-1.072922
C	3.913208	0.531483	-0.805523
C	4.140103	1.422312	0.238947
C	3.060799	1.867117	0.994236
C	1.777499	1.414182	0.690290
F	2.467287	-0.767362	-2.096749
F	4.960046	0.088397	-1.543139
F	5.394105	1.846453	0.517930
F	3.275002	2.725261	2.020512
F	0.786412	1.872433	1.499823
F	-1.929514	-1.725906	-2.066413
F	-2.445182	-4.311239	-1.513876
F	-1.110441	-5.585246	0.516693
F	0.749146	-4.210170	1.990117
F	1.264930	-1.630302	1.471412
C	-1.199725	1.023220	-0.348829
C	-2.090400	0.834034	0.708041
C	-3.127238	1.713429	1.017055
C	-3.300873	2.861656	0.252239
C	-2.431867	3.107246	-0.806188
C	-1.414658	2.195369	-1.078212
F	-1.970684	-0.244264	1.526726
F	-0.597102	2.506268	-2.116722
F	-2.590523	4.226740	-1.553122
F	-4.298347	3.730484	0.535837
F	-3.961638	1.473896	2.057208
H	0.002346	0.001397	-2.009348

18. TPB hydride. R = F

E_{elec} = -1614.520717 a.u.; **H =** -1614.291417 a.u

C	1.177791	1.985802	0.722719
C	1.201030	1.033951	-0.298948
C	2.415362	1.042144	-0.993445
C	3.499078	1.885863	-0.754059

C	3.356060	2.801288	0.276339
C	2.203355	2.877869	1.039426
B	-0.001268	0.010322	-0.742335
C	0.287000	-1.540914	-0.292417
C	-0.268662	-2.606201	-1.008118
C	-0.067200	-3.963333	-0.758359
C	0.760726	-4.281743	0.305975
C	1.356788	-3.310698	1.092972
C	1.087896	-1.981731	0.763570
F	-1.101556	-2.324392	-2.053654
F	0.992844	-5.602784	0.592911
F	1.669734	-1.056791	1.584387
F	2.591495	0.140335	-2.004492
F	4.392761	3.655896	0.552401
F	0.071658	2.076710	1.520724
C	-1.491276	0.528744	-0.293914
C	-2.169127	1.498257	-1.039845
C	-3.452424	1.983877	-0.790007
C	-4.113048	1.455672	0.307277
C	-3.535763	0.499581	1.126028
C	-2.246460	0.083009	0.793246
F	-5.378579	1.899051	0.595573
F	-1.538355	2.051710	-2.117713
F	-1.700647	-0.835872	1.644889
H	4.405406	1.823022	-1.344703
H	2.099952	3.587273	1.852035
H	-0.538127	-4.725938	-1.367391
H	1.993363	-3.564300	1.932439
H	-3.904280	2.738051	-1.423490
H	-4.050910	0.098876	1.991073
H	-0.000027	0.007997	-1.954158

19. TPB hydride. R = H

E_{elec} = -720.883663 a.u.; **H =** -720.589478 a.u

C	-4.241537	0.572500	-0.466048
C	-3.935492	-0.258040	0.617023
C	-2.611609	-0.406983	1.033372
C	-1.537035	0.252590	0.401607
C	-1.881852	1.080761	-0.683644
C	-3.202954	1.242575	-1.114918
B	-0.000308	0.000310	0.903250
C	0.987031	1.204661	0.401902
C	0.954000	2.465080	1.033653
C	1.745861	3.536504	0.617399
C	2.618199	3.385622	-0.465560
C	2.678363	2.151098	-1.114363
C	1.876751	1.088559	-0.683230
C	0.549291	-1.456796	0.401828
C	1.652500	-2.062116	1.038490
C	2.185203	-3.283049	0.621499
C	1.624143	-3.958562	-0.467241
C	0.529773	-3.389645	-1.121007
C	0.009472	-2.164911	-0.688931
H	2.102781	-1.549117	1.888257
H	-0.848538	-1.742908	-1.210778

H	3.038537	-3.713883	1.145501
H	0.078694	-3.902676	-1.970447
H	2.032424	-4.912725	-0.796851
H	0.279354	2.600776	1.878929
H	1.944758	0.132749	-1.201288
H	1.687737	4.492771	1.137543
H	3.353520	2.014763	-1.959227
H	3.241218	4.215969	-0.794396
H	-2.391345	-1.058834	1.878722
H	-1.088385	1.618076	-1.201593
H	-4.734203	-0.787099	1.137150
H	-3.422930	1.895085	-1.959942
H	-5.272187	0.695977	-0.795102
H	-0.000539	0.000428	2.136290

20. TPB hydride. R = NH_2

E_{elec} = -1219.390041 a.u.; **H =** -1218.935073 a.u

C	-2.427045	0.015516	-0.769052
C	-1.524937	-0.542228	0.174511
C	-2.034426	-1.643535	0.917251
C	-3.342883	-2.136139	0.753836
C	-4.203401	-1.531901	-0.163380
C	-3.747577	-0.456755	-0.923679
B	-0.000198	-0.000523	0.534685
C	1.231955	-1.049504	0.174580
C	2.440532	-0.938087	0.916906
C	3.522192	-1.824040	0.754108
C	3.429999	-2.872513	-0.161898
C	2.270844	-3.017469	-0.921553
C	1.200674	-2.110901	-0.767544
N	2.558152	0.069324	1.883390
N	4.488480	-3.819961	-0.277475
N	0.123920	-2.227389	-1.656086
N	-1.222041	-2.249497	1.884636
N	-5.553511	-1.973576	-0.279791
N	-1.987552	1.004306	-1.658647
C	0.292769	1.591001	0.175218
C	-0.405938	2.582346	0.918845
C	-0.178864	3.961935	0.755716
C	0.773752	4.405721	-0.162170
C	1.476789	3.473893	-0.923199
C	1.226093	2.094000	-0.768783
N	1.065753	5.795912	-0.278254
N	-1.335973	2.180897	1.886740
N	1.862490	1.219351	-1.658845
H	-3.689703	-2.972323	1.362344
H	-4.400416	0.001694	-1.667735
H	4.419878	-1.704666	1.362131
H	2.200697	-3.813113	-1.664532
H	-0.729387	4.680018	1.364854
H	2.199591	3.810537	-1.667664
H	-2.712117	1.646468	-1.959987
H	-1.151985	1.490963	-1.333543
H	-1.404230	-3.236912	2.022190
H	-0.231959	-2.050188	1.760363

H	-5.919936	-1.814015	-1.214985
H	-5.644030	-2.961361	-0.055577
H	-2.100023	2.832080	2.025549
H	-1.658170	1.223789	1.762161
H	2.780654	1.526065	-1.960631
H	1.866658	0.252325	-1.333945
H	1.386825	6.033884	-1.213454
H	0.255458	6.367860	-0.053583
H	3.504189	0.405517	2.021501
H	1.890438	0.826879	1.758051
H	-0.069468	-3.176827	-1.955032
H	-0.715497	-1.747223	-1.331194
H	4.533648	-4.218195	-1.212188
H	5.388829	-3.403286	-0.054010
H	-0.000158	-0.000957	1.767545

21. TPB hydride. R = NO_2

E_{elec} = -2562.572236 a.u.; **H =** -2562.236064 a.u

C	0.031138	2.438208	1.008136
C	0.412428	1.391118	0.139643
C	1.294934	1.842370	-0.867827
C	1.707989	3.160887	-1.037306
C	1.242335	4.113270	-0.149362
C	0.418375	3.764062	0.911648
B	-0.074576	-0.147625	0.505497
C	1.096445	-1.256279	0.212258
C	1.028243	-2.492634	-0.469026
C	2.066610	-3.406115	-0.589134
C	3.300891	-3.075392	-0.050619
C	3.479959	-1.880302	0.631438
C	2.379274	-1.048737	0.770272
N	-0.214753	-2.941347	-1.155297
O	-0.875481	-2.106002	-1.768281
N	4.434256	-4.001748	-0.200720
O	4.242829	-5.047537	-0.830408
N	2.679665	0.161273	1.599690
O	2.105751	0.281598	2.677320
N	1.891061	0.909902	-1.874519
O	1.288168	-0.126669	-2.137349
N	1.647414	5.523890	-0.317608
O	2.380623	5.800408	-1.270898
N	-0.876397	2.178121	2.164511
O	-0.475606	2.538813	3.271066
C	-1.634058	-0.441078	0.088184
C	-2.402887	0.269654	-0.856957
C	-3.758198	0.112487	-1.090312
C	-4.448231	-0.817117	-0.322091
C	-3.805109	-1.524624	0.680919
C	-2.443075	-1.318038	0.848167
N	-5.885637	-1.025974	-0.554678
O	-6.428071	-0.350413	-1.437289
N	-1.797212	1.327295	-1.721394
O	-1.191195	0.970145	-2.725338
N	-1.860954	-2.100117	1.971214
O	-0.787298	-2.672400	1.783173

O	2.959215	1.251888	-2.393570
O	-1.976079	1.681203	1.928430
O	1.224778	6.339800	0.507968
O	-0.482419	-4.146378	-1.094935
O	3.536958	0.931295	1.154236
O	5.510946	-3.678086	0.314324
O	-2.007876	2.497300	-1.387208
O	-2.515040	-2.149942	3.014989
O	-6.465866	-1.863004	0.145298
H	2.382681	3.429356	-1.838817
H	0.093490	4.502527	1.633636
H	1.919764	-4.346871	-1.103149
H	4.444182	-1.617203	1.048734
H	-4.276172	0.695824	-1.842112
H	-4.350376	-2.207228	1.319780
H	-0.119880	-0.143947	1.715635

22. TPB hydride. R = OCH_3

E_{elec} = -1752.161379 a.u.; **H =** -1751.558243 a.u

C	-2.347230	0.328090	-0.588609
C	-1.554573	-0.348055	0.346308
C	-2.225912	-1.399180	1.005163
C	-3.569901	-1.736119	0.793493
C	-4.304759	-0.994465	-0.129778
C	-3.707875	0.044829	-0.835588
B	0.000050	0.002579	0.753562
C	1.078424	-1.171044	0.344382
C	2.323069	-1.235238	1.004872
C	3.282220	-2.235099	0.791576
C	3.003820	-3.237387	-0.135774
C	1.807288	-3.230942	-0.844923
C	0.886448	-2.190457	-0.596143
O	2.561818	-0.207100	1.897639
C	3.811115	-0.170707	2.558709
O	3.995005	-4.207319	-0.291423
C	3.734794	-5.234088	-1.234431
O	-0.278948	-2.115191	-1.339141
C	-0.695924	-3.248429	-2.073063
O	-1.449854	-2.116724	1.895769
C	-2.035877	-3.221701	2.554993
O	-5.638307	-1.375146	-0.284386
C	-6.402605	-0.639515	-1.225627
O	-1.706801	1.306896	-1.329092
C	-2.488312	2.248434	-2.036047
C	0.475672	1.522742	0.341888
C	-0.103770	2.632714	0.992192
C	0.279923	3.963946	0.780674
C	1.297949	4.226026	-0.134629
C	1.903216	3.187051	-0.833392
C	1.464453	1.868102	-0.586947
O	1.638878	5.570420	-0.288649
C	2.667149	5.860926	-1.221188
O	-1.121788	2.322891	1.874117
C	-1.797349	3.385570	2.517360
O	1.995921	0.822782	-1.322890

C	3.217532	1.020498	-2.005649
H	-4.064843	-2.544635	1.319438
H	-4.261680	0.609140	-1.575207
H	4.228782	-2.266446	1.319056
H	1.594189	-3.989219	-1.587719
H	-0.175676	4.798663	1.301186
H	2.673924	3.381466	-1.568473
H	3.786374	0.725881	3.183592
H	3.967281	-1.055261	3.196734
H	4.649118	-0.095989	1.847408
H	-1.701459	-3.010801	-2.430127
H	-0.042556	-3.449676	-2.938801
H	-0.736772	-4.149734	-1.441925
H	4.608088	-5.892060	-1.210572
H	2.835299	-5.810672	-0.969938
H	3.609335	-4.830884	-2.251092
H	3.526022	0.028067	-2.344948
H	3.105838	1.683205	-2.880503
H	3.991236	1.435912	-1.341765
H	-2.568207	2.913767	3.132244
H	-1.124775	3.972540	3.163119
H	-2.273942	4.065380	1.793204
H	2.795714	6.946766	-1.197831
H	3.615322	5.374537	-0.945742
H	2.392784	5.547718	-2.240281
H	-1.784611	3.008467	-2.385802
H	-2.998151	1.800554	-2.905805
H	-3.242155	2.720218	-1.386889
H	-1.244037	-3.645460	3.178180
H	-2.881168	-2.920533	3.194284
H	-2.386607	-3.985100	1.842307
H	-7.406512	-1.072761	-1.201490
H	-6.458221	0.427082	-0.959617
H	-5.990976	-0.728651	-2.242751
H	0.001491	0.004536	1.974228

23. TPB hydride. R = $OCOCH_3$

E_{elec} = -2772.988113 a.u.; **H =** -2772.285146 a.u

C	2.278654	-0.821623	-0.613907
C	1.610548	-0.188532	0.446617
C	2.484122	0.273927	1.449351
C	3.869871	0.149492	1.420045
C	4.445887	-0.529599	0.355728
C	3.656374	-1.053589	-0.656017
B	-0.004684	-0.068413	0.686649
C	-0.923024	-1.195359	-0.085284
C	-0.832815	-2.534290	0.338711
C	-1.746957	-3.536223	0.024892
C	-2.815922	-3.213396	-0.799615
C	-2.931534	-1.939111	-1.332024
C	-1.977783	-0.981177	-0.985068
O	0.189720	-2.857798	1.247980
C	1.198345	-3.741865	0.938263
C	1.228304	-4.339194	-0.450881
O	-3.778871	-4.186485	-1.105713

C	-4.896545	-4.317417	-0.291012
C	-4.996888	-3.377595	0.885645
O	-2.185243	0.323114	-1.476189
C	-2.238770	0.604212	-2.815428
C	-1.791843	-0.461230	-3.790790
O	1.949514	0.989215	2.529786
C	1.691211	0.354934	3.731648
C	1.959586	-1.125236	3.794748
O	5.832089	-0.742052	0.333450
C	6.659519	0.158102	-0.320758
C	5.992000	1.332506	-0.993186
O	1.485175	-1.350707	-1.649577
C	1.830013	-1.339329	-2.978207
C	2.880150	-0.369200	-3.475413
C	-0.631673	1.445508	0.668390
C	0.001502	2.651824	0.338737
C	-0.602143	3.910571	0.383441
C	-1.933696	3.993923	0.757856
C	-2.625014	2.853512	1.147241
C	-1.958544	1.634867	1.110088
O	1.368122	2.685458	-0.027293
C	1.749807	2.429760	-1.310054
C	0.662852	2.044379	-2.279538
O	-2.728883	0.507059	1.440871
C	-2.701096	-0.027356	2.712828
C	-1.994949	0.779797	3.770900
O	-2.583733	5.237574	0.785949
C	-3.193072	5.716684	-0.362772
C	-3.067893	4.872376	-1.609321
O	-2.604034	1.712986	-3.145735
O	-5.703497	-5.167359	-0.581326
O	2.006563	-3.994826	1.802334
O	1.218341	-2.083796	-3.714207
O	1.260998	1.029184	4.638911
O	7.846631	-0.061975	-0.313836
O	-3.290217	-1.067381	2.898426
O	2.929681	2.505720	-1.588232
O	-3.768464	6.776279	-0.294968
H	-3.663824	2.905976	1.455082
H	-0.038377	4.797138	0.112992
H	-1.656008	-4.530103	0.449714
H	-3.775398	-1.682806	-1.963756
H	4.480240	0.562130	2.216186
H	4.111694	-1.630217	-1.452742
H	-1.035262	-1.128097	-3.373977
H	-2.649937	-1.074541	-4.084508
H	-1.413460	0.043907	-4.680945
H	-5.917775	-3.600875	1.423596
H	-4.135523	-3.477479	1.552243
H	-4.993526	-2.333752	0.558158
H	0.589890	-5.228957	-0.475283
H	0.871862	-3.645774	-1.213519
H	2.255727	-4.644620	-0.653832
H	3.074681	0.458005	-2.791626
H	3.814226	-0.911822	-3.653905

H	2.530558	0.005399	-4.440281
H	1.392006	-1.646458	3.018355
H	3.016893	-1.342644	3.615915
H	1.667648	-1.486015	4.780770
H	5.340064	1.879073	-0.306546
H	6.767995	1.987045	-1.389236
H	5.343678	0.990482	-1.805490
H	0.293664	1.051711	-2.002282
H	-0.193490	2.720154	-2.225924
H	1.072522	2.025595	-3.288634
H	-1.011655	1.113707	3.435621
H	-2.586979	1.675528	3.993520
H	-1.897381	0.173098	4.670245
H	-3.360147	3.832530	-1.440150
H	-3.684421	5.319734	-2.388475
H	-2.024003	4.845761	-1.938680
H	-0.145394	-0.404753	1.853963

24. TPB hydride. R = OH

E_{elec} = -1398.230880 a.u.; **H =** -1397.891361 a.u

C	0.909744	-2.127162	0.742336
C	0.111888	-1.580659	-0.272206
C	-0.621450	-2.545410	-0.985205
C	-0.564114	-3.930591	-0.753273
C	0.266970	-4.402127	0.253416
C	1.010491	-3.505316	1.013324
B	0.000034	0.004706	-0.693439
C	-1.429008	0.697721	-0.270649
C	-1.909704	1.805210	-0.991130
C	-3.141615	2.440954	-0.758583
C	-3.956661	1.960137	0.256754
C	-3.539302	0.878424	1.025098
C	-2.292306	0.283627	0.753100
O	-1.096935	2.308520	-1.993078
O	-5.180368	2.595687	0.475446
O	-1.881820	-0.759671	1.566356
O	-1.469722	-2.086915	-1.979227
O	0.320084	-5.780033	0.471962
O	1.624291	-1.255640	1.547601
C	1.317980	0.892681	-0.273956
C	2.520806	0.733339	-0.984465
C	3.694035	1.471907	-0.753057
C	3.688650	2.431015	0.250207
C	2.539471	2.633747	1.007090
C	1.393795	1.860906	0.736690
O	4.857887	3.162106	0.468443
O	2.545847	-0.234570	-1.974501
O	0.280769	2.051682	1.538896
H	-1.151827	-4.631859	-1.343940
H	1.652988	-3.864835	1.821123
H	-3.464270	3.293183	-1.354978
H	-4.164545	0.504791	1.839985
H	4.595701	1.307956	-1.341457
H	2.530695	3.373161	1.811922
H	-1.556409	3.072555	-2.375739

H	-5.613333	2.126030	1.205362
H	-2.607711	-0.955533	2.178785
H	0.479991	2.781477	2.145584
H	3.442055	-0.239221	-2.346084
H	4.669357	3.783246	1.189176
H	2.157068	-1.790790	2.155982
H	-1.917304	-2.862725	-2.352169
H	0.950142	-5.924787	1.195070
H	-0.001053	0.004075	-1.909557

25. TPB hydride. R = SO_3H

E_{elec} = -6337.105317 a.u.; **H =** -6336.642779 a.u

C	-1.007709	1.923141	1.526069
C	0.092092	1.522943	0.755623
C	0.735080	2.595841	0.099583
C	0.090360	3.813993	-0.093198
C	-1.192725	4.000608	0.430095
C	-1.717025	3.106926	1.358547
B	0.151847	-0.149372	0.781705
O	-0.189842	-0.307289	2.315290
S	-1.203363	0.754977	2.852192
O	-2.540466	0.142021	2.904846
S	2.500122	2.525055	-0.275327
O	2.852205	4.103189	-0.395074
S	-2.183786	5.330398	-0.213403
O	-3.508952	5.198965	0.346847
C	1.568966	-0.762196	0.209491
C	2.789774	-1.006954	1.105022
C	4.032524	-1.021617	0.467480
C	4.063097	-1.231848	-0.908580
C	2.938252	-1.823750	-1.550749
C	1.712234	-1.806143	-0.941035
S	2.887982	-0.825945	2.778322
O	1.881561	-1.707437	3.561624
S	0.494778	-2.798455	-1.721836
O	-0.199087	-2.106370	-2.805032
S	5.526720	-0.957269	-1.815203
O	5.079156	0.285536	-2.773297
C	-1.241860	-0.767582	0.091767
C	-1.941000	-0.216979	-1.002951
C	-3.243543	-0.562760	-1.374224
C	-3.888872	-1.565584	-0.671658
C	-3.225503	-2.238518	0.348256
C	-1.939961	-1.844565	0.706238
S	-1.185532	0.892858	-2.192597
O	-1.953795	2.123814	-2.270158
S	-5.526239	-2.071309	-1.143386
O	-6.114882	-2.804202	-0.045229
S	-1.298649	-2.972261	1.989872
O	-1.980960	-2.421347	3.311393
O	-1.498059	0.121458	-3.549056
O	0.244609	0.929465	-2.009002
O	-6.191623	-0.974682	-1.793953
O	-5.224113	-3.127479	-2.345670
O	0.157528	-2.916562	2.100762

O	-1.862274	-4.277797	1.758738
O	1.365869	-3.887911	-2.563834
O	-0.310078	-3.530989	-0.769782
O	6.560877	-0.504483	-0.906486
O	5.795079	-2.021664	-2.757358
O	4.208842	-1.007270	3.318095
O	2.326721	0.618027	3.162152
O	-1.476502	6.586109	-0.162819
O	-2.182620	4.930396	-1.772372
O	3.135797	2.092014	0.954197
O	2.889162	1.932952	-1.539507
O	-0.731957	1.370953	4.067319
H	3.110673	-2.352994	-2.481925
H	4.959842	-0.899065	1.016671
H	-3.731757	-0.072429	-2.209545
H	-3.704528	-3.066844	0.857795
H	0.549129	4.611968	-0.663495
H	-2.647793	3.313562	1.875923
H	1.127135	-2.093207	2.979656
H	-4.972755	-3.980324	-1.941561
H	-0.982643	-0.737151	-3.480658
H	2.629245	1.246326	2.453222
H	4.537888	0.906872	-2.239179
H	1.764516	-4.499127	-1.914706
H	-2.221759	-1.442343	3.217714
H	3.059486	4.278879	-1.333033
H	-2.317225	3.957121	-1.893664
H	1.886154	0.121791	-0.365582

26. TPB NMR. R = CF_3

E_{elec} = -4409.671311 a.u.; **H =** -4409.098942 a.u

C	1.510854	1.698715	1.369993
C	0.878686	0.435475	1.132531
C	1.700802	-0.642323	1.606038
C	3.057751	-0.519701	1.905653
C	3.685430	0.712695	1.829133
C	2.879615	1.822014	1.657990
B	-0.357783	0.160393	-0.072065
C	0.762447	-0.641969	-1.120523
C	1.807061	0.133492	-1.742901
C	3.095425	-0.359610	-1.953240
C	3.397793	-1.687736	-1.697179
C	2.347403	-2.536812	-1.411747
C	1.061445	-2.043188	-1.147985
C	1.641117	1.534667	-2.318540
F	0.485845	1.675591	-3.034870
C	4.810297	-2.197499	-1.771625
F	4.869821	-3.480659	-2.212864
C	0.094911	-3.229369	-1.093876
F	0.613718	-4.304178	-0.440165
C	1.215795	-2.027990	1.993356
F	-0.041719	-2.322722	1.612292
C	5.175867	0.851427	1.968805
F	5.727232	-0.179436	2.654623
C	0.887582	3.074387	1.575334

F	1.549871	4.058328	0.885268
C	-1.858717	-0.510337	0.286381
C	-2.436768	-0.534135	1.598073
C	-3.673404	-1.120010	1.879918
C	-4.459514	-1.649612	0.869002
C	-4.003185	-1.559730	-0.430552
C	-2.745461	-1.012462	-0.717227
C	-1.834656	0.156862	2.822286
F	-0.775003	-0.462897	3.378682
C	-2.519786	-1.128369	-2.225782
F	-2.556318	-2.412003	-2.653096
C	-5.814378	-2.228894	1.180627
F	-6.225407	-3.108670	0.234667
F	-3.566540	-0.524386	-2.896498
F	-1.391069	-0.576059	-2.709470
F	-6.766633	-1.251231	1.249903
F	-5.828976	-2.871876	2.374537
F	-1.455591	1.426309	2.531744
F	-2.751376	0.282230	3.827574
F	1.687903	2.547605	-1.412961
F	2.628334	1.823363	-3.213264
F	-1.119660	-3.026346	-0.557853
F	-0.107948	-3.650010	-2.382695
F	5.406679	-2.177953	-0.544423
F	5.586118	-1.447557	-2.595559
F	1.258970	-2.155062	3.352626
F	2.026758	-3.018313	1.523478
F	5.525687	2.000031	2.607016
F	5.783597	0.885555	0.747147
F	-0.420934	3.258141	1.297182
F	1.019073	3.406543	2.894438
P	-1.759594	2.607606	-1.102881
C	-3.079000	2.741118	0.153883
C	-4.401499	3.401654	-0.268576
C	-2.590846	2.240045	-2.680044
C	-3.098450	3.449334	-3.487532
C	-0.869619	4.178369	-1.375295
C	-1.629656	5.459676	-0.997597
H	2.522194	-3.604758	-1.367658
H	3.867700	0.294493	-2.335960
H	3.620000	-1.399451	2.197449
H	3.309972	2.811604	1.762954
H	-4.611432	-1.945770	-1.240975
H	-4.036198	-1.146261	2.898965
H	-1.878392	1.666578	-3.275274
H	-3.421954	1.572569	-2.449562
H	0.058836	4.117267	-0.815286
H	-0.591054	4.180623	-2.434134
H	-3.263924	1.698998	0.436713
H	-2.661304	3.227874	1.036500
H	-0.976630	6.320789	-1.167684
H	-2.532405	5.604343	-1.596355
H	-1.907530	5.458225	0.060257
H	-5.099022	3.353449	0.573146
H	-4.275811	4.453610	-0.534777

H	-4.867081	2.881760	-1.110279
H	-3.599751	3.078107	-4.386457
H	-3.819599	4.053796	-2.932676
H	-2.280343	4.098237	-3.810656
O	-0.725923	1.524713	-0.699313

27. TPB NMR. R = CH_3

E_{elec} = -1728.788448 a.u.; **H =** -1728.025176 a.u

C	0.227396	2.604952	-0.473095
C	0.675205	1.411771	-1.099972
C	1.754797	1.555332	-2.015158
C	2.407890	2.783186	-2.169889
C	2.033937	3.924796	-1.459754
C	0.915207	3.815068	-0.637621
B	0.010412	-0.041913	-0.723909
O	-0.013990	0.050672	0.972378
P	-0.037058	0.139593	2.475630
C	1.095424	1.462564	3.036704
C	0.795840	2.134677	4.385069
C	2.236553	0.433624	-2.915977
C	2.793663	5.222296	-1.596801
C	-1.061540	2.703133	0.326337
C	0.959302	-1.363849	-0.941580
C	0.608355	-2.433580	-1.811200
C	1.354123	-3.617413	-1.832041
C	2.477904	-3.808458	-1.027057
C	2.883751	-2.728083	-0.247568
C	2.169441	-1.522613	-0.215390
C	-0.538564	-2.358697	-2.801441
C	2.839879	-0.399332	0.558704
C	3.229993	-5.117599	-1.021510
C	-1.590055	-0.224274	-1.041912
C	-2.277207	0.547489	-2.019639
C	-3.670524	0.488260	-2.133324
C	-4.453838	-0.330583	-1.318801
C	-3.778782	-1.161893	-0.428461
C	-2.383010	-1.140698	-0.301486
C	-1.569666	1.431838	-3.029152
C	-5.960392	-0.330587	-1.417842
C	-1.803079	-2.224236	0.592900
C	0.498207	-1.454374	3.197129
C	1.131391	-1.427580	4.596328
C	-1.732548	0.516921	3.051358
C	-2.124282	0.060211	4.464683
H	-4.348209	-1.877804	0.166187
H	-4.158007	1.098814	-2.893494
H	3.804919	-2.806942	0.331766
H	1.048794	-4.414046	-2.510559
H	0.538877	4.704765	-0.130465
H	3.232859	2.849013	-2.879363
H	2.938826	-0.645377	1.625446
H	3.860363	-0.259145	0.181220
H	2.321612	0.552402	0.462697
H	3.229044	-5.582124	-2.013648
H	4.270303	-4.978326	-0.708619

H	2.769362	-5.832833	-0.326766
H	-0.367272	-3.072412	-3.614873
H	-1.503362	-2.598463	-2.342338
H	-0.640632	-1.363198	-3.239736
H	-2.140876	-2.114478	1.633146
H	-2.165087	-3.203314	0.255288
H	-0.715752	-2.253959	0.575997
H	-2.213957	1.577770	-3.903209
H	-1.327506	2.419178	-2.622176
H	-0.627906	0.996064	-3.371094
H	-6.291949	-0.186444	-2.452018
H	-6.383987	-1.271000	-1.049522
H	-6.393798	0.483127	-0.820843
H	2.746933	0.857754	-3.787779
H	2.938787	-0.236751	-2.409416
H	1.414060	-0.189699	-3.274510
H	-0.872819	3.019920	1.361820
H	-1.703775	3.474151	-0.117113
H	-1.627216	1.774021	0.338313
H	3.166526	5.360039	-2.617662
H	2.164062	6.081594	-1.342382
H	3.664054	5.241010	-0.927119
H	2.104948	1.037760	3.024802
H	1.065036	2.193013	2.219350
H	-0.372493	-2.118417	3.171876
H	1.202381	-1.850485	2.455769
H	-1.870656	1.597158	2.935295
H	-2.373533	0.046909	2.295898
H	-3.170998	0.317252	4.655314
H	-2.024990	-1.023621	4.578542
H	-1.518214	0.541888	5.237172
H	1.436571	-2.439827	4.879587
H	2.024024	-0.794973	4.620612
H	0.435159	-1.065281	5.357955
H	1.527773	2.927299	4.569570
H	-0.197127	2.594606	4.392281
H	0.851966	1.430094	5.219634

28. TPB NMR. R = CN

E_{elec} = -2205.421134 a.u.; **H =** -2204.915715 a.u

C	-2.047092	-1.786911	0.176044
C	-1.582475	-0.635318	-0.510895
C	-2.517115	-0.076656	-1.416997
C	-3.815844	-0.584239	-1.580821
C	-4.240734	-1.689463	-0.841826
C	-3.339632	-2.304436	0.030897
B	-0.081573	-0.027886	-0.212084
P	0.534619	-0.008037	2.809705
C	0.667216	1.731257	3.311980
C	1.315398	1.973752	4.685630
C	-2.217209	1.030023	-2.279368
N	-2.077268	1.905217	-3.031647
C	-5.569094	-2.196988	-0.987149
N	-6.650285	-2.608318	-1.095756
C	-1.181875	-2.574091	1.000669

N	-0.542457	-3.298745	1.646707
C	0.038607	1.550930	-0.660216
C	-0.693224	2.546775	0.033336
C	-0.577988	3.916072	-0.239518
C	0.271809	4.350927	-1.259623
C	0.948101	3.400656	-2.029069
C	0.821091	2.035853	-1.737993
C	-1.689501	2.200110	1.001373
N	-2.547234	2.000207	1.760355
C	1.481593	1.165710	-2.667527
N	2.018174	0.533631	-3.482485
C	0.418570	5.746518	-1.531585
N	0.541820	6.882617	-1.741663
C	1.167912	-0.929782	-0.780823
C	1.065457	-2.072392	-1.607716
C	2.182855	-2.830545	-1.991996
C	3.464763	-2.462349	-1.576243
C	3.623147	-1.308309	-0.803598
C	2.496240	-0.569874	-0.426673
C	-0.175517	-2.512105	-2.177211
N	-1.125654	-2.924289	-2.704613
C	4.603270	-3.242281	-1.949069
N	5.531723	-3.877909	-2.238668
C	2.761659	0.639131	0.293831
N	3.028684	1.615035	0.868017
C	2.144685	-0.790739	3.142100
C	2.308643	-2.252217	2.696788
C	-0.628756	-0.861181	3.909147
C	-2.102307	-0.452155	3.792805
H	1.565137	3.715909	-2.863127
H	-1.163015	4.632440	0.326993
H	2.046975	-3.700987	-2.624470
H	4.613525	-0.978764	-0.509014
H	-4.488553	-0.111771	-2.288113
H	-3.631237	-3.192249	0.581219
H	2.256165	-0.711147	4.232287
H	2.913298	-0.148030	2.704787
H	3.332754	-2.575439	2.906941
H	1.622209	-2.918892	3.221625
H	2.123068	-2.368846	1.627993
H	1.239422	2.225526	2.518950
H	-0.352440	2.129078	3.284154
H	1.278610	3.042150	4.916881
H	0.789980	1.444341	5.487236
H	2.365089	1.668214	4.695150
H	-0.245611	-0.681265	4.923111
H	-0.505080	-1.929726	3.704516
H	-2.701387	-1.072887	4.465696
H	-2.260921	0.595750	4.056319
H	-2.471224	-0.591425	2.774739
O	0.050423	-0.092278	1.349642

29. TPB NMR. R = 5F

E_{elec} = -2864.048755 a.u.; **H =** -2863.653054 a.u

C	-0.309403	-2.517502	-0.377188

C	-0.635013	-1.236115	-0.825180
C	-1.562474	-1.219269	-1.870125
C	-2.147322	-2.369630	-2.402249
C	-1.803690	-3.617952	-1.893365
C	-0.866993	-3.693271	-0.865593
B	0.059459	0.085210	-0.145198
O	-0.074327	-0.061662	1.408725
P	-0.441137	-0.645846	2.764443
C	-1.985224	-1.600445	2.710897
C	-2.353236	-2.313483	4.023137
F	-1.938244	-0.056155	-2.449930
F	-2.360708	-4.734057	-2.388340
F	0.633556	-2.669879	0.599950
C	-0.731936	1.481159	-0.480900
C	-2.040196	1.667839	-0.030030
C	-2.779089	2.830036	-0.212159
C	-2.205954	3.890761	-0.909518
C	-0.914812	3.752231	-1.408076
C	-0.212476	2.565361	-1.191084
F	-2.679758	0.652259	0.619631
F	1.025724	2.514203	-1.735663
F	-2.893811	5.027233	-1.102938
C	1.676263	0.178484	-0.385469
C	2.461658	1.008463	0.421763
C	3.844154	1.114178	0.317912
C	4.511706	0.373853	-0.654857
C	3.776759	-0.447992	-1.502777
C	2.391227	-0.524715	-1.356291
F	1.878123	1.789507	1.370786
F	5.846321	0.458225	-0.775442
F	1.753568	-1.329268	-2.241312
C	0.860402	-1.725393	3.437290
C	2.280310	-1.224897	3.125145
C	-0.611488	0.733782	3.932993
C	-1.500701	1.878355	3.424574
H	-2.771685	-0.907269	2.398415
H	-1.857295	-2.319027	1.892636
H	-3.295163	-2.854178	3.893059
H	-1.590377	-3.039958	4.318425
H	-2.489923	-1.604258	4.845803
H	-0.981661	0.319680	4.879540
H	0.407965	1.095045	4.116032
H	-1.540144	2.674816	4.173335
H	-1.093590	2.294149	2.499676
H	-2.523573	1.544436	3.228375
H	0.686482	-1.785268	4.519695
H	0.706564	-2.726653	3.023389
H	3.014187	-1.880444	3.602708
H	2.456281	-1.232244	2.047520
H	2.446205	-0.206396	3.489323
F	-0.355084	4.764660	-2.094568
F	-4.033783	2.937735	0.268448
F	-3.041101	-2.283839	-3.403185
F	-0.507404	-4.892229	-0.365701
F	4.540627	1.922873	1.140738

F	4.408117	-1.159105	-2.454861

30. TPB NMR. R = F

E_{elec} = -2268.393136 a.u.; **H =** -2267.955268 a.u

C	-0.463306	2.625482	-0.196761
C	0.515733	1.674393	-0.498682
C	1.703310	2.265332	-0.943427
C	1.938102	3.635046	-1.046589
C	0.902831	4.482766	-0.690873
C	-0.327733	4.004999	-0.262228
B	0.235507	0.073967	-0.314246
P	-1.232322	-0.379354	2.312830
C	-1.007432	-2.072234	2.945959
C	0.465608	-2.513014	2.952753
F	2.737935	1.473893	-1.336280
F	1.090910	5.825424	-0.773723
F	-1.703137	2.176062	0.187213
C	1.592033	-0.835806	-0.315975
C	2.549743	-0.724150	0.700248
C	3.713958	-1.473559	0.814089
C	3.954607	-2.404912	-0.185652
C	3.090832	-2.579798	-1.254026
C	1.948689	-1.783144	-1.280137
F	2.357914	0.209219	1.681694
F	1.143650	-1.962671	-2.365008
F	5.080535	-3.162480	-0.117934
C	-0.962126	-0.507860	-1.265951
C	-1.534847	0.131293	-2.372076
C	-2.577491	-0.370273	-3.148723
C	-3.080412	-1.614528	-2.808090
C	-2.568920	-2.350839	-1.748323
C	-1.529285	-1.764845	-1.038970
F	-1.053450	1.338370	-2.770427
F	-4.098142	-2.138197	-3.538154
F	-1.019782	-2.523960	-0.012519
C	-0.856048	0.727494	3.704718
C	-0.767636	2.209650	3.312530
C	-2.975989	-0.188003	1.838362
C	-3.992812	-0.619622	2.908026
H	3.292449	-3.295239	-2.041850
H	4.404487	-1.322773	1.634871
H	-2.966525	0.192083	-3.988806
H	-2.940669	-3.337878	-1.501644
H	2.890772	4.012652	-1.397409
H	-1.145572	4.669040	-0.010689
H	-3.107661	0.861646	1.559795
H	-3.096749	-0.770862	0.917583
H	-5.008647	-0.463521	2.532907
H	-3.890838	-1.679370	3.160897
H	-3.886886	-0.036234	3.828504
H	-1.610760	0.555084	4.482677
H	0.104700	0.381236	4.105207
H	-0.504183	2.809632	4.188782
H	-0.001355	2.356890	2.547683
H	-1.717361	2.581261	2.917157

H	-1.436977	-2.091558	3.956246
H	-1.604472	-2.742233	2.319993
H	0.549567	-3.513867	3.386663
H	0.858534	-2.538945	1.934437
H	1.092559	-1.834010	3.538967
O	-0.272934	-0.039588	1.186479

31. TPB NMR: R = H

E_{elec} = -1374.773745 a.u.; **H =** -1374.269935 a.u

C	3.273461	-1.114908	-0.067468
C	2.164035	-0.617988	-0.775879
C	2.265371	-0.599510	-2.176381
C	3.408445	-1.055186	-2.838871
C	4.493601	-1.543667	-2.110924
C	4.420779	-1.570994	-0.716213
B	0.884258	-0.053910	0.035021
C	1.176816	1.440757	0.591374
C	0.941647	2.588632	-0.187728
C	1.248585	3.871855	0.267417
C	1.818532	4.048889	1.529704
C	2.083873	2.928312	2.318274
C	1.767247	1.650938	1.851381
C	0.298267	-1.108444	1.133214
C	0.364739	-2.493322	0.883613
C	-0.267125	-3.427786	1.704812
C	-0.989995	-3.002413	2.822689
C	-1.049179	-1.639142	3.117796
C	-0.408175	-0.716177	2.285906
H	-1.588056	-1.295859	3.998528
H	-0.196897	-4.489191	1.476457
H	1.053126	4.735080	-0.365896
H	2.538268	3.049594	3.299314
H	3.452352	-1.028189	-3.926006
H	5.259232	-1.949428	-0.135256
H	-1.486312	-3.725783	3.465032
H	-0.455399	0.339704	2.546244
H	0.922102	-2.838708	0.015108
H	1.430526	-0.221659	-2.762327
H	3.234218	-1.150654	1.020403
H	5.384854	-1.899099	-2.622423
H	2.061042	5.045683	1.889678
H	1.986441	0.792193	2.483348
H	0.518392	2.471165	-1.184062
O	-0.280726	0.091319	-1.091454
P	-1.807579	0.174857	-0.927423
C	-2.424825	0.658660	-2.574441
C	-2.283093	1.454956	0.281099
C	-2.514789	-1.443699	-0.471446
H	-1.848844	-2.161057	-0.965021
H	-2.346996	-1.572253	0.603656
C	-3.979125	-1.719838	-0.846836
H	-1.456506	1.463013	0.998515
H	-2.225531	2.414727	-0.246013
C	-3.627493	1.281346	1.001737
C	-3.817016	1.307050	-2.643777

H	-1.660554	1.351573	-2.946488
H	-2.369783	-0.235679	-3.206935
H	-3.764506	2.100286	1.714587
H	-4.475977	1.295274	0.311235
H	-3.652161	0.343465	1.564684
H	-4.246405	-2.728548	-0.516944
H	-4.669863	-1.021384	-0.367216
H	-4.137296	-1.673430	-1.928770
H	-4.045577	1.573831	-3.680181
H	-4.604590	0.636963	-2.290477
H	-3.858595	2.224568	-2.049213

32. TPB NMR. R = NH_2

E_{elec} = -1873.287043 a.u.; **H =** -1872.623706 a.u

C	1.779073	-2.411943	-0.931188
C	1.556422	-1.189035	-0.226691
C	2.474206	-0.937668	0.836230
C	3.440307	-1.868851	1.242532
C	3.566440	-3.090734	0.575819
C	2.744910	-3.349175	-0.520215
B	0.320342	-0.147553	-0.552935
C	-1.003706	-0.845403	-1.231959
C	-1.569557	-0.479643	-2.489536
C	-2.802172	-0.996664	-2.932097
C	-3.485819	-1.946670	-2.173701
C	-2.912594	-2.408944	-0.983387
C	-1.690761	-1.884720	-0.541796
N	-0.864846	0.340930	-3.364404
N	-4.752698	-2.404868	-2.574875
N	-1.166570	-2.394898	0.663290
N	2.437871	0.277593	1.546359
N	4.475691	-4.061130	1.033518
N	1.108890	-2.681919	-2.121866
C	0.723173	1.359772	-1.070185
C	2.014469	1.752760	-1.531753
C	2.357808	3.105372	-1.723380
C	1.418703	4.115282	-1.523565
C	0.113055	3.765728	-1.160285
C	-0.220004	2.421330	-0.955114
N	2.959154	0.800178	-1.895958
N	-1.555252	2.142089	-0.584065
N	1.784548	5.467934	-1.640428
P	-1.111858	0.503471	2.148613
C	-2.864729	0.062828	1.870124
C	-3.915446	0.925803	2.585440
C	-0.486044	-0.479504	3.546300
C	-1.194630	-0.311521	4.897044
C	-1.041144	2.259738	2.628135
C	0.385477	2.830699	2.632808
H	-3.414011	-3.179665	-0.399067
H	-3.203264	-0.675726	-3.892570
H	4.092450	-1.634914	2.083233
H	2.867356	-4.271621	-1.086559
H	-0.644330	4.538102	-1.030432
H	3.361917	3.357380	-2.062478

H	-1.421701	0.851869	-4.037765
H	-0.122999	0.894114	-2.939177
H	-1.432018	-3.360650	0.826934
H	-0.154526	-2.277162	0.729984
H	-4.916347	-2.342126	-3.574058
H	-4.984625	-3.329883	-2.229031
H	-2.209920	2.822160	-0.957596
H	-1.836568	1.196224	-0.843036
H	3.924228	1.103644	-1.861072
H	2.808902	-0.129489	-1.510274
H	2.575864	5.628445	-2.254599
H	1.013970	6.083357	-1.878186
H	3.354833	0.568540	1.868654
H	1.976310	1.025528	1.036220
H	1.043803	-3.661853	-2.368461
H	0.236287	-2.177563	-2.252685
H	4.786124	-4.704926	0.313522
H	5.267561	-3.676069	1.537330
H	-0.549339	-1.514771	3.191383
H	0.581529	-0.233397	3.604348
H	-1.657254	2.797020	1.901604
H	-1.516153	2.352604	3.613404
H	-3.005336	0.100957	0.785451
H	-2.952363	-0.993739	2.143683
H	-4.916157	0.538185	2.368122
H	-3.786285	0.922074	3.672566
H	-3.879013	1.964246	2.243654
H	0.372692	3.857759	3.011818
H	1.062150	2.240307	3.258557
H	0.782459	2.852446	1.614574
H	-0.727472	-0.957520	5.647545
H	-1.133661	0.718119	5.264698
H	-2.252293	-0.588839	4.836651
O	-0.203597	0.211017	0.976265

33. TPB NMR. R = OCH_3

$\mathbf{E_{elec}}$ **=** -2406.061766 a.u.; **H =** -2405.248803 a.u

C	-1.423988	0.830631	1.826233
C	-0.755240	1.202536	0.641372
C	-0.869414	2.572571	0.339491
C	-1.578503	3.486599	1.132406
C	-2.222335	3.042848	2.283256
C	-2.151860	1.700971	2.649739
B	-0.092725	-0.038201	-0.231839
P	1.903279	1.204326	-1.837543
C	2.592740	2.032246	-0.356728
C	3.162617	3.451350	-0.470241
O	-0.226346	3.039657	-0.808526
C	-0.452507	4.394308	-1.181375
O	-2.901996	4.007835	2.998953
C	-3.589597	3.581040	4.172659
O	-1.298314	-0.496995	2.149724
C	-2.062339	-1.028902	3.219989
C	1.153317	-0.855060	0.476235
C	1.790245	-0.538003	1.686193

C	2.806186	-1.318705	2.273487
C	3.238111	-2.461504	1.608111
C	2.691532	-2.805589	0.369751
C	1.683570	-2.001510	-0.166221
O	1.405837	0.636479	2.296630
C	1.676832	0.819574	3.681095
O	1.147537	-2.279881	-1.401434
C	1.427156	-3.541569	-1.994687
O	4.218848	-3.306972	2.079724
C	4.801690	-2.989694	3.342616
C	-1.367753	-0.947123	-0.753858
C	-2.303592	-0.370308	-1.644516
C	-3.445606	-1.025840	-2.117181
C	-3.705219	-2.321734	-1.679397
C	-2.844549	-2.944661	-0.779813
C	-1.705331	-2.246949	-0.335542
O	-2.031633	0.922558	-2.037801
C	-3.021506	1.630551	-2.768527
O	-4.847156	-2.908332	-2.188178
C	-5.145384	-4.233468	-1.757970
O	-0.860171	-2.843706	0.576637
C	-1.090322	-4.189881	0.958231
C	3.167403	-0.008704	-2.347657
C	4.518601	0.564573	-2.802169
C	1.545662	2.250114	-3.297225
C	2.463600	3.405270	-3.722701
H	3.065440	-3.688591	-0.134297
H	3.250104	-1.019632	3.213555
H	-4.138898	-0.564999	-2.810012
H	-3.052187	-3.942692	-0.419308
H	-1.654528	4.537956	0.887104
H	-2.642635	1.335953	3.541851
H	-2.629219	2.644271	-2.880135
H	-3.186637	1.194177	-3.764500
H	-3.976321	1.665463	-2.225912
H	-0.255759	-4.448760	1.613862
H	-2.037200	-4.305100	1.507271
H	-1.096851	-4.867181	0.091464
H	-6.068264	-4.509953	-2.271742
H	-4.346015	-4.934935	-2.035726
H	-5.303582	-4.276575	-0.671101
H	-1.845972	-2.098602	3.221697
H	-1.766365	-0.595206	4.187027
H	-3.138436	-0.870376	3.063558
H	0.069600	4.535179	-2.130973
H	-1.521850	4.598669	-1.323258
H	-0.046474	5.097714	-0.440746
H	-4.063681	4.476650	4.578804
H	-4.358541	2.832688	3.936099
H	-2.894628	3.164877	4.915675
H	1.097409	1.697839	3.973809
H	1.350079	-0.050402	4.265955
H	2.744111	1.007177	3.870561
H	0.788117	-3.594350	-2.878227
H	2.481692	-3.624886	-2.297110

H	1.181642	-4.365382	-1.311906
H	5.530002	-3.779041	3.537329
H	5.312415	-2.017080	3.314188
H	4.045868	-2.980152	4.139805
H	1.466047	1.510257	-4.105241
H	0.526357	2.597020	-3.106958
H	1.775906	2.009422	0.370808
H	3.353850	1.339038	0.021494
H	3.298509	-0.660179	-1.476203
H	2.705992	-0.622066	-3.129460
H	3.546081	3.758988	0.508266
H	3.985886	3.520184	-1.187928
H	2.387865	4.164578	-0.760383
H	2.146481	3.775428	-4.703540
H	2.411787	4.239898	-3.020918
H	3.510926	3.104177	-3.806415
H	5.234380	-0.250224	-2.951522
H	4.435659	1.108352	-3.747734
H	4.944951	1.242281	-2.054396
O	0.566647	0.506654	-1.578545

34. TPB NMR. R = $OCOCH_3$

E_{elec} = -2406.061766 a.u.; **H =** -2405.248803 a.u

C	-1.835839	1.792348	1.074443
C	-0.561210	1.537580	0.529435
C	0.130566	2.721929	0.230840
C	-0.357898	4.012583	0.428924
C	-1.648699	4.170899	0.912714
C	-2.402175	3.050069	1.237320
B	-0.054813	-0.007189	0.243145
P	-0.020876	-1.962906	2.546346
C	1.656312	-2.640168	2.365341
C	2.097773	-3.694247	3.395135
O	1.475596	2.686212	-0.196415
C	1.828903	2.574533	-1.511834
O	3.011750	2.541748	-1.776651
O	-2.173119	5.430455	1.177189
C	-2.592768	6.284011	0.151399
O	-2.997621	7.369965	0.471317
O	-2.654360	0.692101	1.391843
C	-2.830853	0.325962	2.702696
O	-3.435819	-0.699577	2.928064
C	1.551853	-0.166572	-0.089809
C	2.117205	-0.763570	-1.228712
C	3.462859	-1.114529	-1.351285
C	4.333628	-0.768226	-0.330261
C	3.867915	-0.101908	0.796999
C	2.512578	0.202821	0.877116
O	1.246110	-1.111677	-2.269808
C	1.439673	-0.826082	-3.606120
O	0.602969	-1.249511	-4.371449
O	2.027332	0.821385	2.030199
C	2.481872	2.028350	2.543625
O	1.896514	2.447281	3.513297
O	5.671626	-1.146158	-0.396564

C	6.612555	-0.315500	-1.014515
O	7.756386	-0.683909	-1.024848
C	-1.114378	-0.812212	-0.764620
C	-1.113176	-2.219218	-0.831316
C	-2.177475	-3.008329	-1.259663
C	-3.316168	-2.374096	-1.738373
C	-3.330311	-0.995591	-1.868666
C	-2.228809	-0.260352	-1.426834
O	0.010380	-2.881186	-0.315982
C	0.721803	-3.832537	-1.033267
O	1.600265	-4.409059	-0.433826
O	-4.421740	-3.122808	-2.131292
C	-5.405326	-3.454604	-1.191831
O	-6.348923	-4.090781	-1.577609
O	-2.313446	1.127133	-1.600051
C	-2.438619	1.687416	-2.857107
O	-2.688273	2.868970	-2.913472
C	0.380228	-4.080400	-2.479750
C	-5.172296	-2.988062	0.225740
C	-2.196574	0.810177	-4.062703
C	3.655175	2.725887	1.900117
C	6.088059	0.963908	-1.618415
C	2.630612	-0.005649	-4.037136
C	0.705451	2.472876	-2.505545
C	-2.258840	1.238420	3.762191
C	-2.503743	5.753561	-1.256560
C	-1.370936	-3.176230	2.299132
C	-1.006267	-4.666104	2.248074
C	-0.183279	-1.508553	4.297340
C	0.853175	-0.530193	4.860645
H	-3.415068	3.157692	1.609300
H	0.273092	4.868995	0.217586
H	-2.136265	-4.089603	-1.192035
H	-4.201229	-0.490611	-2.271911
H	4.551036	0.147042	1.600444
H	3.825130	-1.666320	-2.210160
H	-1.461650	0.024384	-3.875167
H	-3.130947	0.325524	-4.363041
H	-1.871327	1.457351	-4.878325
H	-6.053703	-3.235903	0.815792
H	-4.295395	-3.490178	0.647600
H	-4.970983	-1.914187	0.275312
H	-0.321758	-4.918756	-2.543683
H	-0.056174	-3.213347	-2.974556
H	1.303327	-4.380975	-2.978362
H	2.967784	0.701205	-3.276978
H	3.459340	-0.678889	-4.281147
H	2.347433	0.516747	-4.952257
H	3.738625	2.527215	0.832096
H	3.529113	3.794809	2.077075
H	4.574553	2.405219	2.403349
H	5.509514	1.545692	-0.895717
H	6.935608	1.542609	-1.983632
H	5.407209	0.742538	-2.446245
H	0.213385	1.503843	-2.367201

H	-0.054497	3.239300	-2.336074
H	1.106847	2.547767	-3.514914
H	-1.202288	1.446993	3.569104
H	-2.781275	2.199235	3.760976
H	-2.389720	0.765118	4.735013
H	-2.882051	4.731868	-1.343858
H	-3.061340	6.426288	-1.907718
H	-1.457369	5.732685	-1.579886
H	-2.094401	-2.971732	3.096067
H	-1.858683	-2.861895	1.373807
H	-1.889276	-5.242772	1.952933
H	-0.212065	-4.863211	1.523230
H	-0.685618	-5.039328	3.225070
H	-1.204589	-1.134212	4.417726
H	-0.152124	-2.470673	4.829459
H	0.643740	-0.341272	5.918302
H	1.867378	-0.935634	4.789028
H	0.840266	0.423535	4.331257
H	1.762094	-3.026417	1.349734
H	2.296046	-1.755189	2.444335
H	3.148329	-3.939522	3.212240
H	2.015679	-3.330525	4.423865
H	1.525748	-4.619453	3.307590
O	-0.254326	-0.713820	1.667836

35. TPB NMR. R = OH

E_{elec} = -2052.131057 a.u.; **H =** -2051.582705 a.u

C	-0.033317	-0.083804	2.805766
C	0.031235	-0.617560	1.506087
C	0.533719	-1.929325	1.464816
C	0.991748	-2.649868	2.574575
C	0.923421	-2.053167	3.828111
C	0.408844	-0.765893	3.950449
B	-0.489939	0.120065	0.133159
P	2.148846	-0.206712	-1.325098
C	2.138838	-1.602868	-2.501455
C	3.254605	-1.691194	-3.552558
O	0.604595	-2.550888	0.220646
O	1.379725	-2.779413	4.909958
O	-0.592156	1.163007	2.969798
C	-1.692418	-0.724160	-0.592469
C	-2.676678	-1.403419	0.147122
C	-3.700369	-2.173357	-0.423419
C	-3.786150	-2.266952	-1.809108
C	-2.870663	-1.585564	-2.599965
C	-1.868788	-0.823725	-1.983677
O	-2.652995	-1.264776	1.520274
O	-1.016677	-0.111094	-2.824542
O	-4.761106	-3.010375	-2.447604
C	-0.722066	1.737170	0.232092
C	0.341503	2.594899	0.561050
C	0.260162	3.988159	0.627609
C	-0.965994	4.593973	0.374729
C	-2.069780	3.808815	0.060858
C	-1.932751	2.411402	-0.003498

O	1.574112	2.010081	0.851688
O	-1.026865	5.971539	0.449194
O	-3.055048	1.664152	-0.284991
C	2.691212	1.299943	-2.202541
C	4.200882	1.524454	-2.375748
C	3.301844	-0.536288	0.052031
C	4.539864	-1.389052	-0.261180
H	-2.946220	-1.646656	-3.683728
H	-4.429840	-2.675930	0.213121
H	1.125421	4.599383	0.874677
H	-3.038786	4.271610	-0.130860
H	1.391594	-3.656270	2.473945
H	0.335626	-0.292828	4.930735
H	2.116897	2.691759	1.278272
H	-1.934837	6.246258	0.245055
H	-3.796976	2.274112	-0.420963
H	-0.608020	1.364834	3.918511
H	0.757615	-3.496085	0.378180
H	1.264169	-2.240250	5.708417
H	-3.382981	-1.785931	1.889103
H	-1.404357	-0.161724	-3.712893
H	-5.318523	-3.422721	-1.768935
H	2.080633	-2.510127	-1.891626
H	1.157596	-1.489310	-2.979193
H	2.170969	1.295626	-3.168035
H	2.246099	2.105834	-1.608671
H	3.578742	0.442019	0.456876
H	2.683214	-1.015899	0.817555
H	5.122327	-1.537710	0.653876
H	4.257926	-2.377775	-0.636320
H	5.195418	-0.917858	-0.999911
H	3.085489	-2.561539	-4.195327
H	3.269002	-0.806136	-4.196522
H	4.245300	-1.800185	-3.102260
H	4.377684	2.474879	-2.889792
H	4.707774	1.574390	-1.406769
H	4.675577	0.734840	-2.964831
O	0.726297	0.013270	-0.883054

36. H_3PO_4 NMR

E_{elec} = -644.372730 a.u.; **H =** -644.319634 a.u

P	-0.053721	0.149720	0.000000
O	-0.545822	1.521184	0.000000
O	1.557429	0.070397	-0.000000
O	-0.545822	-0.684257	1.290114
O	-0.545822	-0.684257	-1.290114
H	-0.251527	-1.606650	1.349877
H	-0.251527	-1.606650	-1.349877
H	1.949163	-0.817039	-0.000000

37. $PO(CH_2CH_3)_3$ NMR

E_{elec} = -654.5192712 a.u.; **H =** -654.305918 a.u

O	0.841651	-0.959892	1.700354
P	0.142116	-0.306739	0.549937
C	-1.592141	-0.894494	0.356625

C	0.962808	-0.611853	-1.061446
C	0.074633	1.516760	0.753034
C	-2.239440	-0.830501	-1.037059
H	-1.555978	-1.931616	0.711796
H	-2.189364	-0.339115	1.091279
H	1.120926	1.828840	0.858831
C	-0.637592	2.350372	-0.322313
H	-0.387631	1.671027	1.736217
C	2.446751	-0.211644	-1.035656
H	0.863581	-1.688922	-1.248131
H	0.423326	-0.094282	-1.864138
H	-3.272442	-1.192104	-0.990417
H	-2.262164	0.187152	-1.437368
H	-1.701532	-1.459413	-1.753115
H	2.946933	-0.525229	-1.957891
H	2.565483	0.873565	-0.945047
H	2.953280	-0.680338	-0.187001
H	-0.542357	3.418831	-0.100480
H	-0.210906	2.180754	-1.316669
H	-1.706225	2.119219	-0.368858

2. TPP derivatives. Cartesian coordinates

38. TPP R = CF_3

E_{elec} = -4071.662342 a.u.; **H =** -4071.308043 a.u

C	-1.462312	-2.093486	-0.859613
C	-0.505082	-1.700533	0.113004
C	0.023033	-2.740534	0.933960
C	-0.312644	-4.075865	0.728225
C	-1.223719	-4.427342	-0.257463
C	-1.812198	-3.435744	-1.025058
P	0.004674	0.002081	0.654877
C	1.731638	0.414890	0.107474
C	2.372781	1.386320	0.932564
C	3.696969	1.761330	0.725502
C	4.453251	1.152383	-0.266187
C	3.884302	0.154050	-1.039008
C	2.546024	-0.211899	-0.872145
C	-1.218468	1.294451	0.119771
C	-2.374630	1.362549	0.953233
C	-3.358686	2.327464	0.760714
C	-3.211115	3.295823	-0.222843
C	-2.069058	3.301199	-1.005699
C	-1.085669	2.319880	-0.852847
H	-1.938836	4.074270	-1.751769
H	-4.231114	2.340498	1.401021
H	4.144332	2.522224	1.351763
H	4.480505	-0.337259	-1.797039
H	0.128703	-4.843188	1.350728
H	-2.540630	-3.707542	-1.778150
C	-1.622960	-5.871372	-0.442131
C	0.931563	-2.488907	2.139332
C	-2.251477	-1.147195	-1.756836
C	2.116513	-1.360603	-1.777062
C	5.903152	1.530950	-0.447941

C	1.705611	2.041736	2.143681
C	0.119589	2.526890	-1.762414
C	-4.299564	4.318373	-0.442359
C	-2.601619	0.451770	2.161589
F	-4.890441	4.681158	0.724146
F	-3.828869	5.445129	-1.030716
F	-5.280931	3.826828	-1.247227
F	-3.836474	0.631807	2.695973
F	-2.520825	-0.872585	1.860784
F	-1.714574	0.703412	3.151716
F	1.131773	3.153804	-1.094228
F	0.625063	1.393808	-2.286611
F	-0.182580	3.324955	-2.818971
F	2.037123	-1.755492	1.837696
F	1.394960	-3.651593	2.664994
F	0.275053	-1.852224	3.136406
F	-2.008856	-6.130534	-1.715931
F	-0.606518	-6.716950	-0.140855
F	-2.668052	-6.202148	0.365453
F	-3.293152	-0.587021	-1.075421
F	-2.802140	-1.801244	-2.811898
F	-1.526622	-0.140453	-2.282064
F	1.484450	1.149977	3.136705
F	2.483931	3.020546	2.671935
F	0.517504	2.635828	1.849284
F	6.110740	2.849680	-0.206657
F	6.343012	1.269105	-1.703052
F	6.705113	0.840190	0.408442
F	2.160822	-2.549471	-1.107114
F	0.877788	-1.234686	-2.290981
F	2.949990	-1.498460	-2.840292

39. TPP R = CH_3

E_{elec} = -1390.771860 a.u.; **H =** -1390.226779 a.u

P	0.001406	-0.001425	0.791276
C	-1.277880	-1.175717	0.202457
C	-2.255384	-0.939534	-0.795749
C	-1.332246	-2.388982	0.942082
C	-3.262726	-1.890063	-0.998948
C	-2.346383	-3.313305	0.685231
C	-3.336141	-3.078275	-0.271376
H	-4.004622	-1.698590	-1.773183
H	-2.362870	-4.241685	1.254407
C	-0.378933	1.693236	0.203866
C	-1.405822	2.343437	0.941868
C	0.314407	2.424461	-0.792184
C	-1.703192	3.682941	0.684651
C	-0.009238	3.771014	-0.995929
C	-1.005566	4.425120	-0.270510
H	-2.501635	4.158536	1.252470
H	0.527476	4.319787	-1.768778
C	1.658898	-0.520016	0.203138
C	2.735608	0.046522	0.939287
C	1.945701	-1.488545	-0.790666
C	4.044420	-0.365850	0.682885

C	3.273863	-1.881652	-0.993262
C	4.338497	-1.343731	-0.269465
H	4.855445	0.089701	1.249308
H	3.480910	-2.622733	-1.764268
C	2.508628	1.122255	1.975223
H	2.162418	2.056233	1.514504
H	1.732846	0.822114	2.690478
H	3.434026	1.334241	2.518984
C	5.755161	-1.812277	-0.496606
H	6.472874	-0.999184	-0.345329
H	6.016763	-2.615224	0.205140
H	5.886856	-2.205208	-1.509877
C	0.900357	-2.095398	-1.696845
H	0.210801	-1.337997	-2.080785
H	1.385382	-2.581744	-2.548734
H	0.287294	-2.844185	-1.183769
C	1.361040	1.820908	-1.698900
H	2.317773	1.668944	-1.187563
H	1.051119	0.842518	-2.077315
H	1.535922	2.480703	-2.554236
C	-1.308242	5.886096	-0.498657
H	-0.741686	6.514846	0.200846
H	-1.036259	6.195277	-1.512986
H	-2.370778	6.101663	-0.344929
C	-2.221633	1.610475	1.980708
H	-2.857588	0.842226	1.522762
H	-1.572249	1.090800	2.695968
H	-2.867523	2.306608	2.524049
C	-0.292110	-2.729984	1.983137
H	0.692524	-2.895023	1.527463
H	-0.169274	-1.908791	2.700024
H	-0.572739	-3.638505	2.524266
C	-4.452986	-4.067581	-0.499489
H	-5.283015	-3.884550	0.195595
H	-4.852096	-3.989942	-1.515939
H	-4.113147	-5.096145	-0.339294
C	-2.250674	0.266505	-1.705431
H	-2.593945	1.173904	-1.196951
H	-1.247209	0.482011	-2.083683
H	-2.909444	0.088410	-2.560877

40. TPP R = CN

E_{elec} = -1867.332737 a.u.; **H =** -1867.046378 a.u

C	-0.433136	-2.701147	0.937360
C	-0.806226	-1.499927	0.282522
C	-1.831643	-1.580832	-0.685686
C	-2.459324	-2.801780	-0.968703
C	-2.068488	-3.971554	-0.308966
C	-1.040613	-3.922819	0.640580
P	0.000134	0.000061	0.965680
C	-0.895821	1.448408	0.282771
C	-2.123026	1.725257	0.937220
C	-2.877671	2.861911	0.640414
C	-2.405968	3.776902	-0.308713
C	-1.197045	3.531259	-0.967900

C	-0.453107	2.377456	-0.684881
C	1.702368	0.051657	0.282609
C	2.556235	0.975165	0.937592
C	3.917940	1.059930	0.640694
C	4.473949	0.194336	-0.309130
C	3.656221	-0.728882	-0.968932
C	2.285036	-0.795787	-0.685786
H	-3.239637	-2.837144	-1.720811
H	-0.718488	-4.824672	1.148856
H	-3.820057	3.033311	1.148329
H	-0.837456	4.225106	-1.719617
H	4.537987	1.789697	1.149052
H	4.076897	-1.386838	-1.721218
C	5.869639	0.259209	-0.610038
C	2.003543	1.887088	1.893260
C	1.522923	-1.717809	-1.469327
C	0.727064	2.179465	-1.467754
C	-3.160417	4.952925	-0.609505
C	-2.636537	0.790050	1.892243
C	-2.249168	-0.459774	-1.469127
C	0.633165	-2.678416	1.892798
C	-2.710225	-5.212705	-0.609670
N	1.554034	2.660250	2.636427
N	7.004670	0.311381	-0.852019
N	0.959637	-2.476220	-2.146108
N	1.527670	-2.675667	2.635759
N	-3.232608	-6.221777	-0.851464
N	-2.624364	0.407332	-2.145785
N	1.666018	2.071653	-2.143959
N	-3.081470	0.013753	2.634896
N	-3.773482	5.909597	-0.851383

41. TPP R = 5F

E_{elec} = -2525.969597 a.u.; **H =** -2525.792012 a.u

C	-0.459648	-2.138976	-0.634740
C	0.251870	-1.560732	0.422287
C	1.281747	-2.336957	0.971931
C	1.595673	-3.612517	0.517071
C	0.852837	-4.160620	-0.527847
C	-0.178086	-3.421079	-1.103406
P	-0.019196	0.062197	1.271638
C	-1.617112	0.671874	0.585921
C	-2.752696	-0.077744	0.919982
C	-4.045595	0.315894	0.602944
C	-4.241395	1.528365	-0.058394
C	-3.141567	2.316559	-0.387167
C	-1.855003	1.887786	-0.062179
F	-2.589742	-1.255715	1.562664
F	-5.477979	1.933447	-0.369122
F	-0.843113	2.713138	-0.399892
F	2.024031	-1.829964	1.979451
F	1.133740	-5.387467	-0.980445
F	-1.448780	-1.475069	-1.259299
C	1.282948	1.054971	0.428471
C	1.516464	1.072324	-0.951343

C	2.576286	1.769361	-1.517864
C	3.442987	2.484669	-0.689011
C	3.240039	2.496376	0.689307
C	2.169000	1.785605	1.228221
F	0.682668	0.420092	-1.781683
F	2.007209	1.825149	2.562215
F	4.465393	3.161914	-1.220459
F	-3.324866	3.487540	-1.015762
F	-5.096633	-0.451432	0.925415
F	4.072755	3.186451	1.481574
F	2.768721	1.773688	-2.845195
F	2.596954	-4.312983	1.069555
F	-0.884196	-3.942556	-2.117869

42. TPP R = F

E_{elec} = -1930.325552 a.u.; **H =** -1930.105606 a.u

C	-1.469521	-1.172305	-1.030401
C	-1.208167	-1.126071	0.345514
C	-2.057177	-1.913875	1.133505
C	-3.094425	-2.687929	0.626984
C	-3.281614	-2.664808	-0.747647
C	-2.485157	-1.917603	-1.608185
P	0.039979	-0.058364	1.172215
C	1.662488	-0.559224	0.464805
C	2.766580	0.248492	0.775070
C	4.081915	-0.038795	0.452723
C	4.311212	-1.237260	-0.211608
C	3.288968	-2.114963	-0.539411
C	1.993648	-1.752903	-0.187329
C	-0.351414	1.546119	0.341549
C	-1.426421	2.261429	0.890731
C	-1.851916	3.510304	0.464840
C	-1.142980	4.084011	-0.582491
C	-0.069124	3.453075	-1.193374
C	0.291699	2.197197	-0.719552
H	0.466144	3.909960	-2.016797
H	-2.695048	4.007153	0.928543
H	4.886012	0.640059	0.708294
H	3.482973	-3.050472	-1.049657
H	-3.724229	-3.280169	1.279169
H	-2.641496	-1.924028	-2.679914
F	-1.519546	5.303896	-1.029587
F	1.325231	1.595259	-1.348702
F	-2.117227	1.682460	1.905324
F	2.521628	1.412853	1.430641
F	5.581266	-1.562333	-0.546060
F	1.018000	-2.639436	-0.503059
F	-0.673710	-0.461574	-1.859663
F	-1.868631	-1.938527	2.471392
F	-4.280969	-3.405531	-1.276641

43. TPP R = H

E_{elec} = -1036.733729 a.u.; **H =** -1036.447906 a.u

C	2.431734	-1.319462	-0.984596
C	1.620707	-0.311987	-0.436277

C	2.087578	0.395582	0.680771
C	3.331836	0.097076	1.238864
C	4.121992	-0.916712	0.695817
C	3.667084	-1.627261	-0.417369
P	0.000582	-0.000621	-1.257588
C	-0.539481	1.558149	-0.436213
C	-0.071987	2.764049	-0.984519
C	-0.422660	3.988007	-0.417437
C	-1.265487	4.027129	0.695675
C	-1.748916	2.836107	1.238701
C	-1.385925	1.609115	0.680618
C	-1.079965	-1.247598	-0.436793
C	-2.360403	-1.439506	-0.981829
C	-3.246508	-2.353916	-0.415033
C	-2.858076	-3.108488	0.694326
C	-1.582515	-2.938066	1.233767
C	-0.699934	-2.011255	0.676202
H	3.681412	0.655938	2.103326
H	4.279571	-2.414026	-0.850108
H	-0.047079	4.911621	-0.850187
H	-2.407756	2.859701	2.103112
H	-4.236015	-2.485512	-0.845099
H	-1.272797	-3.524506	2.095156
H	-3.543947	-3.829468	1.131051
H	-2.662029	-0.864213	-1.854643
H	0.288253	-1.880538	1.108087
H	-1.761303	0.687014	1.115199
H	-1.548434	4.981086	1.132558
H	0.573193	2.739004	-1.860223
H	1.476255	1.181462	1.115198
H	5.089674	-1.148297	1.132762
H	2.087842	-1.865728	-1.860417

44. TPP R = NH_2

E_{elec} = -1535.243030 a.u.; **H =** -1534.798086 a.u

C	-4.259390	-1.537641	-0.414353
C	-3.750061	-1.736306	0.874992
C	-2.467444	-1.285962	1.201730
C	-1.629036	-0.658582	0.224704
C	-2.186975	-0.436194	-1.073579
C	-3.485925	-0.881819	-1.373678
P	-0.020765	-0.074748	0.858677
C	1.392160	-1.089759	0.275185
C	2.650183	-0.764013	0.867974
C	3.785935	-1.552065	0.645127
C	3.702440	-2.707486	-0.138144
C	2.481675	-3.052481	-0.723050
C	1.339090	-2.259158	-0.535611
C	0.245993	1.623866	0.232401
C	0.959909	2.000599	-0.934902
C	1.135550	3.344724	-1.278953
C	0.574641	4.355633	-0.489435
C	-0.136555	4.015091	0.664615
C	-0.291806	2.673358	1.036671
H	1.682733	3.600735	-2.184752

H	-0.566046	4.796522	1.288877
H	4.732244	-1.268379	1.102567
H	2.408827	-3.946207	-1.340600
H	-4.360077	-2.221732	1.634147
H	-3.886808	-0.715499	-2.372116
N	-5.516760	-2.046983	-0.753932
H	-6.151313	-2.169086	0.026745
H	-5.967104	-1.582837	-1.534202
N	-1.488256	0.223783	-2.052902
H	-0.475250	0.327799	-1.994258
H	-1.831810	0.112003	-2.996824
N	-2.044619	-1.411760	2.522326
H	-2.456009	-2.171494	3.048893
H	-1.049535	-1.271398	2.677739
N	2.754108	0.334050	1.717824
N	4.818017	-3.543150	-0.286328
N	0.183015	-2.607477	-1.223866
H	3.700754	0.632912	1.916630
H	2.127525	1.113109	1.533409
H	4.811208	-4.110201	-1.126631
H	5.710860	-3.080211	-0.159944
H	0.136210	-3.575964	-1.515111
H	-0.689358	-2.266112	-0.826744
N	1.417989	1.014564	-1.821340
N	0.675842	5.694274	-0.884689
N	-0.938476	2.389165	2.231466
H	2.071062	1.356114	-2.517478
H	1.744226	0.157864	-1.370327
H	0.589753	6.367751	-0.132248
H	1.466166	5.902122	-1.483897
H	-1.611152	3.073020	2.551032
H	-1.213956	1.424293	2.384864

45. TPP R = NO_2

E_{elec} = -2878.319118 a.u.; **H =** -2877.995214 a.u

C	0.892796	2.301301	-0.621289
C	-0.051566	1.727186	0.254719
C	-0.907211	2.686176	0.851448
C	-0.885572	4.047409	0.570907
C	0.053049	4.509987	-0.330080
C	0.971676	3.653609	-0.917115
P	-0.000155	-0.000222	0.973218
C	-1.470047	-0.908578	0.254265
C	-1.873094	-2.128862	0.851154
C	-3.062965	-2.790447	0.570650
C	-3.932736	-2.208706	-0.330341
C	-3.649975	-0.985178	-0.917642
C	-2.439183	-0.377721	-0.621957
C	1.521816	-0.819004	0.254812
C	2.780103	-0.557757	0.851720
C	3.948144	-1.257037	0.570881
C	3.879455	-2.300885	-0.330472
C	2.678499	-2.668034	-0.917627
C	1.546868	-1.923571	-0.621590
H	2.631675	-3.510167	-1.598382

H	4.879015	-0.988902	1.054524
H	-3.296370	-3.730588	1.054384
H	-4.355905	-0.523421	-1.598274
H	-1.583386	4.719423	1.054431
H	1.724530	4.034294	-1.597636
N	0.093391	5.958130	-0.664884
N	-1.897827	2.279105	1.870914
N	1.923787	1.499681	-1.340654
N	-2.259590	0.915340	-1.342157
N	-5.207354	-2.897421	-0.664763
N	-1.025585	-2.783109	1.870938
N	0.337056	-2.415089	-1.341019
N	5.113428	-3.059799	-0.665456
N	2.922854	0.503220	1.871634
O	0.957332	6.320849	-1.463121
O	-0.737553	6.681208	-0.118333
O	-2.433104	3.143206	2.554287
O	-2.107334	1.059546	1.980348
O	3.095856	1.754057	-1.053538
O	1.542235	0.690740	-2.176325
O	-3.065389	1.803962	-1.055925
O	-1.368045	0.988040	-2.177738
O	-5.953383	-2.330502	-1.463006
O	-5.418392	-3.978379	-0.117936
O	-1.506551	-3.678456	2.554470
O	0.135359	-2.354866	1.980536
O	-0.027661	-3.558150	-1.055930
O	-0.174133	-1.678652	-2.174519
O	3.938891	0.534522	2.554932
O	1.971322	1.294228	1.981578
O	6.155073	-2.701929	-0.118709
O	4.995640	-3.989056	-1.464050

46. TPP R = OCH_3

E_{elec} = -2068.028114 a.u.; **H =** -2067.432836 a.u

C	1.109268	1.467479	-0.921909
C	0.958797	1.306585	0.465026
C	1.719150	2.161404	1.289369
C	2.600047	3.110138	0.762078
C	2.727629	3.219563	-0.623902
C	1.985867	2.407395	-1.484388
P	-0.042828	-0.021383	1.259056
C	-1.787358	0.215452	0.716047
C	-2.673964	-0.833439	1.049910
C	-4.038502	-0.794518	0.774851
C	-4.565735	0.350468	0.172126
C	-3.748235	1.438447	-0.128351
C	-2.373545	1.363568	0.161225
C	0.607830	-1.470640	0.298921
C	1.883555	-1.896980	0.714920
C	2.533177	-3.022155	0.187628
C	1.876178	-3.759187	-0.797858
C	0.616071	-3.373611	-1.256391
C	0.002573	-2.235986	-0.721976
H	0.152097	-3.963022	-2.037646

H	3.516386	-3.304234	0.538153
H	-4.704986	-1.613788	1.014344
H	-4.161791	2.338197	-0.562583
H	3.189768	3.768505	1.387471
H	2.076093	2.503027	-2.557914
O	3.616693	4.174454	-1.056091
O	1.539153	2.008305	2.637179
O	0.322947	0.662346	-1.691220
O	-1.544115	2.427929	-0.072844
O	-5.918829	0.315564	-0.076035
O	-2.071588	-1.909002	1.646852
O	-1.209307	-1.803848	-1.181152
O	2.400728	-4.883807	-1.391154
O	2.462345	-1.111873	1.679101
C	2.327197	2.806009	3.517758
H	2.036643	2.497031	4.523218
H	3.400386	2.623244	3.369450
H	2.117675	3.876168	3.386183
C	3.790245	4.326468	-2.465458
H	4.531829	5.118051	-2.584841
H	4.162466	3.400139	-2.923119
H	2.852078	4.624651	-2.952815
C	0.484200	0.682591	-3.104913
H	-0.206502	-0.073834	-3.480381
H	0.220322	1.663184	-3.525388
H	1.510885	0.421226	-3.394042
C	-2.860060	-3.062051	1.929520
H	-2.167774	-3.787489	2.359298
H	-3.655642	-2.834994	2.651542
H	-3.302853	-3.470854	1.011481
C	-2.037528	3.540697	-0.810002
H	-1.174729	4.194866	-0.948740
H	-2.427511	3.229714	-1.789091
H	-2.820114	4.078886	-0.257194
C	-6.506317	1.469022	-0.677487
H	-7.566233	1.233977	-0.788579
H	-6.390648	2.354520	-0.037808
H	-6.068195	1.671164	-1.664487
C	3.754510	-1.455794	2.168919
H	3.987220	-0.698331	2.919204
H	3.754990	-2.449830	2.636551
H	4.505706	-1.423629	1.368080
C	-1.933115	-2.627159	-2.089724
H	-2.893491	-2.126067	-2.223187
H	-1.418963	-2.710181	-3.057707
H	-2.094056	-3.632871	-1.678548
C	3.689365	-5.316658	-0.954570
H	3.911433	-6.211097	-1.539025
H	4.453616	-4.551589	-1.147852
H	3.684286	-5.567186	0.114851

47. TPP R = $OCOCH_3$

E_{elec} = -3088.765565 a.u.; **H =** -3088.074599 a.u

C	2.282186	-0.987919	-0.614792
C	1.724238	-0.345114	0.501554

C	2.632233	0.083119	1.490959
C	4.003976	-0.090326	1.387519
C	4.501632	-0.768680	0.277246
C	3.649586	-1.242637	-0.715026
P	-0.032857	-0.220154	0.997629
C	-1.032045	-1.312121	-0.084823
C	-1.028430	-2.663130	0.313806
C	-2.000378	-3.575127	-0.071094
C	-3.025849	-3.129010	-0.901122
C	-3.033042	-1.829105	-1.394511
C	-2.023297	-0.948898	-1.012457
O	-0.038958	-3.031196	1.222913
C	1.023800	-3.875050	0.881946
C	0.990856	-4.528165	-0.475371
O	-4.036725	-4.001702	-1.276124
C	-5.190024	-4.131708	-0.478748
C	-5.241772	-3.284919	0.767614
O	-2.042528	0.364678	-1.464144
C	-2.121078	0.729832	-2.800224
C	-2.014190	-0.337589	-3.859102
O	2.113381	0.796283	2.565142
C	1.749509	0.148478	3.763342
C	2.132777	-1.298577	3.887561
O	5.858370	-1.046669	0.202336
C	6.735522	-0.202911	-0.499190
C	6.118739	1.005498	-1.154030
O	1.409718	-1.471567	-1.583736
C	1.597153	-1.350139	-2.953726
C	2.631630	-0.382684	-3.473691
C	-0.602004	1.501218	0.726337
C	0.158093	2.629876	0.382589
C	-0.360873	3.922061	0.420074
C	-1.696183	4.107316	0.760265
C	-2.493652	3.025199	1.125495
C	-1.930497	1.758320	1.125900
O	1.505507	2.474024	0.052693
C	1.952829	2.586586	-1.246780
C	0.949940	2.961792	-2.307238
O	-2.746103	0.666309	1.411623
C	-2.903697	0.171939	2.718697
C	-2.251183	0.964594	3.816349
O	-2.227494	5.389321	0.794485
C	-2.808746	5.949730	-0.353959
C	-2.795448	5.095990	-1.597448
O	-2.234227	1.911583	-3.025966
O	-6.031059	-4.900814	-0.854354
O	1.881169	-4.024720	1.712618
O	0.870413	-2.012033	-3.653669
O	1.173553	0.813751	4.581580
O	7.894664	-0.513047	-0.524195
O	-3.558263	-0.829079	2.838715
O	3.125967	2.374774	-1.448061
O	-3.261881	7.057179	-0.258966
H	-3.534245	3.165730	1.396484
H	0.268838	4.771756	0.179729

H	-1.989469	-4.594555	0.297170
H	-3.838600	-1.504283	-2.042527
H	4.669971	0.281358	2.158040
H	4.052743	-1.810443	-1.545565
H	-1.407349	-1.188980	-3.547873
H	-3.015673	-0.700050	-4.113474
H	-1.590753	0.128344	-4.750002
H	-6.199229	-3.456717	1.257635
H	-4.424440	-3.541401	1.448663
H	-5.122217	-2.222858	0.533810
H	0.285913	-5.366156	-0.460575
H	0.672356	-3.833811	-1.255686
H	1.989156	-4.912809	-0.684198
H	2.903987	0.394556	-2.758892
H	3.534280	-0.939426	-3.746989
H	2.229794	0.055945	-4.389355
H	1.662246	-1.895447	3.099328
H	3.214951	-1.423832	3.783131
H	1.808660	-1.656326	4.864110
H	5.516581	1.585848	-0.449954
H	6.919241	1.616992	-1.568654
H	5.438368	0.697750	-1.954301
H	-0.019158	2.482071	-2.158268
H	0.783013	4.044108	-2.278237
H	1.367142	2.704812	-3.280880
H	-1.174198	1.057170	3.644830
H	-2.667835	1.976661	3.850760
H	-2.429211	0.456792	4.763251
H	-3.255585	4.119460	-1.422867
H	-3.327270	5.628899	-2.384550
H	-1.767024	4.898131	-1.915155

48. TPP R = OH

E_{elec} = -1714.091850 a.u.; **H =** -1713.761233 a.u

C	1.889111	-1.843484	-0.342490
C	1.639409	-0.681197	0.405035
C	2.784185	0.019414	0.839937
C	4.088547	-0.365140	0.535241
C	4.283679	-1.517028	-0.222161
C	3.190407	-2.261124	-0.659121
P	0.041698	-0.077457	1.090034
C	-0.247817	1.573488	0.312763
C	-1.303730	2.315012	0.882302
C	-1.616931	3.622953	0.509335
C	-0.845813	4.239532	-0.471403
C	0.202027	3.549273	-1.077157
C	0.481667	2.229587	-0.694154
C	-1.294097	-1.065666	0.305648
C	-2.059171	-1.906400	1.132430
C	-3.176995	-2.607675	0.663118
C	-3.550727	-2.470544	-0.673088
C	-2.814743	-1.662496	-1.535860
C	-1.698960	-0.983846	-1.041024
H	-3.111239	-1.580422	-2.578425
H	-3.743050	-3.252993	1.334200

H	-2.444854	4.159365	0.965060
H	0.795046	4.023385	-1.859626
H	4.944008	0.212286	0.875428
H	3.339332	-3.168716	-1.244281
O	5.583048	-1.873324	-0.506706
O	2.547048	1.147742	1.586314
O	0.824160	-2.598686	-0.767557
O	1.487345	1.549988	-1.328687
O	-1.172630	5.532944	-0.811366
O	-2.051580	1.672856	1.839726
O	-0.937227	-0.217906	-1.877372
O	-4.639428	-3.123532	-1.202314
O	-1.664427	-2.041140	2.439248
H	3.395467	1.514590	1.880919
H	5.572081	-2.690037	-1.030690
H	1.168772	-3.360867	-1.258801
H	-2.301780	-2.612363	2.896690
H	-1.342202	-0.225576	-2.759076
H	-5.061096	-3.643585	-0.499695
H	1.923967	2.157452	-1.946287
H	-2.710132	2.294317	2.188655
H	-0.560564	5.835542	-1.500968

49. TPP R = SO_3H

E_{elec} = -6652.876225 a.u.; **H =** -6652.424997 a.u

C	-1.309048	2.071045	1.327963
C	-0.111429	1.615718	0.706497
C	0.592933	2.599326	-0.042438
C	-0.008134	3.800738	-0.406069
C	-1.328783	4.038695	-0.046355
C	-1.949685	3.233245	0.892852
P	0.145832	-0.195730	0.983501
O	-0.888302	1.181983	3.777391
S	-2.004681	1.431349	2.899528
O	-2.722515	0.061543	2.543553
S	2.319334	2.433069	-0.527168
O	2.626728	3.812601	-1.290867
S	-2.239890	5.325444	-0.885178
O	-3.547309	5.399265	-0.284052
C	1.740135	-0.793850	0.249442
C	2.871306	-0.509801	1.063390
C	4.183884	-0.667335	0.622542
C	4.401446	-1.164026	-0.651633
C	3.339202	-1.647947	-1.405169
C	2.029910	-1.517261	-0.944277
S	2.766140	-0.060256	2.819707
O	1.885853	-1.007300	3.482663
S	0.822709	-2.474889	-1.904853
O	0.130379	-1.656838	-2.869602
S	6.060546	-1.279622	-1.299789
O	6.187842	0.114770	-2.114410
C	-1.274768	-1.020661	0.132449
C	-2.075697	-0.572350	-0.939697
C	-3.287026	-1.190602	-1.272075
C	-3.680759	-2.329319	-0.591456

C	-2.897387	-2.851063	0.433353
C	-1.732531	-2.189142	0.800785
S	-1.672650	0.747389	-2.103018
O	-2.676525	1.782418	-1.981301
S	-5.208800	-3.142230	-1.043245
O	-5.674764	-3.893915	0.097386
S	-1.005274	-2.922742	2.300620
O	-1.393752	-2.054507	3.409454
O	-1.996176	0.028685	-3.493582
O	-0.267008	1.057276	-2.063523
O	-6.016944	-2.193080	-1.755442
O	-4.688704	-4.199346	-2.159475
O	0.563380	-2.875478	2.139271
O	-1.391672	-4.305902	2.308772
O	1.772515	-3.402157	-2.818726
O	0.113880	-3.342841	-0.997600
O	6.981739	-1.240408	-0.189821
O	6.070789	-2.308224	-2.303326
O	4.113189	0.095692	3.299654
O	2.004650	1.328106	2.844857
O	-1.401818	6.480519	-1.066372
O	-2.330405	4.657778	-2.348585
O	3.100259	2.533191	0.696482
O	2.559040	1.357086	-1.453985
O	-3.040750	2.368002	3.248065
H	3.545262	-2.135831	-2.349170
H	5.011465	-0.416369	1.276046
H	-3.903583	-0.799222	-2.074145
H	-3.207301	-3.743944	0.964436
H	0.516386	4.530998	-1.007748
H	-2.918650	3.502000	1.298366
H	0.967988	-2.142100	2.709828
H	-4.556063	-5.061709	-1.720196
H	-1.277289	-0.634556	-3.635689
H	2.496941	1.962898	2.252354
H	6.580470	0.781936	-1.518280
H	1.984759	-4.215880	-2.319301
H	-2.212536	-0.698959	2.969261
H	2.522459	3.656887	-2.251220
H	-2.746961	3.765768	-2.288349

50. H+

E_{elec} = 0.000000 a.u.; **H =** 5/2 RT a.u

H	0.000000.	0.000000.	0.000000

51. TPP protonated. R = CF_3

E_{elec} = -4072.018895 a.u.; **H =** -4071.653958 a.u

C	0.971227	-2.402525	-0.888964
C	1.151096	-1.405370	0.099001
C	2.269925	-1.530522	0.968744
C	3.187723	-2.564149	0.798548
C	3.016986	-3.502670	-0.209597
C	1.903228	-3.431812	-1.031682
P	-0.006712	-0.003132	0.434827
C	-1.797345	-0.303899	0.083045

C	-2.473120	-1.211892	0.944948
C	-3.825193	-1.490910	0.760420
C	-4.542547	-0.873397	-0.254425
C	-3.916900	0.057448	-1.068961
C	-2.561225	0.350319	-0.912693
C	-1.835634	-1.889341	2.156748
F	-0.645962	-2.481675	1.851042
C	-6.022445	-1.171943	-0.423429
F	-6.754076	-0.391893	0.407853
C	-2.058139	1.474976	-1.812567
F	-2.857071	1.664057	-2.875595
C	2.528914	-0.631440	2.176327
F	1.609970	-0.860428	3.152080
C	4.058216	-4.589835	-0.413909
F	5.066293	-4.126899	-1.191221
C	-0.251172	-2.538787	-1.791698
F	-0.011496	-3.336122	-2.845750
C	0.631721	1.697485	0.087138
C	1.590117	2.030820	-0.899911
C	2.014744	3.351099	-1.053702
C	1.513036	4.359405	-0.245001
C	0.611040	4.047786	0.762095
C	0.175108	2.737149	0.943704
C	2.323211	1.031459	-1.789796
F	1.517317	0.062580	-2.282831
C	-0.742071	2.524230	2.146835
F	-0.092016	1.850116	3.132958
C	1.998569	5.788772	-0.415329
F	3.078871	6.011842	0.370833
F	2.357081	6.035311	-1.693164
F	1.040995	6.673955	-0.064868
F	-1.848098	1.792238	1.829886
F	-1.175717	3.679111	2.667703
F	3.309411	0.413455	-1.082110
F	2.903869	1.628361	-2.843676
F	-2.024826	2.647329	-1.119605
F	-0.811609	1.258594	-2.292313
F	-6.294280	-2.461357	-0.127006
F	-6.431816	-0.931770	-1.686714
F	-1.583947	-0.981227	3.137108
F	-2.620262	-2.839664	2.680654
F	-1.285661	-3.088667	-1.096729
F	-0.685136	-1.356011	-2.285504
F	4.590523	-4.981173	0.764258
F	3.529100	-5.673050	-1.020265
F	3.740777	-0.831137	2.709845
F	2.448450	0.692680	1.859987
H	1.748340	-4.187560	-1.791791
H	4.035437	-2.640714	1.468461
H	-4.318438	-2.197633	1.416618
H	-4.483881	0.561271	-1.842107
H	0.239380	4.828922	1.413922
H	2.741956	3.589534	-1.820196
H	-0.012673	0.001436	1.825656

52. TPP protonated. R = CH_3

E_{elec} = -1391.186183 a.u.; **H =** -1390.629015 a.u

C	-3.432212	2.975447	-0.250372
C	-3.501770	2.009738	0.758629
C	-2.465630	1.106728	0.992706
C	-1.308772	1.177371	0.171052
C	-1.212948	2.136338	-0.866213
C	-2.283824	3.013610	-1.046683
P	0.001964	0.002665	0.555482
C	-0.363303	-1.718336	0.167683
C	0.273698	-2.687150	0.988781
C	0.003528	-4.034908	0.755886
C	-0.871523	-4.454502	-0.251116
C	-1.474790	-3.477096	-1.047751
C	-1.244055	-2.111866	-0.868622
C	1.232414	-2.320022	2.098807
C	-1.940314	-1.147630	-1.800394
C	-1.166781	-5.916554	-0.459499
C	-2.622109	0.094227	2.104779
C	-0.029679	2.261930	-1.797016
C	-4.555931	3.955729	-0.461375
C	1.674468	0.547583	0.165064
C	2.453550	-0.016646	-0.873889
C	3.752416	0.463333	-1.051843
C	4.300212	1.470310	-0.251845
C	3.500980	2.017503	0.756801
C	2.197815	1.579914	0.988808
C	1.962925	-1.096045	-1.810212
C	1.402346	2.224219	2.101532
C	5.715276	1.942578	-0.458940
H	4.353591	0.039672	-1.853144
H	3.900590	2.814911	1.379402
H	0.496870	-4.778938	1.377283
H	-2.143993	-3.786612	-1.847373
H	-4.392366	1.951893	1.380402
H	-2.220478	3.747199	-1.847168
H	-2.401754	-0.926783	1.772361
H	-1.958888	0.320938	2.950324
H	-3.645889	0.107044	2.484847
H	-5.525216	3.507377	-0.224859
H	-4.426501	4.824665	0.196335
H	-4.579158	4.321457	-1.491506
H	0.289452	1.296235	-2.201013
H	-0.288902	2.905180	-2.641097
H	0.837050	2.700418	-1.291309
H	-2.753593	-0.614490	-1.296907
H	-1.260331	-0.389979	-2.201865
H	-2.366835	-1.692260	-2.645912
H	-1.985242	-6.233346	0.199397
H	-1.473884	-6.119445	-1.489053
H	-0.297075	-6.536256	-0.222718
H	2.006192	-1.618759	1.765735
H	0.707871	-1.860286	2.947268
H	1.733114	-3.214703	2.475122
H	0.401316	2.529875	1.776401

H	1.283556	1.542935	2.954940
H	1.919224	3.113789	2.468121
H	6.398119	1.384641	0.194364
H	6.042282	1.785016	-1.490326
H	5.821091	3.003370	-0.214078
H	1.907497	-2.069775	-1.312364
H	0.966152	-0.881891	-2.208196
H	2.645592	-1.189326	-2.657991
H	0.005176	0.001423	1.953796

53. TPP protonated. R = CN

E_{elec} = -1867.659396 a.u.; **H =** -1867.362656 a.u

C	2.367484	0.660660	-0.779310
C	1.434642	0.998894	0.223939
C	1.627449	2.185976	0.971609
C	2.731297	3.008363	0.735440
C	3.655900	2.655287	-0.258025
C	3.471581	1.488634	-1.013634
P	-0.000141	0.000150	0.679143
C	-1.582738	0.743133	0.224364
C	-2.707311	0.315071	0.971021
C	-3.971652	0.859355	0.734886
C	-4.128318	1.837728	-0.257490
C	-3.025662	2.263123	-1.011875
C	-1.756341	1.721412	-0.777560
C	-2.528449	-0.715457	1.944694
N	-2.294703	-1.576558	2.690708
C	-5.417914	2.398548	-0.506210
N	-6.466837	2.855201	-0.707020
C	-0.672053	2.164523	-1.595186
N	0.205793	2.528536	-2.263210
C	0.646269	2.546135	1.946083
N	-0.215849	2.774367	2.692636
C	4.786713	3.491243	-0.506805
N	5.706926	4.170902	-0.707716
C	2.207095	-0.498638	-1.598351
N	2.081223	-1.439810	-2.267482
C	0.147744	-1.741931	0.224381
C	1.081976	-2.501245	0.970119
C	1.243132	-3.868312	0.734032
C	0.473219	-4.493690	-0.257235
C	-0.447700	-3.752004	-1.010754
C	-0.613479	-2.381899	-0.776625
C	1.886083	-1.830608	1.942577
N	2.515930	-1.197510	2.687659
C	-1.540419	-1.664846	-1.593455
N	-2.295220	-1.086509	-2.260679
C	0.632614	-5.890916	-0.505824
N	0.761827	-7.027622	-0.706571
H	-1.031358	-4.235408	-1.786502
H	1.963165	-4.436724	1.312472
H	-4.823784	0.521126	1.314203
H	-3.152474	3.009464	-1.788416
H	2.865408	3.914835	1.315487
H	4.180441	1.225808	-1.791168

H	0.000111	0.000439	2.079683

54. TPP protonated. R = 5F

E_{elec} = -2526.317889 a.u.; **H =** -2526.128893 a.u

C	-0.922083	-2.557658	-1.176510
C	-0.210455	-1.706956	-0.313059
C	0.319937	-2.250927	0.867299
C	0.164071	-3.597893	1.172355
C	-0.541540	-4.424058	0.288718
C	-1.089456	-3.906927	-0.891827
P	0.000775	0.000433	-0.785006
C	-1.372688	1.036438	-0.314150
C	-1.762123	2.070273	-1.183022
C	-2.848987	2.887246	-0.898555
C	-3.563997	2.676550	0.287063
C	-3.192424	1.660295	1.176237
C	-2.102013	0.854327	0.871275
F	-1.066661	2.274482	-2.313545
F	-3.207892	3.858092	-1.732118
F	-4.598605	3.449589	0.573061
F	-3.875272	1.478396	2.302191
F	-1.742583	-0.103202	1.736133
F	0.981180	-1.465439	1.727166
F	0.671034	-4.101490	2.293313
F	-0.696942	-5.706143	0.574963
F	-1.760965	-4.699744	-1.720456
F	-1.452772	-2.054531	-2.302988
C	1.585103	0.671927	-0.315513
C	1.791268	1.406580	0.862590
C	3.035865	1.945899	1.165574
C	4.103723	1.745781	0.281865
C	3.929334	1.009605	-0.896735
C	2.677088	0.478768	-1.179036
F	0.780355	1.589092	1.721973
F	2.505800	-0.235538	-2.303346
F	4.951094	0.822733	-1.725757
F	5.291735	2.253377	0.566051
F	3.218803	2.640179	2.284349
H	-0.000136	-0.000784	-2.181244

55. TPP protonated. R = F

E_{elec} = -1930.698276 a.u.; **H =** -1930.466839 a.u

C	-1.332455	1.866493	-0.927434
C	-1.324036	1.096642	0.248772
C	-2.434370	1.241479	1.101139
C	-3.484709	2.098673	0.838984
C	-3.413066	2.835022	-0.343116
C	-2.354061	2.740658	-1.245330
P	-0.000075	0.000054	0.715143
C	1.611458	0.598651	0.248649
C	2.293336	1.485992	1.101810
C	3.560997	1.966540	0.839395
C	4.161703	1.537587	-0.343777
C	3.549133	0.669455	-1.246823
C	2.281091	0.222429	-0.928786

F	1.663518	1.878935	2.228436
F	5.386163	1.986176	-0.629727
F	1.651756	-0.604257	-1.783038
F	-2.460560	0.497570	2.226438
F	-4.414253	3.670630	-0.628956
F	-0.300832	1.736893	-1.780683
C	-0.287471	-1.694743	0.248174
C	-0.948868	-2.086846	-0.928711
C	-1.195472	-3.408603	-1.246544
C	-0.748821	-4.372984	-0.343897
C	-0.075909	-4.067050	0.838631
C	0.141468	-2.728882	1.100817
F	-1.351346	-1.128789	-1.782660
F	0.798190	-2.379717	2.226536
F	-0.972451	-5.657767	-0.629632
H	-2.335066	3.321423	-2.159718
H	-4.324429	2.190747	1.517317
H	4.061631	2.646262	1.518439
H	4.041709	0.363608	-2.162014
H	0.263447	-4.840347	1.517305
H	-1.707329	-3.682419	-2.161301
H	-0.000217	-0.000228	2.110687

56. TPP protonated. R = H

E_{elec} = -1037.125751 a.u.; **H =** -1036.828452 a.u

C	2.220005	0.559773	0.723849
C	1.699386	-0.218429	-0.320920
C	2.486151	-1.203383	-0.942910
C	3.794653	-1.402670	-0.513930
C	4.314726	-0.629635	0.528549
C	3.531427	0.347069	1.145457
P	0.001544	-0.000240	-0.843150
C	-1.037334	-1.361086	-0.321745
C	-2.281909	-1.551658	-0.946773
C	-3.109956	-2.584162	-0.517479
C	-2.704011	-3.417856	0.528856
C	-1.468254	-3.226044	1.148892
C	-0.626980	-2.198125	0.726681
C	-0.659194	1.579192	-0.321805
C	-0.196697	2.754089	-0.939537
C	-0.680560	3.986130	-0.510681
C	-1.614685	4.048119	0.527784
C	-2.071593	2.880204	1.140580
C	-1.598110	1.639019	0.718942
H	-2.799802	2.933459	1.943828
H	-0.332647	4.897042	-0.987686
H	-4.069458	-2.741154	-1.000252
H	-1.155788	-3.878492	1.958222
H	4.409998	-2.156988	-0.994227
H	3.941092	0.947160	1.951944
H	5.337382	-0.788084	0.857521
H	2.082100	-1.807354	-1.751560
H	1.609851	1.324887	1.194920
H	0.339121	-2.051005	1.200769
H	-3.353651	-4.223208	0.858266

H	-2.600588	-0.902057	-1.758180
H	-1.957674	0.727157	1.186878
H	-1.990267	5.012361	0.856903
H	0.532413	2.707467	-1.744591
H	0.002366	-0.000878	-2.248377

57. TPP protonated. R = NH_2
E_{elec} = -1535.678005 a.u.; **H =** -1535.220818 a.u

C	-0.253339	2.650644	1.068289
C	0.236763	1.669661	0.153867
C	0.950773	2.108094	-0.998096
C	1.267642	3.450958	-1.156399
C	0.862324	4.399667	-0.196838
C	0.084962	3.990689	0.901080
P	-0.019808	-0.042806	0.534240
C	1.362986	-1.076884	0.110300
C	2.551974	-0.903760	0.894572
C	3.613534	-1.793107	0.774019
C	3.543009	-2.865084	-0.134820
C	2.407112	-3.016424	-0.948954
C	1.326787	-2.140776	-0.849090
N	2.602309	0.125901	1.824924
N	4.568875	-3.772043	-0.209600
N	0.271041	-2.264831	-1.738960
N	1.228294	1.192888	-2.020568
N	1.238622	5.712707	-0.327434
N	-1.024866	2.249023	2.155435
C	-1.610819	-0.681310	0.086898
C	-2.174977	-1.674642	0.953438
C	-3.518879	-2.009202	0.861061
C	-4.334796	-1.397001	-0.112558
C	-3.776347	-0.485884	-1.023241
C	-2.426672	-0.129928	-0.956541
N	-5.651566	-1.764107	-0.225974
N	-1.366703	-2.244273	1.933212
N	-1.916279	0.764626	-1.865497
H	1.827706	3.771963	-2.031338
H	-0.291075	4.730543	1.603535
H	4.508898	-1.646040	1.372918
H	2.366198	-3.828048	-1.671439
H	-3.932248	-2.774133	1.513466
H	-4.395832	-0.067643	-1.812977
H	-6.090717	-2.257879	0.537726
H	-6.270649	-1.203220	-0.793570
H	-0.920947	0.784754	-2.079901
H	-2.512070	1.011567	-2.643839
H	-1.797262	-2.983261	2.476226
H	-0.415364	-2.475057	1.661749
H	3.488407	0.241983	2.301514
H	2.186251	1.012680	1.553192
H	4.620302	-4.391275	-1.005998
H	5.453833	-3.548218	0.222807
H	0.220519	-3.147905	-2.232445
H	-0.635365	-1.930598	-1.422078
H	1.845667	1.547882	-2.743470

H	1.520493	0.267675	-1.706820
H	0.775519	6.411200	0.236873
H	1.573842	6.037730	-1.223439
H	-1.383305	3.004178	2.728192
H	-1.739554	1.553559	1.964811
H	-0.026458	-0.053842	1.940025

58. TPP protonated. R = NO_2

E_{elec} = -2878.650305 a.u.; **H =** -2878.315689 a.u

C	0.028778	-2.517436	-0.624769
C	0.687493	-1.633033	0.248249
C	1.853735	-2.162736	0.843738
C	2.351112	-3.432429	0.575494
C	1.649551	-4.230840	-0.307484
C	0.475010	-3.801833	-0.903482
P	-0.002109	0.001106	0.793475
C	-1.761685	0.222702	0.247329
C	-2.804587	-0.521735	0.841779
C	-4.152581	-0.316282	0.573285
C	-4.491922	0.691332	-0.309237
C	-3.532013	1.493214	-0.904614
C	-2.196942	1.236062	-0.625632
N	-2.504090	-1.601057	1.812027
O	-1.292114	-1.835729	2.001547
N	-5.938017	0.933314	-0.620646
O	-6.745741	0.206872	-0.052965
N	-1.246303	2.142800	-1.323270
O	-0.449276	1.628555	-2.099993
N	2.637507	-1.362216	1.814256
O	2.234123	-0.195586	2.004049
N	2.164731	-5.603694	-0.618450
O	1.504017	-6.264119	-1.413247
N	-1.233077	-2.149259	-1.320967
O	-1.191722	-1.193772	-2.088103
C	1.070645	1.412137	0.245164
C	2.163330	1.280753	-0.630689
C	3.056300	2.306235	-0.909138
C	2.847203	3.537705	-0.310300
C	1.806936	3.749305	0.574287
C	0.952082	2.686849	0.842005
N	2.468086	0.004676	-1.331739
O	1.619184	-0.427185	-2.103664
N	-0.131845	2.968045	1.813005
O	-0.944474	2.038342	2.000380
N	3.783424	4.666500	-0.620304
O	3.563187	5.728458	-0.049230
O	4.684081	4.421528	-1.416063
O	-0.179124	4.051719	2.356175
O	3.558711	-0.501162	-1.074804
O	-1.352844	3.338480	-1.058282
O	-6.178309	1.836719	-1.414709
O	-3.417825	-2.187744	2.352872
O	-2.211228	-2.847839	-1.063433
O	3.197278	-5.939099	-0.049469
O	3.603161	-1.859562	2.354490

H	-0.076537	-4.454063	-1.572563
H	3.261824	-3.782034	1.049184
H	-4.911403	-0.929745	1.046425
H	-3.820134	2.297338	-1.573707
H	1.658739	4.712820	1.049343
H	3.894869	2.152469	-1.580296
H	-0.001989	0.002012	2.171765

59. TPP protonated. R = OCH_3

E_{elec} = -2068.476993 a.u.; **H =** -2067.868633 a.u

C	-1.391192	1.779745	-1.043859
C	-1.409726	1.010771	0.144139
C	-2.588511	1.004528	0.918038
C	-3.702927	1.767271	0.561482
C	-3.636841	2.537837	-0.606706
C	-2.490289	2.545094	-1.414808
P	-0.014843	0.066961	0.728143
C	1.567757	0.791843	0.346819
C	2.019241	1.864121	1.154664
C	3.267089	2.440393	0.958662
C	4.083745	1.953117	-0.072443
C	3.660823	0.913037	-0.910475
C	2.399731	0.347784	-0.700101
O	1.137595	2.272773	2.109759
C	1.529543	3.342261	2.989349
O	5.284555	2.571904	-0.189116
C	6.186093	2.133895	-1.220073
O	1.889713	-0.637256	-1.481458
C	2.701747	-1.185459	-2.528457
O	-2.554193	0.198643	2.017004
C	-3.700191	0.163107	2.884736
O	-4.654099	3.315167	-1.053402
C	-5.866675	3.369498	-0.281929
O	-0.251921	1.699560	-1.775426
C	-0.138878	2.497966	-2.962498
C	-0.121436	-1.678811	0.385253
C	-0.750097	-2.211570	-0.757659
C	-0.861356	-3.592497	-0.944579
C	-0.314976	-4.450601	0.018849
C	0.345180	-3.953209	1.152136
C	0.443097	-2.578520	1.322206
O	-1.214834	-1.302535	-1.650972
C	-1.922510	-1.762726	-2.810228
O	1.081023	-1.988661	2.371544
C	1.660181	-2.833312	3.382652
O	-0.365138	-5.803206	-0.063713
C	-1.026184	-6.398650	-1.193228
H	-2.498555	3.148840	-2.313243
H	-4.596857	1.753152	1.168680
H	3.633509	3.257304	1.566845
H	4.292987	0.556834	-1.711527
H	0.764899	-4.658662	1.857609
H	-1.354162	-3.987575	-1.821680
H	2.096255	-2.148930	4.109967
H	0.890450	-3.449716	3.860223

H	2.441297	-3.469425	2.951890
H	-0.947153	-7.473927	-1.035940
H	-2.081656	-6.103841	-1.226899
H	-0.525318	-6.122113	-2.128703
H	-2.221096	-0.856344	-3.337166
H	-1.271362	-2.369279	-3.451153
H	-2.811937	-2.336664	-2.525399
H	0.683027	3.483494	3.660947
H	2.421876	3.065820	3.561897
H	1.713632	4.260411	2.420677
H	2.092739	-1.970878	-2.976441
H	2.938344	-0.423164	-3.280535
H	3.625113	-1.616677	-2.124693
H	7.070284	2.761260	-1.111389
H	6.457030	1.080914	-1.079087
H	5.744785	2.282357	-2.212858
H	-3.429898	-0.525004	3.685370
H	-3.905857	1.156812	3.298639
H	-4.578658	-0.215127	2.349758
H	0.859864	2.291865	-3.347833
H	-0.893354	2.206046	-3.702578
H	-0.235599	3.564038	-2.727806
H	-6.519578	4.052662	-0.824328
H	-6.333763	2.379436	-0.219664
H	-5.671191	3.760698	0.723404
H	-0.103694	0.148379	2.115460

60. TPP protonated. R = $OCOCH_3$

E_{elec} = -3089.144666 a.u.; **H =** -3088.443524 a.u

C	-1.885735	1.654825	1.266346
C	-0.578193	1.444707	0.786341
C	0.192111	2.570057	0.445535
C	-0.322783	3.853019	0.578081
C	-1.644790	4.018921	1.002295
C	-2.435024	2.923278	1.359910
P	-0.032433	-0.262511	0.724135
C	-1.102869	-1.323480	-0.246076
C	-1.202901	-2.650741	0.207583
C	-2.213946	-3.496471	-0.215638
C	-3.153564	-2.993548	-1.120604
C	-3.047038	-1.701961	-1.645941
C	-2.008777	-0.878862	-1.227400
O	-0.287854	-3.041995	1.177368
C	0.873559	-3.786278	0.850879
O	1.714844	-3.858838	1.701461
O	-4.179549	-3.785873	-1.563362
C	-5.309786	-4.084247	-0.737435
O	-6.098615	-4.864612	-1.174364
O	-1.859489	0.431546	-1.619429
C	-2.026319	0.971476	-2.908947
O	-1.886617	2.164996	-2.976621
O	1.510050	2.361321	0.083970
C	2.054257	2.714255	-1.154536
O	3.203057	2.399128	-1.331378
O	-2.169597	5.275330	1.154047

C	-2.539086	6.082574	0.037415
O	-2.886127	7.199387	0.272183
O	-2.639859	0.522978	1.517992
C	-2.964784	0.066669	2.831174
O	-3.599743	-0.946116	2.882023
C	1.716574	-0.492983	0.438382
C	2.289641	-0.958091	-0.758310
C	3.649089	-1.235914	-0.827682
C	4.456579	-0.959910	0.281689
C	3.923954	-0.451850	1.470503
C	2.557122	-0.244255	1.541590
O	1.407481	-1.220059	-1.788986
C	1.551164	-0.855752	-3.143279
O	0.638171	-1.174213	-3.855439
O	1.929153	0.327164	2.631232
C	1.769333	-0.311012	3.900365
O	0.985615	0.216708	4.636268
O	5.791893	-1.258846	0.253958
C	6.730291	-0.531088	-0.539416
O	7.837925	-0.970025	-0.595702
C	0.911072	-4.408690	-0.515300
C	-5.377242	-3.349843	0.575302
C	-2.318744	0.054666	-4.060905
C	2.564838	-1.555331	4.149910
C	6.213697	0.725770	-1.187932
C	2.782051	-0.109569	-3.574243
C	1.171502	3.418529	-2.146840
C	-2.439866	0.888726	3.971634
C	-2.469987	5.415862	-1.312163
H	-3.457669	3.069164	1.689100
H	0.296585	4.719636	0.378144
H	-2.289584	-4.510155	0.160867
H	-3.801052	-1.358251	-2.341818
H	4.579264	-0.221340	2.301596
H	4.087735	-1.677734	-1.713562
H	-1.864931	-0.929112	-3.938502
H	-3.403825	-0.056241	-4.166415
H	-1.940571	0.535058	-4.963781
H	-6.363837	-3.516242	1.006153
H	-4.615766	-3.725815	1.266060
H	-5.192086	-2.279330	0.446771
H	0.149682	-5.191771	-0.590222
H	0.699072	-3.665565	-1.289861
H	1.897350	-4.846682	-0.664030
H	3.163569	0.563819	-2.804748
H	3.564964	-0.831900	-3.831188
H	2.529465	0.443499	-4.479749
H	2.412295	-2.285059	3.347714
H	3.632816	-1.321936	4.198713
H	2.244335	-1.973487	5.103574
H	5.562431	1.297472	-0.522265
H	7.071629	1.322861	-1.495943
H	5.626686	0.471401	-2.076633
H	0.143100	3.048660	-2.134727
H	1.152811	4.489342	-1.915874

H	1.612861	3.295710	-3.136213
H	-1.351963	1.003984	3.903298
H	-2.880172	1.890485	3.958000
H	-2.698442	0.386937	4.903112
H	-2.896945	4.409390	-1.294135
H	-3.000534	6.043798	-2.027119
H	-1.426841	5.319841	-1.630339
H	-0.234267	-0.747368	2.016506

61. TPP protonated. R = OH

E_{elec} = -1714.529290 a.u.; **H =** -1714.185837 a.u

C	-2.216093	-0.759986	-0.929527
C	-1.407425	-0.997759	0.203617
C	-1.763827	-2.066159	1.053413
C	-2.891570	-2.845679	0.816053
C	-3.682205	-2.563358	-0.304797
C	-3.347005	-1.527466	-1.181367
P	0.002029	-0.004061	0.648504
C	-0.156494	1.714276	0.208653
C	-0.942618	2.541260	1.043670
C	-1.070804	3.904026	0.816049
C	-0.409940	4.467777	-0.279798
C	0.359294	3.678880	-1.143162
C	0.475130	2.311641	-0.900498
O	-1.572491	1.914911	2.082039
O	-0.567985	5.804793	-0.455898
O	1.191163	1.497408	-1.723743
O	-0.932204	-2.294436	2.113762
O	-4.798043	-3.274258	-0.610633
O	-1.833831	0.245649	-1.763046
C	1.571011	-0.725759	0.213165
C	1.775759	-1.565895	-0.899757
C	3.017503	-2.150797	-1.139969
C	4.081786	-1.886051	-0.270000
C	3.920964	-1.037265	0.829592
C	2.676899	-0.465046	1.054603
O	0.715394	-1.771186	-1.728145
O	2.446648	0.389475	2.095503
O	5.318423	-2.419535	-0.443358
H	-3.973207	-1.341904	-2.048879
H	-3.146030	-3.664603	1.485129
H	-1.673907	4.535372	1.461193
H	0.857698	4.124349	-2.001600
H	4.766796	-0.835985	1.479668
H	3.156662	-2.800819	-2.001278
H	3.245664	0.470223	2.642039
H	5.332012	-2.993733	-1.227134
H	0.965316	-2.388345	-2.434985
H	-1.286517	-3.006886	2.670507
H	-2.482342	0.335807	-2.480558
H	-4.951732	-3.965343	0.054939
H	1.604634	2.024861	-2.426455
H	-2.049087	2.566480	2.622363
H	-0.072940	6.103870	-1.236718
H	-0.002245	-0.004840	2.042717

62. TPP protonated. R = SO_3H

E_{elec} = -6653.222295 a.u.; **H =** -6652.760653 a.u

C	-1.923535	-2.150908	0.893919
C	-1.405940	-1.093446	0.112455
C	-2.100047	-0.770293	-1.063695
C	-3.306912	-1.370939	-1.413417
C	-3.806370	-2.377554	-0.598573
C	-3.107901	-2.789799	0.533750
P	0.062203	-0.146213	0.665421
C	1.721554	-0.856297	0.270983
C	2.782231	-0.514468	1.152129
C	4.120686	-0.704818	0.812285
C	4.433708	-1.240478	-0.427097
C	3.429017	-1.745753	-1.241132
C	2.088208	-1.602410	-0.883651
S	2.561024	0.051324	2.862612
O	1.769678	1.410769	2.701223
S	6.151726	-1.423284	-0.927190
O	6.178617	-2.446464	-1.932332
S	0.994137	-2.620334	-1.927798
O	-0.094996	-3.108045	-1.120560
S	-1.352758	0.334764	-2.239020
O	0.051829	0.355481	-1.822370
S	-5.380024	-3.147475	-1.011806
O	-4.883966	-4.341952	-1.976261
S	-1.227717	-2.769011	2.439971
O	-1.661938	-4.123291	2.599109
C	-0.196981	1.681224	0.497311
C	-1.377035	2.182751	1.105363
C	-1.940006	3.390342	0.676084
C	-1.294890	4.147182	-0.285962
C	-0.008999	3.808329	-0.698333
C	0.536519	2.587345	-0.320595
S	-2.102636	1.605290	2.691194
O	-3.079284	2.593912	3.044749
S	2.306920	2.394894	-0.677944
O	2.628026	1.265296	-1.509033
S	-2.038717	5.660562	-0.907309
O	-2.507054	5.178115	-2.372205
O	-0.987672	1.283789	3.549519
O	-2.863662	0.258432	2.319540
O	2.656311	3.730475	-1.477181
O	2.957994	2.559016	0.611764
O	-3.200823	5.940925	-0.105175
O	-0.966490	6.587265	-1.131419
O	1.622430	-0.859275	3.500444
O	3.859229	0.259632	3.430601
O	0.763193	-1.998294	-3.213324
O	1.961138	-3.841508	-2.284757
O	6.384931	-0.018241	-1.684151
O	6.957197	-1.442446	0.267210
O	-2.107112	1.546194	-2.374072
O	-1.437437	-0.448048	-3.600404
O	-5.916843	-3.722009	0.195590

O	-6.081587	-2.211163	-1.841159
O	-1.523464	-1.762791	3.455260
O	0.335573	-2.752537	2.160306
H	3.703135	-2.284281	-2.141708
H	4.899970	-0.438897	1.518792
H	-3.834393	-1.077991	-2.316236
H	-3.489576	-3.600903	1.145965
H	0.555349	4.510555	-1.298369
H	-2.859900	3.743133	1.130953
H	0.808508	-2.131941	2.802455
H	-4.963907	-5.189337	-1.494448
H	-0.668626	-1.092856	-3.631921
H	2.303132	2.031746	2.124584
H	6.944211	0.551278	-1.118743
H	1.928719	-4.512279	-1.571898
H	-2.471980	-0.476661	2.883138
H	2.734897	3.523403	-2.431219
H	-3.442726	4.896062	-2.336061
H	-0.041523	-0.271518	2.051835

3. FLP cartesian coordinates

63. FLP$_1$: Acid R = NH$_2$. Base R = CN

E_{elec} = -3086.105920 a.u.; **H =** -3085.370007 a.u

C	2.686509	2.633130	0.511700
C	2.808733	1.511851	-0.346041
C	3.264874	1.750389	-1.660873
C	3.503781	3.054109	-2.113653
C	3.324220	4.146838	-1.258318
C	2.943984	3.932675	0.071392
P	2.080915	-0.026654	0.341823
C	1.858887	-1.193441	-1.046910
C	2.454484	-2.467871	-1.212785
C	2.064913	-3.302520	-2.273373
C	1.091054	-2.889592	-3.182248
C	0.473794	-1.638227	-3.031864
C	0.864978	-0.804238	-1.977326
C	3.496172	-2.966128	-0.374796
N	4.345212	-3.435221	0.267887
C	0.691894	-3.748655	-4.249433
N	0.327212	-4.450969	-5.101950
C	0.313802	0.511072	-1.929996
N	-0.053236	1.614104	-1.964986
C	3.558154	0.687775	-2.571186
N	3.837001	-0.139098	-3.338502
C	3.541476	5.477198	-1.732149
N	3.716189	6.561504	-2.111179
C	2.310693	2.424676	1.876479
N	2.037099	2.249664	2.993898
C	3.336506	-0.720643	1.487181
C	2.810780	-1.570351	2.492151
C	3.615951	-2.107114	3.499829
C	4.975139	-1.777419	3.549658
C	5.518065	-0.924390	2.583077
C	4.711910	-0.406520	1.560451

C	1.427013	-1.938370	2.480624
N	0.320997	-2.294328	2.514824
C	5.372799	0.404481	0.585696
N	5.977592	1.046866	-0.170567
C	5.804890	-2.309280	4.584759
N	6.476985	-2.739252	5.429244
H	-0.318224	1.027774	2.141964
N	-1.228875	0.866785	1.725834
C	-1.623908	1.835281	0.795529
C	-2.750264	1.587922	-0.026089
C	-3.129492	2.620408	-0.913866
C	-2.430412	3.826892	-0.999984
C	-1.350847	4.060776	-0.140081
C	-0.967246	3.072911	0.772482
N	-4.227988	2.370602	-1.768998
B	-3.596007	0.248889	0.070208
C	-2.891980	-1.071610	-0.482888
C	-2.547272	-2.139682	0.397741
C	-1.969985	-3.332275	-0.065841
C	-1.849057	-3.555847	-1.440290
C	-2.197872	-2.534201	-2.339362
C	-2.661380	-1.288730	-1.865529
N	-0.602959	5.246623	-0.231320
N	-2.847345	-2.041218	1.749057
N	-1.445077	-4.793118	-1.920011
N	-2.878174	-0.287113	-2.802149
C	-4.995490	0.209062	0.703914
C	-5.472953	1.230304	1.626496
C	-6.619082	1.044255	2.406438
C	-7.394117	-0.112497	2.279269
C	-7.034960	-1.086450	1.340556
C	-5.889579	-0.931551	0.558912
N	-4.825053	2.435461	1.768995
N	-8.555091	-0.258362	3.022008
N	-5.678653	-1.867669	-0.429278
H	-6.921768	1.822771	3.104625
H	-7.673194	-1.955612	1.192311
H	-1.715100	-4.117349	0.641851
H	-2.156802	-2.723566	-3.410338
H	-0.143301	3.257227	1.456249
H	-2.731611	4.585536	-1.719940
H	3.839327	3.210639	-3.132605
H	2.834425	4.768534	0.752363
H	2.546591	-4.266674	-2.392383
H	-0.243958	-1.274102	-3.754173
H	6.572904	-0.674528	2.608956
H	3.184753	-2.769934	4.241399
H	-1.315182	-0.091969	1.400384
H	-1.082254	6.008865	-0.698321
H	-0.210147	5.555795	0.651436
H	-4.602868	3.206918	-2.205731
H	-4.969676	1.850759	-1.297691
H	-5.021965	2.958093	2.611674
H	-3.878172	2.515466	1.412042
H	-8.910284	-1.200316	3.124461

H	-8.623565	0.292744	3.867839
H	-6.137211	-2.760566	-0.303126
H	-4.739839	-1.928895	-0.810230
H	-3.108021	-0.591381	-3.739253
H	-3.356082	0.570912	-2.526071
H	-1.169503	-4.867964	-2.889752
H	-0.943371	-5.401533	-1.288465
H	-2.232934	-2.531452	2.386923
H	-3.174863	-1.144075	2.088840

64. FLP$_2$: Acid R = OCH$_3$. Base R = CF$_3$
E$_{elec}$ = -5823.212835 a.u.; **H =** -5822.259834 a.u

C	1.679046	-0.556419	2.637294
C	2.553350	-0.763671	1.527939
C	3.560983	-1.748999	1.697347
C	3.689781	-2.450381	2.899899
C	2.855805	-2.185999	3.972522
C	1.850659	-1.237231	3.837867
P	2.014228	0.113038	-0.016622
B	-4.213102	-0.303147	-0.033976
C	-4.011638	-1.799743	-0.463173
C	-4.633879	-2.347570	-1.614222
C	-4.402487	-3.646264	-2.066403
C	-3.520955	-4.460875	-1.351726
C	-2.901229	-3.999931	-0.191085
C	-3.161612	-2.688113	0.230609
C	4.542574	-2.209852	0.626171
F	5.030344	-1.223008	-0.149100
C	2.981310	-2.958969	5.262085
F	4.192908	-3.551314	5.389965
C	0.446039	0.350397	2.587203
F	-0.494207	-0.129149	1.757020
C	2.648253	-0.718626	-1.551195
C	3.615653	-0.265900	-2.486007
C	3.830797	-0.946058	-3.688237
C	3.123152	-2.095944	-3.993820
C	2.153902	-2.555358	-3.112028
C	1.897664	-1.872709	-1.927360
C	3.345521	-2.816679	-5.300807
F	4.554217	-2.531903	-5.843095
C	4.455448	0.999194	-2.352065
F	4.919971	1.234468	-1.110046
C	0.711382	-2.410810	-1.122898
F	-0.333857	-1.565969	-1.146650
C	2.440859	1.919348	0.022482
C	1.581146	2.724260	-0.783318
C	1.672747	4.112118	-0.787619
C	2.577267	4.759794	0.043536
C	3.389516	4.012293	0.879449
C	3.339438	2.615262	0.872320
C	0.445392	2.153923	-1.636420
F	0.869379	1.194900	-2.509527
C	2.677156	6.265139	0.013825

F	1.459350	6.844826	-0.145475
C	4.266821	1.976023	1.899298
F	4.855276	0.835617	1.491870
C	-4.382421	0.823893	-1.119524
C	-3.616679	0.867184	-2.308259
C	-3.718656	1.895982	-3.245010
C	-4.629437	2.929283	-3.015244
C	-5.431414	2.937809	-1.873245
C	-5.289831	1.887791	-0.953200
C	-4.202149	0.101483	1.486887
C	-4.850435	-0.669983	2.479578
C	-4.809715	-0.358177	3.838517
C	-4.095688	0.771531	4.247227
C	-3.453621	1.589239	3.318247
C	-3.525393	1.241223	1.960578
F	2.799072	-2.159372	6.343859
F	2.043535	-3.944472	5.339084
F	-0.130572	0.468786	3.812789
F	0.736808	1.626088	2.197995
F	5.624020	-2.823199	1.174843
F	3.967953	-3.131114	-0.201827
F	5.277863	2.815595	2.245578
F	3.596775	1.696625	3.055719
F	3.220468	6.763799	1.151337
F	3.454160	6.688959	-1.019904
F	0.273195	-3.597109	-1.621594
F	1.020818	-2.657306	0.183204
F	-0.530651	1.623037	-0.879341
F	-0.130868	3.115436	-2.403730
F	5.548578	0.958414	-3.158092
F	3.745609	2.100080	-2.736301
F	3.271459	-4.163409	-5.147770
F	2.402639	-2.472087	-6.221121
H	-5.317015	-0.948373	4.591529
H	-2.885291	2.452063	3.636689
H	-4.888671	-4.056114	-2.942810
H	-2.204953	-4.623531	0.352156
H	-3.105314	1.937219	-4.136507
H	-6.159163	3.720760	-1.710873
H	4.464489	-3.199569	2.999415
H	1.181830	-1.040684	4.665974
H	1.012653	4.691246	-1.420519
H	4.077599	4.517338	1.544933
H	4.574140	-0.577675	-4.383259
H	1.580585	-3.440431	-3.356135
O	-4.081748	0.999320	5.602665
O	-2.875466	1.987869	1.009341
O	-2.721076	-0.157967	-2.472581
O	-6.081504	1.817758	0.166315
O	-2.539326	-2.180629	1.340423
O	-5.521957	-1.522152	-2.249660
O	-3.325970	-5.715813	-1.873041
O	-4.668709	3.904158	-3.983319
O	-5.559807	-1.745304	2.010062
C	-6.914444	2.926203	0.488565

H	-7.360538	2.680334	1.453836
H	-6.331152	3.853186	0.577632
H	-7.709921	3.064639	-0.257224
C	-2.065459	-0.289578	-3.730387
H	-1.508412	-1.225663	-3.667043
H	-2.792131	-0.345441	-4.551973
H	-1.368970	0.538864	-3.913317
C	-5.570504	4.993894	-3.786501
H	-5.432542	5.647353	-4.649559
H	-6.612168	4.647620	-3.749835
H	-5.335075	5.544007	-2.865498
C	-2.328916	3.248673	1.378778
H	-1.982200	3.694847	0.444967
H	-3.090219	3.895464	1.835889
H	-1.479482	3.138030	2.066312
C	-3.349925	2.132623	6.071965
H	-3.461258	2.123725	7.157529
H	-2.286335	2.055148	5.808710
H	-3.760047	3.067584	5.667189
C	-6.139831	-2.639925	2.954738
H	-6.579514	-3.442577	2.360127
H	-5.379633	-3.054013	3.631314
H	-6.924043	-2.147656	3.546107
C	-6.061758	-1.935349	-3.502013
H	-6.650467	-1.086971	-3.855024
H	-5.264749	-2.158817	-4.223975
H	-6.711179	-2.814349	-3.390341
C	-1.851750	-3.067676	2.215851
H	-1.590500	-2.462800	3.086164
H	-2.497078	-3.899954	2.527273
H	-0.935507	-3.463152	1.757526
C	-2.419810	-6.581936	-1.186539
H	-2.406316	-7.507491	-1.764375
H	-1.410013	-6.151823	-1.152314
H	-2.765146	-6.790072	-0.165157

65. FLP_3. Acid R = NH_2. Base R = H

E_{elec} = -2255.510801 a.u.; **H =** -2254.774756 a.u

C	4.919398	-1.610318	-2.045421
C	4.582100	-1.207094	-0.743283
C	5.410961	-1.594571	0.319793
C	6.552108	-2.362047	0.083587
C	6.885274	-2.745788	-1.216575
C	6.067242	-2.365415	-2.282497
P	3.057274	-0.198246	-0.546368
C	2.734656	-0.332063	1.260336
C	2.922114	0.723489	2.164092
C	2.588939	0.569136	3.512475
C	2.073514	-0.642227	3.976962
C	1.890799	-1.701967	3.084247
C	2.210472	-1.546370	1.737217
H	3.322184	1.670030	1.813079
H	1.808087	-0.757139	5.024309
H	2.034562	-2.369270	1.051108
H	5.160313	-1.295757	1.333678

H	7.775075	-3.342725	-1.398097
H	4.276188	-1.326628	-2.875858
C	3.680751	1.521814	-0.710119
C	2.714039	2.537833	-0.798825
C	3.096881	3.873987	-0.909413
C	4.452086	4.208270	-0.963412
C	5.419448	3.203955	-0.896576
C	5.038361	1.867805	-0.763099
H	1.656570	2.286956	-0.757346
H	5.797322	1.093005	-0.700121
H	4.753561	5.248007	-1.062097
C	-0.671693	3.782664	0.369266
C	-1.660150	3.679618	-0.614836
C	-2.334745	2.467338	-0.808427
C	-2.030010	1.301501	-0.037827
C	-1.001688	1.446268	0.948735
C	-0.345276	2.669516	1.150708
B	-2.795304	-0.057975	-0.262632
C	-4.356659	-0.086551	-0.360299
C	-5.044935	-1.029655	-1.202024
C	-6.434577	-1.177293	-1.172518
C	-7.207275	-0.397364	-0.304415
C	-6.589587	0.562169	0.504400
C	-5.200324	0.743174	0.461111
N	-4.330424	-1.764670	-2.135265
N	-8.583100	-0.608358	-0.212472
N	-4.668520	1.757557	1.234882
N	-3.255860	2.383629	-1.847395
N	0.041780	4.979498	0.519936
N	-0.709495	0.400462	1.811947
C	-1.951477	-1.388968	-0.318770
C	-2.317873	-2.559417	0.427032
C	-1.441091	-3.645385	0.568434
C	-0.209579	-3.649941	-0.093818
C	0.147984	-2.570460	-0.905321
C	-0.689170	-1.450308	-0.997717
N	-3.560559	-2.657040	1.032669
N	-0.288820	-0.421169	-1.837550
N	0.696592	-4.698733	0.107609
H	0.721492	-0.317064	-1.910007
H	-6.916371	-1.897003	-1.831853
H	-7.192445	1.186158	1.162186
H	-1.892637	4.535903	-1.245446
H	0.422597	2.746511	1.917838
H	1.086742	-2.584018	-1.454365
H	-1.734642	-4.498330	1.178348
H	7.184454	-2.657105	0.917143
H	6.317735	-2.664895	-3.296842
H	2.733483	1.398959	4.199739
H	1.479804	-2.646686	3.429314
H	6.475178	3.459835	-0.941368
H	2.329749	4.642137	-0.946559
H	-0.762600	0.463909	-1.678915
H	-3.633485	-3.275668	1.828763
H	-4.117006	-1.810956	1.088638

H	0.277162	-5.577319	0.390205
H	1.366933	-4.827851	-0.642095
H	-9.120104	0.177151	0.135248
H	-9.010601	-1.035611	-1.025423
H	-5.205081	2.052668	2.038686
H	-3.659932	1.788875	1.339430
H	-4.754994	-2.641275	-2.412159
H	-3.334467	-1.846670	-1.942044
H	-3.919533	1.616742	-1.764007
H	-3.693359	3.259440	-2.105846
H	-0.462258	5.810747	0.232382
H	0.476791	5.100741	1.427464
H	-0.914535	-0.527290	1.453174
H	0.188194	0.454811	2.278653

66. FLP_4: Acid R = OCH_3. Base R = H

E_{elec} = -2788.295829 a.u.; **H =** -2787.409925 a.u

C	-3.422694	-2.746597	3.678023
C	-2.271587	-2.424033	4.397951
C	-1.354400	-1.503074	3.886500
C	-1.569415	-0.899577	2.639396
C	-2.725209	-1.242004	1.917461
C	-3.648620	-2.147478	2.435845
P	-0.396484	0.268551	1.831071
C	1.046851	0.194295	2.972899
C	1.941737	-0.874615	2.795414
C	3.084865	-0.988036	3.583551
C	3.368802	-0.019681	4.550635
C	2.494349	1.053439	4.726809
C	1.338333	1.157198	3.949322
B	0.270873	-0.272902	-1.491008
C	1.382522	-1.339170	-1.142530
C	1.158595	-2.459572	-0.303032
C	2.157291	-3.375305	0.035671
C	3.441327	-3.200869	-0.483310
C	3.722708	-2.144363	-1.347563
C	2.689539	-1.249578	-1.666251
C	0.670586	1.242182	-1.695109
C	0.084060	2.040363	-2.709385
C	0.368362	3.395277	-2.885967
C	1.296567	4.003867	-2.041010
C	1.950273	3.268272	-1.052935
C	1.632108	1.907884	-0.906003
C	-1.216847	-0.736640	-1.743291
C	-1.520640	-1.978654	-2.356681
C	-2.821078	-2.448167	-2.545534
C	-3.890410	-1.651349	-2.136310
C	-3.668364	-0.396439	-1.569952
C	-2.344082	0.036848	-1.392538
C	-1.134701	1.898391	2.270309
C	-2.233360	2.067409	3.124692
C	-2.767345	3.337858	3.353145
C	-2.204547	4.459547	2.742872
C	-1.106450	4.302567	1.892744
C	-0.585343	3.033420	1.650184

H	-0.089350	3.991098	-3.665654
H	2.672370	3.737268	-0.399635
H	-3.035438	-3.399528	-3.016024
H	-4.495888	0.222775	-1.253968
H	1.980177	-4.210011	0.701940
H	4.704639	-2.030879	-1.785525
H	-3.622603	3.450117	4.015191
H	-0.668306	5.166075	1.397906
H	3.762480	-1.823739	3.426977
H	2.706962	1.811443	5.476915
H	-2.086582	-2.886458	5.364507
H	-4.533271	-2.402115	1.857067
O	1.510999	5.342792	-2.263591
O	2.229980	1.162626	0.077600
O	-0.103847	-2.579601	0.214385
O	2.901111	-0.238305	-2.567546
O	-2.088584	1.247693	-0.803024
O	-0.442727	-2.695412	-2.802498
O	-5.139182	-2.186669	-2.339562
O	4.368138	-4.134626	-0.087040
O	-0.771227	1.384635	-3.554384
H	-2.621388	5.447105	2.922818
H	0.236779	2.918154	0.950627
H	-2.676619	1.202404	3.609879
H	0.662203	1.993955	4.100082
H	4.266357	-0.099191	5.158529
H	-2.888402	-0.818251	0.931374
H	1.755365	-1.608355	2.017419
H	-0.464153	-1.256570	4.458130
H	-4.134852	-3.463708	4.077988
C	4.233256	0.060837	-2.969868
H	4.154656	0.968065	-3.571618
H	4.879690	0.248606	-2.101591
H	4.663697	-0.745882	-3.579812
C	5.702381	-3.987685	-0.575999
H	6.268801	-4.807600	-0.131350
H	5.736317	-4.067951	-1.670766
H	6.135701	-3.027557	-0.265432
C	-0.415430	-3.736635	0.992191
H	-1.475258	-3.645953	1.229142
H	-0.231954	-4.656248	0.421162
H	0.162987	-3.758594	1.925044
C	-0.647025	-4.033336	-3.247731
H	0.352701	-4.429728	-3.433647
H	-1.146955	-4.636160	-2.477805
H	-1.234376	-4.064469	-4.175645
C	-6.264938	-1.407782	-1.931519
H	-7.141284	-2.011320	-2.173391
H	-6.240288	-1.208996	-0.851403
H	-6.310444	-0.456124	-2.477597
C	-3.181876	2.120741	-0.520418
H	-2.727643	3.033558	-0.135696
H	-3.757865	2.340429	-1.429374
H	-3.844051	1.698469	0.247102
C	-1.536618	2.148557	-4.481534

H	-2.208654	1.432473	-4.957758
H	-2.123044	2.923600	-3.969706
H	-0.897955	2.616810	-5.243035
C	3.277445	1.750475	0.848101
H	3.631542	0.955280	1.504007
H	4.094863	2.100249	0.202957
H	2.906451	2.582205	1.461568
C	2.447658	6.010004	-1.416533
H	2.459031	7.047924	-1.753242
H	2.133702	5.966537	-0.364683
H	3.452941	5.579233	-1.516003

67. FLP_5: Acid R = OCH_3. Base R = OCH_3

E_{elec} = -3819.582917 a.u.; **H =** 3818.387941 a.u

C	-2.909239	2.851481	-3.776472
C	-3.565909	1.621279	-3.749810
C	-3.399347	0.773230	-2.649705
C	-2.542084	1.099946	-1.576811
C	-1.923347	2.361419	-1.643388
C	-2.093087	3.247639	-2.715910
P	-2.011208	0.034398	-0.169041
C	-2.802713	-1.605811	-0.451086
C	-2.196311	-2.408464	-1.444486
C	-2.572040	-3.728124	-1.688659
C	-3.588868	-4.290234	-0.912621
C	-4.237361	-3.539416	0.065821
C	-3.853360	-2.200644	0.266751
B	3.239155	-0.030615	0.211404
C	3.352506	-0.689726	-1.206977
C	2.651727	-0.200765	-2.337498
C	2.674685	-0.845247	-3.576211
C	3.420371	-2.017429	-3.718277
C	4.154139	-2.537093	-2.651604
C	4.109156	-1.856373	-1.425859
C	3.146519	-0.943272	1.491914
C	3.854380	-0.668297	2.679771
C	3.712773	-1.409276	3.850150
C	2.831029	-2.489254	3.861827
C	2.123323	-2.838620	2.708421
C	2.292536	-2.066447	1.549212
C	3.229953	1.528404	0.386467
C	4.051148	2.378504	-0.392508
C	4.069186	3.766216	-0.251860
C	3.248533	4.351216	0.715574
C	2.421465	3.572790	1.524317
C	2.418403	2.180725	1.339321
C	-2.827858	0.738802	1.326910
C	-3.745614	1.809572	1.396600
C	-4.132730	2.377351	2.615108
C	-3.644992	1.843645	3.807773
C	-2.786198	0.743447	3.798334
C	-2.394842	0.217474	2.559320
H	4.289415	-1.155355	4.732455
H	1.453365	-3.687695	2.705910
H	4.711365	4.407850	-0.842078

H	1.788757	4.028733	2.273002
H	2.129860	-0.475061	-4.435748
H	4.760032	-3.424793	-2.770408
H	-4.826290	3.207490	2.667773
H	-2.427412	0.306313	4.719694
H	-2.108101	-4.332783	-2.457713
H	-5.046486	-3.964010	0.643986
H	-4.214175	1.365741	-4.578792
H	-1.600932	4.210230	-2.716480
O	2.727833	-3.169321	5.054448
O	1.599070	-2.346674	0.403173
O	1.913286	0.928977	-2.136040
O	4.857276	-2.280565	-0.355397
O	1.581235	1.377878	2.055273
O	4.880876	1.739589	-1.277937
O	3.321464	5.720927	0.796615
O	3.371011	-2.594269	-4.965085
O	4.730061	0.402671	2.760188
O	-4.083420	2.461547	4.955815
O	-1.569156	-0.874399	2.469371
O	-4.265179	2.246751	0.212417
O	-4.518876	-1.405630	1.158096
O	-3.892679	-5.600559	-1.200997
O	-1.148832	2.679798	-0.559300
O	-1.217960	-1.781128	-2.168303
O	-4.083865	-0.404262	-2.551402
O	-3.140499	3.621491	-4.892440
C	5.439103	-3.580237	-0.395417
H	5.849565	-3.743048	0.602781
H	4.683429	-4.346470	-0.613520
H	6.248038	-3.638802	-1.137274
C	4.105226	-3.803710	-5.162419
H	3.919794	-4.092762	-6.198237
H	5.181358	-3.645093	-5.011322
H	3.753741	-4.596634	-4.488790
C	1.087456	1.398414	-3.200688
H	0.608903	2.299430	-2.821564
H	1.681207	1.642743	-4.092208
H	0.311159	0.665751	-3.452209
C	5.631576	2.527995	-2.197579
H	6.133742	1.810422	-2.848761
H	4.974858	3.173728	-2.795471
H	6.380167	3.144618	-1.681247
C	2.528182	6.366334	1.794390
H	2.744810	7.431315	1.696054
H	1.456606	6.191968	1.626385
H	2.800645	6.024763	2.801489
C	0.693014	1.972836	2.997565
H	0.152687	1.141779	3.446363
H	1.241608	2.520705	3.775742
H	-0.028836	2.634750	2.502395
C	5.845840	0.368681	1.858059
H	6.242954	1.385249	1.822863
H	6.609878	-0.326264	2.235472
H	5.551075	0.060428	0.847952

C	0.607025	-3.369520	0.432757
H	0.203766	-3.410004	-0.576710
H	1.039732	-4.345593	0.694602
H	-0.204147	-3.115315	1.125551
C	1.881575	-4.317450	5.087476
H	1.960232	-4.710893	6.102477
H	0.835855	-4.052047	4.877982
H	2.213214	-5.079783	4.369590
C	-0.483044	3.941927	-0.542849
H	0.094897	3.953792	0.379825
H	0.208792	4.044964	-1.388753
H	-1.207004	4.768343	-0.551297
C	-4.842009	-0.855967	-3.670267
H	-5.212778	-1.841057	-3.382032
H	-5.687897	-0.187136	-3.879767
H	-4.213955	-0.944503	-4.566811
C	-2.478290	4.884242	-4.967780
H	-2.782116	5.317878	-5.921952
H	-2.787317	5.546774	-4.147753
H	-1.386729	4.763283	-4.948408
C	-5.483016	-1.989964	2.027019
H	-5.794731	-1.180480	2.689385
H	-6.353040	-2.366959	1.471372
H	-5.045273	-2.804669	2.620242
C	-4.938005	-6.214748	-0.447168
H	-5.010649	-7.235907	-0.825125
H	-4.697856	-6.234504	0.624564
H	-5.895226	-5.697330	-0.597849
C	-0.580179	-2.509050	-3.220107
H	0.182660	-1.841018	-3.617206
H	-0.093098	-3.417409	-2.842140
H	-1.302530	-2.777244	-4.002234
C	-5.086087	3.411285	0.207131
H	-5.305021	3.598418	-0.845461
H	-6.022301	3.246149	0.757587
H	-4.556844	4.273432	0.634851
C	-3.601430	1.954001	6.200416
H	-4.051278	2.585536	6.968259
H	-3.911964	0.911412	6.353682
H	-2.506849	2.022052	6.261340
C	-1.138802	-1.502451	3.675982
H	-0.493970	-2.321876	3.361976
H	-0.552794	-0.819373	4.304573
H	-1.995803	-1.887238	4.245566

68. FLP$_6$: Acid R = NH$_2$. Base R = NH$_2$

E$_{elec}$ = -2754.035335 a.u.; **H =** -2753.143558 a.u

C	-3.198522	2.517624	-0.287691
C	-2.748541	1.375743	0.451217
C	-1.493809	1.523667	1.132130
C	-0.698749	2.671258	0.980334
C	-1.124881	3.710951	0.148485
C	-2.380660	3.644415	-0.467418
B	-3.429147	-0.040796	0.291484
C	-4.977255	-0.224284	0.375808

C	-5.782883	0.573333	1.263523
C	-7.180105	0.540075	1.231547
C	-7.843779	-0.285197	0.316403
C	-7.106539	-1.107774	-0.541778
C	-5.705508	-1.106552	-0.499971
N	-5.168624	1.346262	2.236026
N	-9.235977	-0.251780	0.227382
N	-5.042163	-1.994024	-1.325267
N	-1.067307	0.559639	2.019424
H	-0.067021	0.552454	2.217596
N	-0.263485	4.780005	-0.128254
N	-4.463016	2.536909	-0.861213
C	-2.465903	-1.241546	-0.050963
C	-2.569134	-2.508461	0.603881
C	-1.687916	-3.564275	0.333632
C	-0.660961	-3.396548	-0.608503
C	-0.541379	-2.183476	-1.292513
C	-1.420333	-1.124787	-1.026172
N	0.298981	-4.386069	-0.792919
N	-3.504056	-2.674463	1.626850
N	-1.322267	0.000145	-1.828981
N	1.024948	-2.162462	1.783686
C	2.377701	-2.403779	1.574644
C	3.149234	-1.513927	0.764260
C	4.559015	-1.731482	0.696268
C	5.145804	-2.804522	1.382527
C	4.363098	-3.676214	2.148149
C	2.982113	-3.475577	2.241556
P	2.253502	-0.074118	0.080955
C	2.319376	-0.025759	-1.754194
C	2.664272	-1.101499	-2.619297
C	2.584812	-0.954890	-4.012250
C	2.109001	0.225647	-4.588862
C	1.674176	1.263964	-3.759179
C	1.754378	1.142032	-2.364047
N	5.357766	-0.962147	-0.125959
N	4.971055	-4.704859	2.873619
N	2.999271	-2.365194	-2.129012
N	1.971485	0.328476	-5.978520
N	1.350508	2.206789	-1.575593
C	3.194926	1.361781	0.738702
C	4.078656	2.226522	0.047220
C	4.517876	3.424311	0.625565
C	4.125880	3.778836	1.918947
C	3.314038	2.904734	2.650658
C	2.860384	1.713897	2.074512
N	4.611801	1.851171	-1.194675
N	4.499462	5.018389	2.460138
N	2.012119	0.884590	2.824839
H	-7.752205	1.153812	1.925052
H	-7.623094	-1.765674	-1.238836
H	-1.770224	-4.495661	0.891968
H	0.234279	-2.062265	-2.043107
H	0.244736	2.747536	1.516782
H	-2.727916	4.469539	-1.087286

H	5.173036	4.082763	0.057135
H	3.039326	3.144209	3.676679
H	2.848197	-1.799895	-4.645758
H	1.250393	2.169403	-4.189103
H	6.221185	-2.960143	1.313575
H	2.373312	-4.138068	2.852917
H	2.136023	-0.116258	2.691053
H	1.979922	1.117484	3.811415
H	4.508801	5.046047	3.473759
H	5.356693	5.401108	2.076645
H	5.041197	2.619004	-1.699165
H	3.965814	1.333621	-1.791685
H	0.577146	-1.610761	1.052527
H	0.472534	-2.979844	2.016975
H	4.361800	-5.479088	3.110011
H	5.860785	-5.021546	2.506660
H	6.351307	-1.008790	0.057639
H	5.050597	-0.033074	-0.407378
H	0.782228	2.914096	-2.026089
H	0.995870	1.983982	-0.646226
H	1.964052	1.276801	-6.335549
H	2.594836	-0.266754	-6.511422
H	3.661351	-2.871227	-2.705759
H	3.243273	-2.394492	-1.141297
H	-1.480445	-0.359707	1.908178
H	-4.578748	3.127522	-1.674324
H	-4.948936	1.646837	-0.913719
H	-0.721006	5.656718	-0.350114
H	0.493668	4.900868	0.535744
H	-9.664298	-1.080937	-0.166893
H	-9.714360	0.066600	1.061699
H	-5.533228	-2.310319	-2.149966
H	-4.038454	-1.876678	-1.425066
H	-5.703017	2.145726	2.553536
H	-4.191699	1.566252	2.047181
H	-4.281744	-2.018578	1.588881
H	-3.810401	-3.629724	1.768604
H	0.000316	-5.344481	-0.668380
H	0.933796	-4.242134	-1.570085
H	-1.665996	0.863503	-1.420888
H	-0.441266	0.106867	-2.319651

69. FLP$_7$: Acid R = H. Base R = CN

E$_{elec}$ = -2587.580995 a.u.; **H =** -2587.003743 a.u

C	0.463107	2.382025	1.161509
C	1.671421	1.691833	0.884178
C	2.877957	2.336025	1.238969
C	2.870167	3.614051	1.816460
C	1.663367	4.272430	2.071514
C	0.452110	3.644933	1.755523
P	1.455217	0.154942	-0.093572
C	3.125275	-0.385792	-0.627334
C	3.889389	-1.468248	-0.137565
C	5.073618	-1.860378	-0.776265
C	5.534037	-1.171561	-1.903262

C	4.808278	-0.079724	-2.394618
C	3.616123	0.293480	-1.770554
C	3.545846	-2.182276	1.052841
C	6.745764	-1.575269	-2.544736
C	2.906383	1.429119	-2.277647
C	4.162307	1.724653	1.090727
C	1.666132	5.573667	2.662414
C	-0.789345	1.742673	0.899596
C	0.816967	-1.140890	1.034787
C	0.587783	-1.051674	2.425368
C	-0.096410	-2.067338	3.107800
C	-0.545515	-3.201997	2.426552
C	-0.300432	-3.332469	1.054522
C	0.359208	-2.309467	0.373050
C	1.086186	0.025313	3.222309
C	-1.247515	-4.229992	3.127845
C	0.632301	-2.480101	-1.021584
C	-3.223791	-3.840896	-1.001751
C	-3.522737	-4.108658	0.336424
C	-3.894159	-3.062135	1.185878
C	-3.990262	-1.763689	0.689199
C	-3.702646	-1.462475	-0.659672
C	-3.301633	-2.533959	-1.485484
B	-3.879091	-0.005690	-1.223250
C	-5.026540	0.898438	-0.659858
C	-4.878178	2.298288	-0.557663
C	-5.892477	3.104443	-0.045989
C	-7.102991	2.532902	0.354072
C	-7.284677	1.152239	0.253298
C	-6.254062	0.349030	-0.232701
C	-2.952427	0.517232	-2.369007
C	-1.630057	0.046816	-2.505306
C	-0.777611	0.521151	-3.497109
C	-1.236690	1.476989	-4.406080
C	-2.547704	1.950139	-4.312250
C	-3.387638	1.482471	-3.302746
H	-3.939423	2.751298	-0.866365
H	-5.745534	4.178546	0.037381
H	-7.900344	3.161029	0.743364
H	-8.226482	0.703835	0.559532
H	-6.404062	-0.726109	-0.299096
H	-4.289964	-0.957335	1.353860
H	-4.118558	-3.267973	2.229308
H	-3.467265	-5.124554	0.720124
H	-2.937486	-4.650540	-1.668887
H	-3.071670	-2.339927	-2.530300
H	-1.264069	-0.692428	-1.800401
H	0.239206	0.145521	-3.565031
H	-0.574862	1.852018	-5.182375
H	-2.910791	2.687349	-5.023983
H	-4.405783	1.858727	-3.239458
H	5.643673	-2.691996	-0.377235
H	5.163158	0.472467	-3.257486
H	-0.258846	-1.974022	4.175804
H	-0.636587	-4.209831	0.516531

H	3.811387	4.082643	2.081769
H	-0.492245	4.133069	1.968473
N	7.729098	-1.903825	-3.068863
N	3.331463	-2.791193	2.019149
N	2.366831	2.377841	-2.678804
N	0.900885	-2.636559	-2.142178
N	1.481813	0.866352	3.920132
N	-1.824546	-5.064692	3.693376
N	-1.809668	1.205793	0.753072
N	1.666501	6.632872	3.139643
N	5.235391	1.285469	1.011668

70. FLP$_8$: Acid R = H. Base R = CF$_3$

E$_{elec}$ = -4791.904367 a.u.; **H =** -4791.259475 a.u

C	1.629180	-0.781381	4.550372
C	2.421622	-1.455993	3.637064
C	2.280628	-1.240502	2.263323
C	1.309288	-0.336060	1.759554
C	0.461165	0.281295	2.726911
C	0.642022	0.080113	4.091582
P	0.800496	0.006245	0.005824
C	1.368401	-1.331837	-1.150559
C	0.543829	-2.496506	-1.119413
C	0.765457	-3.570889	-1.974584
C	1.770180	-3.512920	-2.931476
C	2.543910	-2.369941	-3.036807
C	2.361803	-1.293694	-2.163144
B	-4.887222	-0.019067	-0.086036
C	-4.890857	-1.523126	0.350011
C	-4.261977	-1.942128	1.542582
C	-4.212840	-3.285106	1.909487
C	-4.823036	-4.250363	1.105601
C	-5.472345	-3.864997	-0.069366
C	-5.491543	-2.523127	-0.444990
C	-4.835340	0.356225	-1.605468
C	-5.422830	1.543389	-2.093979
C	-5.355304	1.891753	-3.441598
C	-4.670852	1.070312	-4.340085
C	-4.073206	-0.107436	-3.886109
C	-4.168965	-0.463020	-2.542722
C	-4.902326	1.109113	0.999993
C	-5.558228	0.932337	2.237464
C	-5.557525	1.926913	3.213734
C	-4.872286	3.122951	2.989261
C	-4.205410	3.323634	1.778690
C	-4.235228	2.336632	0.796242
C	1.345889	1.690515	-0.558353
C	2.305916	2.569583	0.009276
C	2.474103	3.867151	-0.480559
C	1.722399	4.330835	-1.547540
C	0.749606	3.511706	-2.102888
C	0.540561	2.228545	-1.605677
H	-5.828887	2.805301	-3.792558
H	-3.533309	-0.745854	-4.581121

H	-6.083559	1.769616	4.152124
H	-3.663169	4.249637	1.603833
H	-3.700284	-3.581986	2.821216
H	-5.955804	-4.612458	-0.693548
H	3.217047	4.513899	-0.031996
H	0.137854	3.877948	-2.917080
H	0.130870	-4.445641	-1.913115
H	3.306383	-2.311560	-3.802456
H	3.174410	-2.146979	3.993549
H	-0.002383	0.586145	4.798880
H	-4.603531	1.346852	-5.389350
H	-3.695127	-1.378505	-2.199996
H	-3.780398	-1.200255	2.173525
H	-5.991187	-2.235254	-1.367003
H	-3.706543	2.500380	-0.138473
H	-6.086074	0.000531	2.426604
H	-4.857381	3.894815	3.754745
H	-4.792869	-5.298323	1.393392
H	-5.950528	2.194852	-1.401408
C	1.906179	5.739914	-2.055935
C	-0.658179	1.496105	-2.213223
C	3.186360	2.257949	1.213527
C	3.266687	-0.106488	-2.470016
C	2.011921	-4.699115	-3.832718
C	-0.752804	1.138413	2.361796
C	-0.689017	-2.636296	-0.223299
C	3.201233	-2.110729	1.415993
C	1.779283	-1.026674	6.031934
F	4.341568	-0.476177	-3.213844
F	2.604565	0.833198	-3.206871
F	3.764226	0.512837	-1.382348
F	2.708644	-4.363652	-4.945429
F	2.721175	-5.667779	-3.191762
F	0.845932	-5.266972	-4.235576
F	-1.660806	-1.769595	-0.586512
F	-1.226303	-3.878776	-0.305502
F	-0.423490	-2.434844	1.095947
F	-1.173645	2.174226	-3.268569
F	-0.352274	0.256455	-2.683945
F	-1.658181	1.361372	-1.313883
F	4.235873	3.115486	1.302176
F	2.486981	2.397826	2.377541
F	3.718532	1.020256	1.210335
F	1.655997	5.831646	-3.385767
F	1.058609	6.604641	-1.432315
F	3.165101	6.195977	-1.842750
F	4.244353	-2.590520	2.141387
F	2.536309	-3.205283	0.943500
F	3.745274	-1.482196	0.355867
F	1.587203	0.109726	6.748497
F	0.866243	-1.933564	6.477630
F	3.005768	-1.508443	6.347941
F	-1.710905	0.400197	1.758369
F	-1.322649	1.690128	3.461687
F	-0.449880	2.180068	1.539719

71. FLP$_9$: Acid R = H. Base R = H

E_{elec} = -1756.997177 a.u.; **H =** -1756.419746 a.u

B	-0.003502	-0.001099	-1.357344
C	-0.017430	1.579317	-1.736179
C	-1.052668	2.151765	-2.497869
C	1.006660	2.458255	-1.320480
C	-1.087981	3.515896	-2.797796
C	0.978211	3.823271	-1.604668
C	-0.079084	4.363367	-2.340496
H	-1.908479	3.916714	-3.388851
H	1.782369	4.465316	-1.252202
C	-1.365834	-0.802606	-1.736047
C	-1.345502	-1.980188	-2.505756
C	-2.638296	-0.357891	-1.315215
C	-2.509822	-2.690633	-2.808548
C	-3.806811	-1.062925	-1.602187
C	-3.747368	-2.243578	-2.346175
H	-2.447768	-3.597568	-3.405909
H	-4.764281	-0.689606	-1.245799
C	1.371475	-0.780138	-1.737263
C	1.618722	-2.107373	-1.323285
C	2.385416	-0.170693	-2.498837
C	2.813914	-2.766830	-1.608727
C	3.583298	-0.823719	-2.800071
C	3.811010	-2.121989	-2.344272
H	2.966297	-3.784991	-1.257653
H	4.341100	-0.314035	-3.390986
P	0.000775	-0.000037	0.754903
C	-1.378340	0.947993	1.493410
C	1.515188	0.722291	1.483362
C	-0.127492	-1.669173	1.492068
C	-2.245531	0.373333	2.433068
C	-1.595714	2.276155	1.083609
C	2.770953	0.236029	1.076141
C	1.458030	1.772425	2.410250
C	0.804217	-2.132355	2.431489
C	-1.169055	-2.521663	1.082826
C	-3.317360	1.105830	2.943628
C	-2.665993	3.002546	1.600819
C	3.938942	0.801579	1.582657
C	2.632065	2.336069	2.910203
C	0.706508	-3.426882	2.942018
C	-1.262202	-3.811866	1.599882
C	-3.532735	2.419401	2.527249
H	-3.983914	0.645420	3.667841
H	-2.823791	4.025522	1.270736
C	3.874203	1.855780	2.495914
H	4.901259	0.418697	1.254528
H	2.572004	3.152721	3.624507
C	-0.323189	-4.270575	2.525879
H	1.438781	-3.773643	3.666093
H	-2.069092	-4.460368	1.270181
H	-0.108448	5.427038	-2.563459
H	1.845526	2.067552	-0.751216

H	0.860099	-2.638292	-0.754899
H	2.236984	0.840947	-2.865845
H	-2.718526	0.560457	-0.740207
H	-0.395651	-2.353555	-2.877978
H	-4.654335	-2.799066	-2.571597
H	4.745833	-2.629739	-2.568468
H	-1.853468	1.516431	-2.865641
H	-4.370695	2.986811	2.923102
H	-0.935307	2.737755	0.357576
H	-2.089802	-0.648538	2.762955
H	0.497786	2.156242	2.738641
H	4.787507	2.299121	2.883403
H	-1.899537	-2.180403	0.357319
H	2.835226	-0.576217	0.360240
H	1.610841	-1.485988	2.761455
H	-0.395059	-5.280025	2.921706

72. FLP_{10}: Acid R = H. Base R = OCH_3

E_{elec} = -2788.284807 a.u.; **H =** -2787.398279 a.u

B	-0.475775	-0.253662	1.976514
C	-2.072581	0.071074	2.141239
C	-2.547192	1.048991	3.037190
C	-3.053894	-0.767555	1.572626
C	-3.906971	1.204958	3.321445
C	-4.414893	-0.606328	1.826231
C	-4.853767	0.386377	2.705468
H	-4.223888	1.966742	4.031063
H	-5.132484	-1.270593	1.349142
C	0.461729	0.794370	2.767265
C	1.398947	0.428661	3.748207
C	0.324073	2.178074	2.529809
C	2.127110	1.374361	4.474433
C	1.059205	3.134012	3.229717
C	1.957946	2.736537	4.223002
H	2.831120	1.045785	5.236594
H	0.925794	4.190313	3.005507
C	-0.367204	-1.833191	2.325925
C	-0.399269	-2.882775	1.390217
C	-0.428342	-2.216281	3.681532
C	-0.441437	-4.224464	1.771959
C	-0.462189	-3.554865	4.078228
C	-0.461715	-4.572756	3.123569
H	-0.457728	-5.000208	1.008255
H	-0.502957	-3.801551	5.137201
P	0.133034	0.018197	-0.190069
C	0.838742	1.640619	-0.729552
C	-1.436624	-0.235457	-1.098666
C	1.416247	-1.094704	-0.894394
C	2.046331	1.999746	-0.095175
C	0.375605	2.532668	-1.729147
C	-1.831438	-1.398471	-1.795180
C	-2.424695	0.751054	-0.914225
C	1.589843	-1.154036	-2.298355
C	2.304686	-1.858244	-0.115814
C	2.685486	3.228903	-0.296499

O	2.560773	1.041654	0.714169
C	0.987500	3.770436	-1.938825
O	-0.661286	2.126153	-2.513479
C	-3.117586	-1.540674	-2.316994
O	-0.902262	-2.393614	-1.907562
C	-3.714946	0.641327	-1.440943
O	-2.035041	1.826457	-0.175718
C	2.505119	-2.010902	-2.901959
O	0.788789	-0.319491	-3.012199
C	3.233973	-2.734935	-0.701568
O	2.250860	-1.694355	1.230852
C	2.121905	4.122407	-1.204453
H	3.587249	3.466724	0.249450
C	3.778094	1.289324	1.419765
H	0.625023	4.465289	-2.685632
C	-1.285318	3.072721	-3.377593
C	-4.051389	-0.517995	-2.136616
H	-3.426922	-2.434875	-2.842684
C	-1.280766	-3.632630	-2.507652
H	-4.443663	1.417346	-1.256657
C	-3.017746	2.792985	0.207136
C	3.313224	-2.813084	-2.090338
H	2.617520	-2.073636	-3.977041
C	0.778294	-0.414850	-4.434139
H	3.893019	-3.315788	-0.071872
C	2.836904	-2.697795	2.068483
O	2.633208	5.365105	-1.472370
H	4.605802	1.447348	0.715798
H	3.669791	2.145615	2.093078
H	3.947786	0.386949	2.005218
H	-1.616276	3.960798	-2.823750
H	-0.613668	3.378096	-4.191197
H	-2.151022	2.550710	-3.789329
O	-5.292194	-0.755285	-2.667616
H	-2.099331	-4.106395	-1.951484
H	-1.575005	-3.494078	-3.556089
H	-0.385484	-4.253836	-2.456292
H	-2.494710	3.487237	0.865072
H	-3.396677	3.329710	-0.672830
H	-3.836163	2.315871	0.755448
O	4.178329	-3.637989	-2.760180
H	0.024338	0.302873	-4.759033
H	1.754765	-0.147552	-4.858369
H	0.497288	-1.425444	-4.757488
H	3.933237	-2.637830	2.045699
H	2.470990	-2.480652	3.071381
H	2.499703	-3.696926	1.772950
C	3.799372	5.767759	-0.748003
C	-6.312397	0.215812	-2.415487
C	5.029688	-4.480647	-1.978045
H	4.648595	5.106731	-0.965456
H	4.022783	6.777947	-1.094018
H	3.608653	5.779575	0.332940
H	-6.058537	1.184700	-2.865862
H	-7.213885	-0.180704	-2.884688

H	-6.477995	0.341642	-1.337810
H	5.610539	-5.057179	-2.699366
H	5.706273	-3.886584	-1.349859
H	4.442353	-5.160203	-1.346983
H	-5.912921	0.504563	2.922716
H	-2.744349	-1.579618	0.920851
H	-0.375485	-2.659060	0.327559
H	-0.476089	-1.446129	4.448052
H	-0.370232	2.507189	1.760341
H	1.560670	-0.624237	3.959339
H	2.521227	3.476606	4.786761
H	-0.493153	-5.616348	3.427104
H	-1.837920	1.696106	3.544603

73. FLP$_{11}$: Acid R = H. Base R = NH$_2$

E$_{elec}$ = -2255.494527 a.u.; **H =** -2254.758917 a.u

C	1.355980	-2.000857	2.132168
C	2.284381	-1.255344	1.338593
C	3.654064	-1.262530	1.739329
C	4.049288	-1.929364	2.907766
C	3.113654	-2.616496	3.688011
C	1.773376	-2.666942	3.290610
P	1.546966	-0.129068	0.115845
C	2.364927	1.487291	0.368731
C	3.369628	2.079650	-0.438814
C	3.762058	3.408577	-0.247444
C	3.188295	4.180072	0.769235
C	2.228291	3.607922	1.609336
C	1.823133	2.280021	1.422593
N	4.059370	1.303482	-1.380040
N	3.531323	5.531125	0.907705
N	0.922525	1.726285	2.327870
N	4.633773	-0.701520	0.943726
N	3.509553	-3.214379	4.888436
N	0.040331	-2.115907	1.733393
C	1.806401	-0.653735	-1.622242
C	2.347519	-1.902461	-2.049664
C	2.329944	-2.258310	-3.409075
C	1.757359	-1.421371	-4.369309
C	1.183992	-0.209893	-3.969705
C	1.195744	0.165972	-2.620864
N	2.836536	-2.831551	-1.152355
N	1.683422	-1.828046	-5.706822
N	0.665451	1.404749	-2.281063
C	-1.247833	-2.191028	-1.787463
C	-1.458123	-3.409680	-1.141203
C	-2.424422	-3.509337	-0.136758
C	-3.186785	-2.396098	0.207986
C	-2.988066	-1.141497	-0.414218
C	-1.989037	-1.071743	-1.410788
B	-3.863186	0.100925	-0.047495
C	-5.339692	-0.099323	0.440296
C	-5.924796	0.770334	1.386156
C	-7.231766	0.589600	1.835037
C	-8.006326	-0.453722	1.322824

C	-7.461656	-1.320301	0.372505
C	-6.143761	-1.151855	-0.048515
C	-3.287838	1.553358	-0.171312
C	-1.929077	1.833598	0.087553
C	-1.418817	3.129517	0.003712
C	-2.254346	4.181345	-0.379454
C	-3.601177	3.933844	-0.656247
C	-4.110267	2.641722	-0.536322
H	-5.333713	1.592381	1.783261
H	-7.650937	1.263876	2.577827
H	-9.030334	-0.589958	1.661719
H	-8.063991	-2.128896	-0.034205
H	-5.727298	-1.836064	-0.783908
H	-3.943264	-2.486340	0.984375
H	-2.578433	-4.455412	0.376367
H	-0.856998	-4.274055	-1.409525
H	-0.496255	-2.109061	-2.566733
H	-1.803052	-0.124422	-1.909444
H	-1.261246	1.019373	0.363222
H	-0.370914	3.321178	0.223848
H	-1.855722	5.189613	-0.460438
H	-4.253220	4.749834	-0.958269
H	-5.162841	2.462326	-0.743142
H	4.520947	3.841926	-0.896791
H	1.803180	4.186653	2.427254
H	2.735300	-3.224419	-3.704155
H	0.724930	0.449069	-4.703948
H	5.097908	-1.920286	3.199790
H	1.049919	-3.227774	3.878931
H	-0.032537	1.771935	-2.916079
H	0.414110	1.539456	-1.306872
H	1.595352	-1.072685	-6.376589
H	2.392117	-2.495790	-5.987600
H	3.514985	-3.489212	-1.510636
H	2.995692	-2.524874	-0.198310
H	0.276549	2.382517	2.751206
H	0.464542	0.873489	2.009140
H	4.583787	1.848294	-2.055534
H	3.489714	0.586469	-1.832772
H	3.383932	5.910481	1.836123
H	4.449007	5.772200	0.550898
H	-0.648520	-2.376405	2.424366
H	-0.287715	-1.493910	0.999343
H	2.915579	-3.972158	5.204229
H	4.491528	-3.459054	4.940228
H	5.505591	-0.475765	1.403746
H	4.382283	-0.038423	0.214652

74. FLP_{12}: Acid R = CN. Base R = CN

E_{elec} = -3418.189091 a.u.; **H =** -3417.611595 a.u

C	4.562162	2.667571	0.293618
C	3.652310	1.645458	-0.073754
C	2.343550	2.053319	-0.431503
C	1.958792	3.396887	-0.428217
C	2.879282	4.376251	-0.033163

C	4.181740	4.012264	0.332403
B	4.064820	0.132389	-0.065617
C	5.423107	-0.321298	-0.705708
C	5.855567	0.179316	-1.959346
C	7.054952	-0.234775	-2.545982
C	7.881320	-1.141705	-1.870407
C	7.497081	-1.643700	-0.620485
C	6.277986	-1.250309	-0.061106
C	5.042546	1.108477	-2.685726
N	4.395556	1.868606	-3.280831
C	9.117801	-1.555067	-2.456747
N	10.122539	-1.891221	-2.933348
C	5.925629	-1.788121	1.218933
N	5.645008	-2.235939	2.253979
C	1.383775	1.077624	-0.851813
N	0.598877	0.289480	-1.187828
C	2.488130	5.751071	-0.003271
N	2.171701	6.868597	0.021168
C	5.900675	2.332779	0.677941
N	6.990010	2.064229	0.981228
C	3.115390	-0.926522	0.599769
C	2.816718	-2.160484	-0.030993
C	1.942123	-3.090067	0.537637
C	1.390136	-2.835956	1.798637
C	1.706619	-1.654660	2.478160
C	2.519673	-0.697418	1.864130
C	0.450695	-3.742823	2.379026
N	-0.354301	-4.432660	2.854266
C	3.368240	-2.458288	-1.318676
N	3.822988	-2.708185	-2.358496
C	2.726511	0.531145	2.570058
N	2.831141	1.530515	3.154507
C	-1.975519	-2.643603	-1.143080
C	-3.352619	-2.529400	-1.515127
C	-4.099504	-1.383512	-1.139567
C	-5.473907	-1.375167	-1.468831
C	-6.058652	-2.449228	-2.155477
C	-5.295597	-3.563945	-2.515336
C	-3.937403	-3.609324	-2.179526
P	-3.093416	-0.000230	-0.472447
C	-2.685748	-0.380261	1.274418
C	-3.043949	-1.523544	2.024284
C	-2.469216	-1.775362	3.278000
C	-1.554656	-0.873630	3.830640
C	-1.214565	0.291273	3.130587
C	-1.758337	0.518014	1.865276
C	-6.363741	-0.320978	-1.091524
C	-5.901245	-4.654729	-3.212234
C	-4.043123	-2.449124	1.589599
C	-0.940064	-1.146835	5.090986
C	-1.395641	1.723280	1.184539
C	-4.180314	1.478064	-0.434667
C	-4.790856	2.087105	0.684494
C	-5.451547	3.316579	0.563011
C	-5.538471	3.955386	-0.678691

C	-4.965323	3.359916	-1.808950
C	-4.285822	2.146469	-1.679839
C	-4.830015	1.467091	1.972183
C	-6.220799	5.205879	-0.794663
C	-3.729175	1.540652	-2.851911
H	7.345571	0.148580	-3.517678
H	8.139693	-2.337785	-0.090481
H	1.680716	-3.992099	-0.001149
H	1.312608	-1.474322	3.471474
H	0.949004	3.672806	-0.708126
H	4.892883	4.769445	0.643296
H	-5.916376	3.760594	1.436240
H	-5.041272	3.837021	-2.779558
H	-2.742534	-2.674041	3.818972
H	-0.520060	1.007533	3.556958
H	-7.116681	-2.415876	-2.390825
H	-3.337707	-4.476376	-2.432260
N	-0.398848	-1.369944	6.094741
N	-4.863046	-3.217993	1.295140
N	-1.117529	2.725582	0.665979
N	-4.903502	1.002663	3.034959
N	-6.773585	6.223137	-0.890097
N	-3.321554	1.036251	-3.817060
N	-0.877056	-2.805786	-0.802049
N	-7.149398	0.488020	-0.810852
N	-6.391704	-5.540523	-3.781713

75. FLP_{13}: Acid R = CF_3. Base R = CF_3
E_{elec} = -7826.857711 a.u.; **H =** -7826.144584 a.u

B	-4.122413	0.261184	-0.351725
C	-4.526345	-0.992023	-1.268293
C	-5.307030	-0.846401	-2.452872
C	-4.138592	-2.324932	-0.966656
C	-5.669169	-1.935143	-3.242708
C	-4.508292	-3.411943	-1.761068
C	-5.274268	-3.220596	-2.898209
H	-6.263633	-1.779165	-4.134520
H	-4.180946	-4.409425	-1.493061
C	-3.498496	1.567757	-1.037320
C	-3.948701	2.881233	-0.731076
C	-2.434933	1.498685	-1.984453
C	-3.364678	4.018674	-1.288106
C	-1.855375	2.641062	-2.534608
C	-2.310880	3.902126	-2.180644
H	-3.744107	4.999770	-1.026305
H	-1.037164	2.546340	-3.236205
C	-4.330686	0.203989	1.241656
C	-3.350725	0.675496	2.165245
C	-5.507398	-0.333387	1.838947
C	-3.536300	0.610126	3.545253
C	-5.672244	-0.416054	3.222002
C	-4.689156	0.054225	4.077415
H	-2.760932	0.967539	4.210539
H	-6.578988	-0.843777	3.631378
C	-5.713069	-4.395374	-3.739801

C	-3.286537	-2.748079	0.224712
C	-5.819897	0.487110	-2.993749
C	-6.719145	-0.848905	1.063487
C	-4.884424	0.001691	5.574062
C	-1.992099	1.250287	1.767213
C	-5.093893	3.228075	0.217237
C	-1.816476	0.196408	-2.492040
C	-1.649413	5.145306	-2.726112
F	-5.444276	1.152714	6.037028
F	-5.701648	-1.014956	5.941701
F	-3.708225	-0.158751	6.229308
F	-2.045301	2.576020	1.478448
F	-1.087821	1.128993	2.778698
F	-1.441299	0.629715	0.700851
F	-6.948167	-0.185201	-0.090933
F	-7.857203	-0.728576	1.796491
F	-6.607314	-2.168525	0.751890
F	-5.977194	2.230546	0.431545
F	-4.629919	3.613257	1.437865
F	-5.814577	4.275116	-0.266544
F	-0.782390	5.676750	-1.819884
F	-2.558815	6.111076	-3.012541
F	-0.944770	4.897305	-3.855306
F	-2.551484	-0.363780	-3.491376
F	-0.579152	0.398293	-3.002657
F	-1.680754	-0.747187	-1.529157
F	-5.759598	-4.078480	-5.057927
F	-6.959351	-4.812569	-3.387100
F	-4.882670	-5.457259	-3.602072
F	-6.144930	1.380303	-2.032824
F	-4.908291	1.090546	-3.803690
F	-6.940483	0.317079	-3.742626
F	-2.436260	-3.754632	-0.124983
F	-2.509955	-1.770077	0.740050
F	-4.049113	-3.225897	1.242226
P	3.682130	-0.224189	-0.270380
C	5.162720	-1.340934	-0.405735
C	2.458220	-0.881342	0.965410
C	4.177196	1.533029	0.084517
C	5.816172	-1.262281	-1.672517
C	5.611229	-2.366907	0.467007
C	2.113933	-0.394959	2.256159
C	1.667361	-1.956627	0.466588
C	5.409091	2.052033	0.562343
C	3.228494	2.493425	-0.375899
C	6.885629	-2.091064	-1.996903
C	6.663902	-3.213067	0.105709
C	1.132334	-1.023461	3.020127
C	0.701827	-2.582300	1.254968
C	5.634554	3.429738	0.627445
C	3.458398	3.862005	-0.271957
C	7.314507	-3.066607	-1.107288
H	7.367625	-1.994150	-2.961021
H	6.979647	-3.991324	0.788271
C	0.451736	-2.132986	2.540859

H	0.900493	-0.642144	4.006185
H	0.134494	-3.410915	0.854250
C	4.659972	4.333598	0.238037
H	6.584849	3.796882	0.992790
H	2.707689	4.561692	-0.616213
C	-0.564371	-2.804890	3.431090
C	2.699665	0.852601	2.905227
C	1.767492	-2.486274	-0.965903
C	4.993937	-2.723549	1.814491
C	8.493817	-3.940480	-1.459304
C	5.367973	-0.330682	-2.800612
C	6.618382	1.219940	0.971450
C	4.891129	5.818334	0.382510
C	1.931294	2.116702	-1.088904
F	9.669707	-3.321828	-1.163943
F	8.523928	-4.233472	-2.784002
F	8.479429	-5.113665	-0.780665
F	5.284285	0.972759	-2.419123
F	6.240493	-0.352192	-3.840718
F	4.164889	-0.691037	-3.303120
F	5.854540	-3.441128	2.582584
F	4.626481	-1.665347	2.561441
F	3.890091	-3.510282	1.650712
F	3.014936	-2.934154	-1.282363
F	0.936284	-3.540560	-1.165723
F	1.424217	-1.551680	-1.879353
F	-1.550152	-1.939450	3.805235
F	-1.160800	-3.860925	2.834105
F	0.009011	-3.254289	4.578816
F	2.370068	0.941005	4.218901
F	4.046030	0.922824	2.853919
F	2.210504	1.982462	2.318350
F	4.417023	6.275727	1.573590
F	6.208725	6.134325	0.331488
F	4.262375	6.523115	-0.590876
F	2.168170	1.604920	-2.319100
F	1.129855	3.201010	-1.274405
F	1.177057	1.218894	-0.403713
F	7.304978	0.787176	-0.126133
F	7.501939	1.946296	1.703571
F	6.322546	0.136690	1.714896

76. FLP_{14}: Acid R = CN. Base R = H

$\mathbf{E_{elec}}$ **=** 2587.589330 a.u.; **H =** -2587.011871 a.u

C	3.291661	2.545941	0.304860
C	2.327386	1.680767	-0.259344
C	1.385787	2.251923	-1.144164
C	1.368413	3.621668	-1.419380
C	2.302203	4.460657	-0.796019
C	3.272669	3.922289	0.059729
B	2.289972	0.146624	0.089198
C	3.560720	-0.740387	-0.179055
C	4.336839	-0.599399	-1.357721
C	5.455682	-1.400120	-1.610313
C	5.861566	-2.346492	-0.661979

C	5.141817	-2.492804	0.530069
C	4.003489	-1.712538	0.755749
C	3.984907	0.358720	-2.362774
N	3.738589	1.133776	-3.192755
C	7.012773	-3.157760	-0.907372
N	7.948138	-3.817254	-1.106977
C	3.322806	-1.910052	2.001263
N	2.799031	-2.103038	3.020467
C	0.521071	1.368088	-1.860280
N	-0.114091	0.607375	-2.466675
C	2.275900	5.867918	-1.048231
N	2.251150	7.011676	-1.250588
C	4.293511	2.002367	1.171502
N	5.108923	1.529254	1.851073
C	1.037928	-0.484517	0.783953
C	0.513132	-1.733995	0.370777
C	-0.554964	-2.343068	1.033892
C	-1.096002	-1.733642	2.172089
C	-0.594101	-0.507891	2.621192
C	0.437442	0.118420	1.916531
C	-2.181545	-2.351277	2.867401
N	-3.069506	-2.842960	3.432235
C	1.078316	-2.396275	-0.766089
N	1.561172	-2.936067	-1.674985
C	0.924311	1.365502	2.423127
N	1.310434	2.376428	2.846372
P	-3.276289	0.296006	-1.065198
C	-3.079859	0.931482	0.662476
C	-3.796418	0.483835	1.784287
C	-3.640708	1.102879	3.023850
C	-2.780891	2.197046	3.161007
C	-2.071984	2.662702	2.053870
C	-2.217847	2.027588	0.818113
C	-3.067553	-1.541596	-0.985995
C	-2.139266	-2.099472	-1.879522
C	-1.962010	-3.484049	-1.954835
C	-2.703690	-4.328809	-1.128577
C	-3.632178	-3.785599	-0.234579
C	-3.822133	-2.405981	-0.175099
C	-5.111386	0.403954	-1.235639
C	-5.828000	1.472958	-0.672719
C	-7.195189	1.616801	-0.907300
C	-7.872321	0.701226	-1.713949
C	-7.168555	-0.358372	-2.288490
C	-5.801636	-0.504314	-2.055937
H	-5.318697	2.192900	-0.037655
H	-8.938202	0.812040	-1.892885
H	-5.270172	-1.337716	-2.507731
H	-4.578528	-2.008464	0.492824
H	-2.565927	-5.405461	-1.182686
H	-1.553726	-1.441215	-2.514542
H	-4.496890	-0.338635	1.692601
H	-2.668836	2.686331	4.124706
H	-1.671665	2.393801	-0.047820
H	6.008821	-1.282544	-2.535456

H	5.462384	-3.211455	1.275998
H	-0.965350	-3.276064	0.665014
H	-1.019037	-0.031047	3.495598
H	0.646658	4.029232	-2.118497
H	4.004257	4.569119	0.530670
H	-7.732338	2.445970	-0.453945
H	-7.684755	-1.078066	-2.918639
H	-4.198533	0.735326	3.881040
H	-1.402737	3.511966	2.153002
H	-4.214066	-4.436002	0.412621
H	-1.237612	-3.895504	-2.651633

77. FLP_{15}: Acid R = CF_3. Base R = H

E_{elec} = -4791.929719 a.u.; **H =** -4791.284826 a.u

B	-1.601455	-0.095905	-0.330263
C	-2.589765	-1.258261	0.171114
C	-2.890273	-2.407708	-0.609279
C	-3.229764	-1.208814	1.443374
C	-3.740336	-3.416923	-0.152708
C	-4.075070	-2.220900	1.892528
C	-4.329582	-3.329322	1.097228
H	-3.938228	-4.276890	-0.781209
H	-4.528776	-2.149072	2.873301
C	-0.179819	-0.518164	-0.950839
C	0.338055	0.045047	-2.155077
C	0.616122	-1.557768	-0.390195
C	1.489989	-0.440731	-2.770321
C	1.776071	-2.031451	-1.007668
C	2.198627	-1.493831	-2.212002
H	1.842966	0.011177	-3.687847
H	2.355315	-2.813833	-0.535560
C	-2.112327	1.426647	-0.219296
C	-1.352586	2.465015	0.377214
C	-3.398796	1.831534	-0.691645
C	-1.814784	3.779811	0.478670
C	-3.853297	3.143771	-0.590031
C	-3.061997	4.125221	-0.007246
H	-1.190322	4.527588	0.954740
H	-4.835490	3.402839	-0.965546
C	-5.277578	-4.401495	1.578708
C	-3.079747	-0.059757	2.436050
C	-2.341480	-2.712626	-2.002361
C	-4.418301	0.892433	-1.328128
C	-3.555787	5.550067	0.057937
C	0.013118	2.327458	1.036018
C	-0.304444	1.216770	-2.882078
C	0.330204	-2.239588	0.943864
C	3.405741	-2.049792	-2.927822
F	-3.356340	6.194353	-1.124269
F	-4.886409	5.607842	0.319127
F	-2.919088	6.266926	1.015569
F	0.930685	3.108721	0.399248
F	-0.038538	2.770634	2.320877
F	0.516054	1.078836	1.074091
F	-3.865743	-0.069076	-2.100094

F	-5.286069	1.563481	-2.129089
F	-5.178601	0.263578	-0.388478
F	-0.047612	2.398072	-2.247865
F	0.170689	1.361180	-4.144682
F	-1.649339	1.110714	-2.998255
F	4.080968	-1.091810	-3.604403
F	3.035112	-2.993885	-3.839929
F	4.277712	-2.646821	-2.077799
F	-0.464801	-3.338714	0.810625
F	1.473004	-2.677957	1.531255
F	-0.266269	-1.424834	1.843760
F	-5.043491	-5.593965	0.978900
F	-6.570893	-4.068378	1.316278
F	-5.182248	-4.590713	2.918899
F	-1.925571	-1.639801	-2.706378
F	-1.290467	-3.575961	-1.935716
F	-3.288106	-3.320487	-2.767941
F	-3.314550	-0.468377	3.709713
F	-1.849577	0.502910	2.446813
F	-3.971809	0.938512	2.185644
P	4.406414	-0.448937	0.799247
C	6.122498	-0.858116	1.319316
C	3.806226	0.646366	2.148218
C	4.682600	0.740292	-0.576967
C	6.742593	-1.944739	0.680639
C	6.828588	-0.162992	2.311213
C	4.027341	2.031195	2.199091
C	3.066130	0.041015	3.175997
C	5.902404	0.877107	-1.253897
C	3.572623	1.468264	-1.039626
C	8.048100	-2.310605	1.007298
H	6.197900	-2.501544	-0.078423
C	8.128327	-0.541177	2.648929
H	6.359255	0.672292	2.823148
C	3.520985	2.789238	3.254383
H	4.588281	2.516735	1.405596
C	2.568971	0.798347	4.235711
H	2.870440	-1.027462	3.135380
C	6.006402	1.719281	-2.362736
H	6.775569	0.328402	-0.912908
C	3.681177	2.319656	-2.136008
H	2.620139	1.376220	-0.526861
C	8.743047	-1.610585	1.995223
H	8.517550	-3.148748	0.499018
H	8.663116	0.003522	3.422827
C	2.793129	2.175152	4.275228
H	3.693393	3.862172	3.277938
H	1.993346	0.316054	5.021183
C	4.900250	2.443421	-2.807223
H	6.959817	1.812351	-2.876355
H	2.811767	2.881879	-2.466847
H	9.756211	-1.901566	2.259279
H	2.392721	2.769431	5.092051
H	4.986278	3.100646	-3.668175

78. FLP$_{17}$: Acid R = CF$_3$. Base R = OCH$_3$

E$_{elec}$ = -5821.167671 a.u.; **H =** -5820.210638 a.u

C	-1.389027	2.317139	0.491579
C	-1.296505	0.944383	0.901981
C	-1.331119	0.859134	2.338939
C	-1.640655	1.926303	3.188026
C	-1.871856	3.195828	2.696251
C	-1.688859	3.387961	1.344438
B	-1.488641	-0.338008	-0.342662
C	-2.239272	-1.860400	0.163114
C	-3.594725	-2.198065	-0.224244
C	-4.475526	-2.890324	0.612434
C	-4.041977	-3.461935	1.795943
C	-2.672540	-3.530755	1.973898
C	-1.801071	-2.798074	1.160515
C	-2.824420	0.453156	-1.219979
C	-3.927017	0.973897	-0.447523
C	-4.899541	1.844112	-0.957407
C	-4.902359	2.224140	-2.282774
C	-3.963592	1.642286	-3.107469
C	-2.982563	0.775694	-2.607286
H	-1.711010	1.748684	4.253489
H	-1.789964	4.382223	0.925247
H	-5.676334	2.212732	-0.298110
H	-3.985303	1.854336	-4.169868
H	-5.507226	-3.012527	0.303565
H	-2.259242	-4.156250	2.756214
C	-2.191363	4.339053	3.614609
C	-1.024738	-0.371872	3.171704
C	-1.162913	2.910661	-0.889440
C	-2.228997	0.251197	-3.822570
C	-5.885780	3.232552	-2.805792
C	-4.372393	0.672044	0.990430
C	-4.219751	-2.105606	-1.614622
C	-4.995382	-4.073602	2.778610
C	-0.401152	-3.356252	1.301981
F	-4.457820	-5.152217	3.413577
F	-5.350840	-3.189534	3.754658
F	-6.149555	-4.487922	2.191526
F	-0.036490	-3.658796	2.587270
F	0.593711	-2.652947	0.762882
F	-0.392292	-4.591096	0.668294
F	-4.834042	-3.308978	-1.894176
F	-3.328571	-1.948112	-2.609531
F	-5.205912	-1.182868	-1.772961
F	-3.685595	-0.211429	1.725619
F	-4.429533	1.823998	1.731303
F	-5.656492	0.190798	0.963486
F	-7.055139	3.217902	-2.114496
F	-6.186717	3.025733	-4.115048
F	-5.396320	4.504132	-2.715839
F	-3.119473	-0.271199	-4.730154
F	-1.620631	1.279318	-4.498935
F	-1.315675	-0.703777	-3.633628
F	-2.096590	-1.145938	3.475191

F	-0.500878	-0.035665	4.396041
F	-0.092758	-1.135282	2.582009
F	-1.047700	4.916263	4.107367
F	-2.917012	3.948680	4.691708
F	-2.880674	5.326030	2.990254
F	-0.277792	3.964926	-0.785276
F	-0.656886	2.102059	-1.821275
F	-2.295229	3.476940	-1.400698
N	0.005893	-0.661263	-1.082704
N	1.200805	0.157146	-1.184375
C	0.185924	-1.763292	-1.934263
C	1.671730	0.495909	-2.468556
O	1.105811	-1.879842	-2.731797
O	-0.772797	-2.702730	-1.794692
O	2.894937	0.617701	-2.583226
O	0.806854	0.782247	-3.412594
C	-0.842678	-3.690600	-2.873383
C	1.389933	1.023781	-4.739114
C	-1.655244	-4.871000	-2.380363
H	0.175168	-3.972043	-3.149732
H	-1.314353	-3.199020	-3.728554
C	1.689382	-0.288193	-5.441994
H	2.286983	1.633949	-4.608455
H	0.615688	1.596880	-5.247795
H	-1.754130	-5.594342	-3.197436
H	-1.167060	-5.363413	-1.536480
H	-2.657047	-4.565848	-2.072781
H	2.067885	-0.074473	-6.448483
H	2.440203	-0.858431	-4.890951
H	0.783690	-0.893522	-5.523958
P	2.835422	-0.015792	-0.341443
C	3.705364	1.598099	-0.109993
C	2.501109	-0.317449	1.425090
C	4.049447	-1.200689	-1.062468
C	3.385876	2.843839	-0.695780
C	4.827195	1.576465	0.747665
C	2.055475	0.824410	2.150753
C	3.001167	-1.384295	2.208099
C	5.279746	-0.752792	-1.626885
C	3.809517	-2.596570	-1.174647
C	4.155713	3.983658	-0.452014
O	2.264597	2.916029	-1.454361
C	5.581732	2.718863	1.038356
O	5.198741	0.351027	1.219285
C	2.140532	0.917407	3.532160
O	1.533676	1.841266	1.412788
C	3.006759	-1.341242	3.607947
O	3.552168	-2.430723	1.553487
C	6.259001	-1.641113	-2.090723
O	5.491449	0.580514	-1.724975
C	4.774720	-3.487350	-1.646113
O	2.589517	-3.037194	-0.815889
C	5.240370	3.922193	0.421253
H	3.892992	4.940996	-0.883426
C	2.132437	4.011891	-2.370054

H	6.437478	2.646812	1.694845
C	6.152708	0.265580	2.278719
C	2.588642	-0.183976	4.261315
H	1.771129	1.783141	4.065348
C	1.623503	3.170550	1.960053
H	3.366511	-2.192367	4.167712
C	3.934222	-3.599499	2.278684
C	6.006882	-3.009723	-2.079727
H	7.187352	-1.251295	-2.481674
C	6.721524	1.079280	-2.245631
H	4.583965	-4.550375	-1.700103
C	2.240545	-4.386597	-1.139692
O	5.909853	5.098840	0.616820
H	1.933106	4.951444	-1.842988
H	3.035533	4.105064	-2.984419
H	1.272938	3.760528	-2.989905
H	5.834839	0.866540	3.139049
H	6.174125	-0.789489	2.553365
H	7.149969	0.578934	1.943480
O	2.569811	-0.038518	5.615483
H	2.653228	3.375712	2.272423
H	1.333440	3.831666	1.145939
H	0.935012	3.297381	2.799976
H	4.741025	-3.384053	2.991836
H	3.073664	-4.033049	2.801872
H	4.299407	-4.292612	1.519794
O	6.896115	-3.955804	-2.503961
H	7.575048	0.750599	-1.638187
H	6.864522	0.772746	-3.289565
H	6.625976	2.164171	-2.187533
H	2.412776	-4.579865	-2.203919
H	2.810233	-5.098822	-0.529001
H	1.184905	-4.473174	-0.906354
C	7.000438	5.097076	1.544170
C	2.883585	-1.185281	6.416056
C	8.162822	-3.506367	-2.997845
H	7.362870	6.125325	1.568245
H	6.666194	4.798381	2.545989
H	7.805185	4.430137	1.207784
H	2.742077	-0.864372	7.448460
H	2.205853	-2.016693	6.187912
H	3.924958	-1.499261	6.266898
H	8.705789	-4.411991	-3.270588
H	8.037566	-2.869832	-3.883022
H	8.717752	-2.960977	-2.223618

79. FLP: Reference

E_{elec} = -2071.690882 a.u.; **H =** -2070.883312 a.u

C	-1.475640	3.719389	-3.510221
C	-2.430443	3.573027	-2.503308
C	-2.234400	2.730505	-1.402437
C	-1.033738	1.987419	-1.307330
C	-0.039377	2.152981	-2.304824
C	-0.278837	3.011056	-3.380989
P	-0.653007	0.742385	0.001549

C	-2.252558	-0.086952	0.397400
C	-2.708800	-0.940765	-0.647064
C	-3.812421	-1.769273	-0.439975
C	-4.498609	-1.799280	0.775329
C	-4.056839	-0.941616	1.781065
C	-2.958997	-0.084501	1.625933
B	1.727103	-2.232187	-0.087316
C	1.043816	-3.028281	-1.253729
C	1.128269	-2.592668	-2.594487
C	0.495550	-3.275352	-3.630697
C	-0.231098	-4.436288	-3.358218
C	-0.319010	-4.905310	-2.045721
C	0.304351	-4.206877	-1.013423
C	1.175133	-2.343185	1.377789
C	2.039043	-2.255480	2.491306
C	1.557334	-2.318439	3.798249
C	0.185812	-2.449279	4.030303
C	-0.694299	-2.538459	2.949125
C	-0.201922	-2.501018	1.645847
C	2.989969	-1.352443	-0.368558
C	3.939076	-1.685874	-1.357584
C	5.052057	-0.885543	-1.605535
C	5.257386	0.304065	-0.894482
C	4.324123	0.649148	0.093365
C	3.228938	-0.166040	0.357926
C	-0.057426	1.765622	1.396813
C	0.003463	3.165015	1.350995
C	0.613271	3.885867	2.379664
C	1.180749	3.241831	3.483870
C	1.128370	1.839661	3.521261
C	0.535008	1.114959	2.495402
H	2.247168	-2.253938	4.636226
H	-1.764379	-2.623131	3.117342
H	5.772784	-1.183871	-2.364818
H	4.459219	1.568189	0.661144
H	0.564084	-2.902455	-4.649613
H	-0.875432	-5.813939	-1.829293
H	0.643658	4.972323	2.321552
H	1.570007	1.304915	4.360192
H	-4.136892	-2.413917	-1.255663
H	-4.589570	-0.924669	2.731217
H	-3.357302	4.141994	-2.564269
H	0.495170	3.129184	-4.137964
H	-0.195010	-2.479015	5.048237
H	-0.902805	-2.550968	0.818483
H	1.683529	-1.686641	-2.821506
H	0.229946	-4.585661	0.002703
H	2.521255	0.137620	1.123395
H	3.810302	-2.601930	-1.929559
C	6.431864	1.201706	-1.198020
H	-0.724487	-4.973707	-4.164359
H	3.106324	-2.134985	2.322669
C	1.844905	4.020309	4.593785
H	0.525149	0.029919	2.546544
H	-0.426192	3.696127	0.506074

C	-2.622898	0.791891	2.809062
C	-5.662346	-2.735464	0.993600
C	1.295253	1.451157	-2.222839
C	-2.041302	-0.975388	-2.000051
C	-3.315623	2.684564	-0.349995
C	-1.730922	4.605258	-4.705588
H	-6.274492	-2.417644	1.843469
H	-6.303827	-2.786846	0.107065
H	-5.310170	-3.754659	1.200111
H	-2.319215	-1.881766	-2.545110
H	-2.321214	-0.104628	-2.605085
H	-0.949922	-0.959008	-1.903149
H	-3.485601	0.850456	3.479907
H	-1.780703	0.386476	3.381311
H	-2.343496	1.806082	2.516565
H	-3.973346	3.553806	-0.447956
H	-3.928600	1.780880	-0.436296
H	-2.889951	2.687381	0.657281
H	-0.806944	5.076271	-5.057005
H	-2.139331	4.021283	-5.540797
H	-2.453411	5.393289	-4.470491
H	1.879920	1.624672	-3.131456
H	1.883548	1.807711	-1.369385
H	1.176679	0.373331	-2.077360
H	1.416361	3.761955	5.569411
H	2.918260	3.798263	4.643225
H	1.730551	5.098412	4.446540
H	6.819412	1.671593	-0.287865
H	7.245789	0.647441	-1.675675
H	6.135361	2.009052	-1.880731

4. FLP-DEAD cartesian coordinates

80. Linker = DEAD

E_{elec} = -645.3876395 a.u.; **H =** -645.205943 a.u

N	-0.298324	0.697857	-0.862889
N	0.851645	0.252894	-0.688186
C	1.879083	1.275120	-0.719755
O	2.510018	1.511319	-1.720798
O	2.067243	1.766060	0.507748
C	-1.309099	-0.335791	-0.970056
O	-1.691365	-0.742374	-2.039877
O	-1.790128	-0.626058	0.241367
C	3.195476	2.701641	0.634218
C	3.281221	3.093171	2.094424
H	2.996863	3.555162	-0.020430
H	4.095306	2.191255	0.278211
H	2.357048	3.577457	2.422945
H	3.461591	2.216660	2.723522
C	-2.934343	-1.550689	0.248015
C	-3.368651	-1.703387	1.690551
H	-2.603894	-2.494542	-0.195217
H	-3.715162	-1.121852	-0.387082
H	-2.557208	-2.110429	2.300824
H	-3.674917	-0.740612	2.109881

H	4.109582	3.796176	2.232177
H	-4.219617	-2.390903	1.741059

81. FLP_3 - Linker. Acid R = NH_2. Base R = H

E_{elec} = -2900.952356 a.u.; **H =** -2900.030996 a.u

C	-4.279298	-1.550593	-1.823851
C	-4.358318	-0.441744	-0.966597
C	-5.603600	0.057990	-0.560457
C	-6.771295	-0.559075	-1.005840
C	-6.697910	-1.667856	-1.850982
C	-5.455265	-2.157811	-2.259130
P	-2.821297	0.344270	-0.452837
C	-2.213242	1.383474	-1.786431
C	-0.967632	2.022404	-1.661770
C	-0.534123	2.876108	-2.673032
C	-1.320511	3.080964	-3.808750
C	-2.550666	2.434714	-3.937054
C	-3.002870	1.589253	-2.925371
N	-1.588026	-0.802481	-0.202363
C	-1.909705	-1.803674	0.697957
O	-0.961171	-2.731801	0.791007
C	-1.192253	-3.788663	1.778275
C	-2.045739	-4.895046	1.182127
N	-0.307485	-0.696811	-0.797550
C	-0.356588	-1.154423	-2.072392
O	0.906468	-1.026785	-2.617646
C	1.078063	-1.599373	-3.932289
C	2.524694	-1.358697	-4.328794
B	2.707086	0.162407	0.944044
C	3.597421	-0.819725	0.099074
C	4.921160	-0.458621	-0.330701
C	5.703291	-1.304689	-1.127261
C	5.213305	-2.547418	-1.536059
C	3.936476	-2.947884	-1.135561
C	3.142865	-2.112443	-0.335846
C	1.771746	-0.344098	2.103604
C	2.119296	-1.470398	2.922280
C	1.235315	-2.002620	3.871435
C	-0.023628	-1.431011	4.061695
C	-0.386651	-0.298904	3.327058
C	0.495769	0.245925	2.384176
C	2.783775	1.716389	0.631861
C	2.949626	2.685664	1.670234
C	2.997995	4.061898	1.403868
C	2.891959	4.525381	0.087870
C	2.738360	3.611572	-0.959398
C	2.695705	2.230061	-0.701535
C	-3.185815	1.430298	0.959240
C	-3.788603	0.919556	2.122991
C	-4.031272	1.760438	3.207666
C	-3.683548	3.110776	3.150472
C	-3.103994	3.626671	1.990174
C	-2.861805	2.795924	0.897702
O	-1.301352	-1.608519	-2.734466
O	-2.982288	-1.792007	1.302840
H	-4.039258	-0.132399	2.174035

H	-3.868931	3.759481	4.002201
H	-2.411673	3.210440	0.002701
H	-0.348399	1.831873	-0.792213
H	-0.972964	3.743997	-4.596904
H	-3.957570	1.083218	-3.025749
H	-5.661647	0.918771	0.098365
H	-7.610648	-2.148973	-2.192443
H	-3.300357	-1.903006	-2.145824
H	-7.737179	-0.174819	-0.690055
H	-5.398678	-3.017838	-2.920543
H	-4.491395	1.353317	4.103893
H	-2.839695	4.678670	1.930351
H	0.417076	3.387017	-2.556742
H	-3.159212	2.584933	-4.824054
N	0.039964	1.356671	1.675647
N	3.397342	-2.003410	2.867813
H	-1.366294	0.152794	3.467987
H	1.542125	-2.856965	4.473061
N	-0.942832	-2.024505	4.938736
N	5.522159	0.707403	0.125102
N	1.900359	-2.584160	0.044247
H	6.713601	-1.003212	-1.399154
H	3.540161	-3.908557	-1.461888
N	6.016313	-3.406287	-2.294183
N	3.144912	2.260805	2.980001
N	2.583162	1.371801	-1.775932
H	3.159249	4.766146	2.218325
H	2.695537	3.969296	-1.987469
N	3.013916	5.893175	-0.186086
H	0.370796	-1.130749	-4.625994
H	0.842406	-2.669689	-3.888381
H	2.723524	-1.818646	-5.303658
H	2.738079	-0.287485	-4.403529
H	3.202303	-1.788492	-3.584472
H	-1.647682	-3.342314	2.664666
H	-0.180701	-4.120412	2.018330
H	-2.145278	-5.710301	1.908095
H	-3.044480	-4.520944	0.940721
H	-1.585045	-5.294998	0.273209
H	1.551439	-3.340163	-0.533303
H	1.174762	-1.878444	0.180126
H	5.508408	-4.095796	-2.836061
H	6.730161	-2.944426	-2.845791
H	6.273163	1.058180	-0.456039
H	4.884642	1.434035	0.436596
H	-0.627715	1.929458	2.177004
H	0.774176	1.895962	1.223751
H	-0.523673	-2.555751	5.693788
H	-1.663679	-1.398066	5.278825
H	3.470540	-2.975610	3.140673
H	3.910724	-1.791364	2.015074
H	2.208944	1.763397	-2.629376
H	2.263475	0.423681	-1.606262
H	2.911912	2.934046	3.698891
H	2.814817	1.319127	3.180493

H	2.585515	6.194125	-1.053704
H	2.771971	6.504706	0.584938

82. FLP_4 - Linker. Acid R = OCH3. Base R = H

E_{elec} = -3433.717301 a.u.; **H =** -3432.642363 a.u

C	-3.491482	0.593993	-3.198928
C	-3.877894	0.490222	-1.855202
C	-5.238339	0.433601	-1.519258
C	-6.204690	0.473857	-2.523502
C	-5.820922	0.564617	-3.861551
C	-4.465984	0.625608	-4.193636
P	-2.646164	0.502879	-0.520609
C	-2.421612	2.227122	-0.048988
C	-1.725735	2.575962	1.116605
C	-1.796646	3.877968	1.606216
C	-2.533160	4.846261	0.925919
C	-3.179629	4.517829	-0.268241
C	-3.132061	3.213747	-0.754994
H	-1.133351	1.833306	1.631121
H	-2.588512	5.860676	1.312015
H	-3.650724	2.961973	-1.672903
H	-5.544676	0.358399	-0.481038
H	-6.575674	0.589682	-4.643041
H	-2.437620	0.673902	-3.442726
N	-1.161221	-0.201582	-1.029796
C	-1.355394	-1.376703	-1.757159
O	-0.284542	-1.759242	-2.433544
C	-0.417104	-3.002277	-3.188266
C	-0.909429	-2.705816	-4.594555
N	0.111057	0.411546	-0.970714
C	0.237726	1.272668	-2.044149
O	1.486255	1.735256	-2.227372
C	1.662869	2.618403	-3.359510
C	3.117010	3.054090	-3.330871
B	1.176815	-0.075006	0.261017
C	1.920167	-1.522113	-0.181765
C	2.197102	-2.580014	0.724875
C	2.754290	-3.810625	0.362423
C	3.138631	-4.020933	-0.959130
C	3.005900	-2.993862	-1.884330
C	2.430300	-1.774308	-1.465130
O	1.925044	-2.334548	2.049802
C	2.299977	-3.288536	3.030643
O	3.663589	-5.266628	-1.246262
C	4.102550	-5.489745	-2.582821
O	2.378331	-0.723817	-2.350723
C	2.829765	-0.915451	-3.677923
C	0.178877	-0.337667	1.588597
C	-0.589757	-1.517528	1.719723
C	-1.235906	-1.937185	2.891470
C	-1.144419	-1.140149	4.024706
C	-0.427096	0.051244	3.978885
C	0.213057	0.420233	2.788870
O	-0.664241	-2.328021	0.607880
C	-0.979825	-3.706940	0.779542

O	0.919090	1.596769	2.748708
C	1.224749	2.278068	3.958321
O	-1.750421	-1.436125	5.227387
C	-2.302031	-2.746118	5.366543
C	2.325735	1.114329	0.493420
C	3.662336	0.817150	0.868066
C	4.685393	1.768237	0.986573
C	4.394618	3.111078	0.791188
C	3.088916	3.493936	0.500811
C	2.104774	2.500780	0.365683
O	3.944952	-0.489780	1.169514
C	5.296675	-0.923562	1.224941
O	5.449395	3.991596	0.928206
C	5.171112	5.372891	0.718712
O	0.809153	2.905857	0.108594
C	0.608980	4.207006	-0.433564
C	-3.455886	-0.330296	0.886277
C	-3.993546	-1.619646	0.711380
C	-4.706382	-2.225267	1.741179
C	-4.898024	-1.564002	2.955894
C	-4.364221	-0.290924	3.137622
C	-3.654298	0.327415	2.109731
H	-3.848443	-2.131019	-0.230588
H	-5.460835	-2.039142	3.755034
H	-3.265426	1.323905	2.271561
O	-0.694206	1.607288	-2.785855
O	-2.449892	-1.933130	-1.768820
H	-7.256886	0.430231	-2.256534
H	-4.162229	0.700905	-5.234147
H	-5.118047	-3.219420	1.588693
H	-4.488964	0.227895	4.083063
H	-1.253641	4.130999	2.511725
H	-3.730452	5.274267	-0.819771
H	-0.372478	0.658818	4.873764
H	-1.819010	-2.847292	2.895361
H	2.918683	-4.604011	1.081037
H	3.364938	-3.110891	-2.897551
H	5.695992	1.488961	1.257777
H	2.826965	4.538158	0.399808
H	0.970898	3.462884	-3.276102
H	1.406748	2.075690	-4.277248
H	3.316503	3.742770	-4.159443
H	3.348729	3.552794	-2.384570
H	3.781620	2.189959	-3.421344
H	-1.100128	-3.665464	-2.652031
H	0.593810	-3.414984	-3.168441
H	-0.934333	-3.632130	-5.179786
H	-1.918597	-2.284843	-4.565161
H	-0.243067	-1.995779	-5.093973
H	1.902295	3.082394	3.664476
H	0.325133	2.705057	4.426839
H	1.725341	1.614289	4.675191
H	-0.727191	-4.182768	-0.169106
H	-0.376310	-4.148400	1.580945
H	-2.047821	-3.853999	0.989995

H	-2.659658	-2.807358	6.396193
H	-3.140878	-2.903913	4.676546
H	-1.537949	-3.515346	5.191079
H	2.023261	-2.837886	3.985751
H	1.761264	-4.240141	2.910158
H	3.381464	-3.483632	3.008880
H	2.657867	0.034759	-4.186207
H	3.902166	-1.157289	-3.716250
H	2.265023	-1.705293	-4.195739
H	4.472119	-6.516869	-2.606092
H	3.276330	-5.380610	-3.300271
H	4.913327	-4.801172	-2.859494
H	-0.390702	4.198027	-0.870759
H	0.657502	4.984020	0.342973
H	1.348435	4.429989	-1.210918
H	6.124696	5.889410	0.844988
H	4.785477	5.554586	-0.294551
H	4.448275	5.754137	1.454133
H	5.243412	-2.013737	1.275917
H	5.850615	-0.625262	0.324628
H	5.813954	-0.537782	2.115578

83. FLP_5 - Linker. Acid R = NH_2. Base R = NH_2

E_{elec} = -3399.468826 a.u.; **H =** -3398.389200 a.u

C	-2.304090	0.101888	2.716764
C	-3.346302	-0.544008	1.978781
C	-3.949437	-1.674608	2.622074
C	-3.552292	-2.098410	3.898984
C	-2.543186	-1.423312	4.588963
C	-1.921026	-0.316889	3.998114
B	-3.787097	-0.045160	0.557072
N	0.149052	-0.166127	-0.833533
N	1.165695	0.637318	-0.224911
P	2.790514	0.067556	-0.051485
C	3.102928	-0.895909	1.435144
C	2.103060	-1.309488	2.373529
C	2.389732	-2.334463	3.289256
C	3.632758	-2.966486	3.299282
C	4.633146	-2.557136	2.409277
C	4.389141	-1.535557	1.488775
N	0.873172	-0.723696	2.451195
N	3.910010	-3.951245	4.249146
N	5.440206	-1.123429	0.694121
N	-5.036637	-2.317009	2.045708
N	-2.102971	-1.888575	5.833057
N	-1.560275	1.106344	2.116890
C	-4.145547	-1.108420	-0.547262
C	-5.334688	-1.008444	-1.336880
C	-5.696705	-2.001792	-2.255945
C	-4.884277	-3.125262	-2.438343
C	-3.703396	-3.250239	-1.701776
C	-3.329264	-2.257063	-0.788287
N	-5.205980	-4.075459	-3.414867
N	-6.230784	0.037690	-1.127630
N	-2.123128	-2.428636	-0.109817

C	-3.926291	1.491787	0.238468
C	-4.472236	2.426514	1.185509
C	-4.699828	3.771165	0.856121
C	-4.363147	4.256056	-0.410941
C	-3.800118	3.392975	-1.355115
C	-3.587624	2.040168	-1.047261
N	-4.772907	2.031862	2.475291
N	-4.508446	5.615970	-0.704681
N	-3.058517	1.244181	-2.042560
C	0.074171	0.027966	-2.164863
O	0.686908	0.814459	-2.907092
O	-0.879761	-0.823924	-2.682865
C	-1.046698	-0.751841	-4.121247
C	-2.250548	-1.602179	-4.478839
C	0.824967	1.869321	0.352615
O	1.412692	2.348884	1.311079
O	-0.208787	2.440019	-0.293436
C	-0.555352	3.783964	0.164667
C	0.309565	4.810307	-0.549021
C	3.086751	-1.174797	-1.347432
C	2.421742	-2.443795	-1.164962
C	2.896379	-3.602074	-1.792721
C	4.019700	-3.568176	-2.617302
C	4.658410	-2.344855	-2.839231
C	4.217903	-1.168831	-2.223408
N	1.346218	-2.543178	-0.320076
N	4.520882	-4.738917	-3.186488
N	4.994192	-0.026904	-2.398564
C	3.766336	1.578968	-0.077059
C	4.432130	2.107325	1.088700
C	5.081596	3.350124	1.023550
C	5.031381	4.147276	-0.115414
C	4.306451	3.699205	-1.224654
C	3.673939	2.456518	-1.210114
N	4.500103	1.459490	2.293322
N	5.716635	5.358130	-0.167267
N	3.036498	2.056627	-2.376952
H	5.603856	3.704630	1.909979
H	4.226890	4.320447	-2.114509
H	1.616005	-2.636789	3.991872
H	5.622900	-3.006142	2.454512
H	2.357043	-4.534968	-1.640147
H	5.523866	-2.295369	-3.497612
H	0.665011	-1.755545	-0.371997
H	0.957441	-3.469642	-0.203990
H	3.851866	-5.494234	-3.277919
H	5.065426	-4.609599	-4.031066
H	5.719534	-0.151386	-3.096517
H	4.440870	0.821352	-2.574374
H	6.157352	-1.810760	0.508848
H	5.301634	-0.470587	-0.071810
H	4.644369	-4.600612	3.993493
H	3.097245	-4.423405	4.627253
H	0.524451	-0.108643	1.731426
H	0.146484	-1.195007	2.974532

H	2.155423	1.500395	-2.354046
H	2.947830	2.801870	-3.059124
H	5.924028	5.781448	0.729200
H	5.365290	6.029658	-0.839136
H	3.849865	0.702686	2.474385
H	4.739660	2.032345	3.089555
H	-4.050794	-2.948723	4.361706
H	-1.117985	0.202081	4.518250
H	-5.119704	4.443954	1.602033
H	-3.516937	3.769656	-2.336485
H	-6.622626	-1.902751	-2.820261
H	-3.042416	-4.097907	-1.874992
H	-0.127022	-1.110134	-4.598900
H	-1.185150	0.294369	-4.415463
H	-2.411190	-1.571203	-5.562622
H	-2.101220	-2.643280	-4.179362
H	-3.151954	-1.235433	-3.979708
H	-0.420417	3.833097	1.248436
H	-1.611749	3.883098	-0.089927
H	0.005721	5.819564	-0.248260
H	1.364386	4.668041	-0.294955
H	0.189319	4.719204	-1.633283
H	-1.666204	-1.185695	6.418537
H	-2.782288	-2.437664	6.347128
H	-5.191792	-3.266167	2.361375
H	-5.115292	-2.210136	1.037765
H	-1.076670	1.743690	2.736933
H	-1.994540	1.552709	1.314070
H	-5.451975	2.582739	2.981013
H	-4.767416	1.038007	2.676812
H	-4.594576	5.831255	-1.691371
H	-5.206070	6.099813	-0.151250
H	-2.474190	1.718971	-2.718754
H	-2.694841	0.336975	-1.775045
H	-5.819534	0.868130	-0.709551
H	-6.821889	0.251619	-1.921744
H	-1.450886	-2.954872	-0.657427
H	-1.693602	-1.565258	0.212489
H	-4.815584	-4.996814	-3.253336
H	-6.189825	-4.122159	-3.653883

84. FLP_6 - Linker. Acid R = OCH_3. Base R = OCH_3

E_{elec} = -4464.962061 a.u.; **H =** -4463.583592 a.u

C	3.510337	-1.708208	1.395825
C	2.487597	-0.741093	1.566154
C	1.915964	-0.679324	2.852970
C	2.183488	-1.634828	3.835978
C	3.082375	-2.660795	3.569762
C	3.782353	-2.684618	2.358473
P	2.009381	0.365628	0.174208
N	0.359601	0.988125	0.173048
N	-0.711470	0.613514	-0.707556
B	-2.187851	-0.047403	-0.156881
C	-2.886135	-1.008284	-1.370890
C	-4.292698	-1.120551	-1.578573

C	-4.906680	-2.049002	-2.433894
C	-4.127826	-2.909702	-3.191491
C	-2.746125	-2.799983	-3.120253
C	-2.175875	-1.854198	-2.249262
O	-5.104983	-0.228668	-0.926666
C	-6.512627	-0.392417	-0.949063
O	-4.803018	-3.817871	-3.985656
C	-4.008024	-4.672063	-4.801763
O	-0.793371	-1.768981	-2.270307
C	-0.180960	-1.981858	-3.544536
C	3.245745	1.725643	0.109441
C	3.073065	2.864205	-0.722008
C	4.120103	3.735080	-1.047913
C	5.370378	3.534001	-0.469190
C	5.548944	2.527469	0.477339
C	4.492759	1.673215	0.799367
C	2.194729	-0.766137	-1.226618
C	1.785537	-2.115617	-1.044236
C	2.229556	-3.140062	-1.879236
C	3.051393	-2.831115	-2.959723
C	3.437645	-1.514349	-3.212464
C	3.016503	-0.496070	-2.344413
C	0.305854	2.223422	0.848899
O	1.168038	2.543896	1.654588
O	-0.717532	2.986374	0.480146
C	-0.864644	4.223559	1.228920
C	-0.031971	5.339803	0.618663
C	-0.448710	0.930419	-2.023665
O	0.638865	1.242872	-2.506515
O	-1.556845	0.842307	-2.793315
C	-1.399545	1.187989	-4.179441
C	-2.728310	0.864610	-4.841592
C	-3.279864	1.163962	0.305097
C	-4.259395	0.914539	1.307658
C	-5.162157	1.860652	1.800905
C	-5.164831	3.141348	1.256595
C	-4.313974	3.435995	0.201134
C	-3.427209	2.438594	-0.266619
O	-4.333463	-0.379622	1.771348
C	-5.288601	-0.718055	2.763298
O	-6.060038	4.037531	1.813711
C	-6.106539	5.339962	1.241231
O	-2.665888	2.718027	-1.381204
C	-2.819159	3.962273	-2.031630
C	-1.804471	-0.931452	1.214958
C	-1.655600	-0.296960	2.463381
C	-1.639099	-0.947381	3.708438
C	-1.768115	-2.328499	3.745547
C	-1.911256	-3.032910	2.554860
C	-1.917482	-2.338040	1.338258
O	-1.583080	1.082687	2.462147
C	-2.198497	1.782257	3.543275
O	-1.752286	-3.084055	4.902228
C	-1.605460	-2.377870	6.129825
O	-2.050389	-3.064020	0.179329

C	-2.712225	-4.322425	0.225843
O	0.980305	-2.355781	0.008559
O	3.414284	-3.892089	-3.745333
O	3.422124	0.781693	-2.527486
O	4.626504	0.787439	1.818448
O	6.468063	4.308265	-0.726346
O	1.826841	3.096448	-1.166760
O	1.107600	0.368433	3.107913
O	3.251568	-3.585897	4.563336
O	4.297083	-1.590565	0.284275
H	6.502955	2.458010	0.983232
H	3.929973	4.571373	-1.705794
H	1.671377	-1.604113	4.789543
H	4.557681	-3.415977	2.178847
H	1.899141	-4.161582	-1.746818
H	4.052710	-1.271763	-4.066805
H	-2.007195	-4.111834	2.595822
H	-1.538186	-0.367222	4.615903
H	-5.872376	1.628114	2.584815
H	-4.343928	4.406034	-0.275499
H	-5.982907	-2.098213	-2.539207
H	-2.100141	-3.447584	-3.698435
H	-0.566345	0.625224	-4.613113
H	-1.149415	2.254339	-4.259165
H	-2.686678	1.105438	-5.910163
H	-2.964251	-0.197443	-4.721616
H	-3.534218	1.441223	-4.378063
H	-0.574055	4.040431	2.266471
H	-1.937001	4.426217	1.173069
H	-0.205985	6.270449	1.171642
H	1.031000	5.091767	0.671466
H	-5.134094	-1.781722	2.954795
H	-5.127504	-0.156861	3.695424
H	-6.316948	-0.546579	2.413043
H	-2.143636	3.928620	-2.890020
H	-3.847961	4.117415	-2.389503
H	-2.534285	4.809647	-1.388328
H	-6.860017	5.888275	1.810619
H	-5.136960	5.851952	1.327794
H	-6.400611	5.303823	0.182524
H	0.735790	-1.390015	-3.543155
H	0.069960	-3.039523	-3.705423
H	-0.838643	-1.643205	-4.352858
H	-4.713779	-5.294115	-5.356277
H	-3.392980	-4.094912	-5.506988
H	-3.352758	-5.313600	-4.195027
H	-6.894106	0.383715	-0.280976
H	-6.928396	-0.244164	-1.956561
H	-6.811763	-1.382859	-0.576438
C	5.921990	0.517501	2.354052
H	5.769748	-0.314495	3.043525
H	6.621305	0.223493	1.561380
H	6.317455	1.383786	2.899169
C	6.315044	5.385690	-1.656413
H	7.298125	5.853259	-1.724111

H	6.012175	5.015289	-2.644426
H	5.581291	6.117807	-1.295797
C	1.610766	3.960830	-2.283276
H	0.555122	3.846721	-2.520024
H	1.832395	5.004019	-2.022934
H	2.213798	3.638673	-3.138657
C	0.841666	-3.712971	0.452875
H	0.323845	-3.645788	1.408116
H	0.239654	-4.285966	-0.258478
H	1.831102	-4.167638	0.586492
C	4.214253	-3.623760	-4.900998
H	4.370399	-4.592421	-5.377509
H	3.693877	-2.950177	-5.593766
H	5.183181	-3.189646	-4.621147
C	4.294698	1.094807	-3.609901
H	4.508115	2.158610	-3.495835
H	5.231645	0.525855	-3.547245
H	3.806043	0.911958	-4.575398
C	5.216147	-2.636360	-0.033728
H	5.641294	-2.352986	-0.997007
H	6.012865	-2.709362	0.718883
H	4.697270	-3.597298	-0.132427
C	1.141584	0.924298	4.423792
H	0.772516	1.943095	4.307888
H	0.497269	0.364146	5.112206
H	2.169186	0.937586	4.805737
C	4.124226	-4.688976	4.303690
H	4.061093	-5.324311	5.188038
H	3.797674	-5.250938	3.419268
H	5.160829	-4.352915	4.166359
H	-0.310606	5.503071	-0.426871
H	-2.950243	-4.557216	-0.814053
H	-2.068396	-5.112111	0.644154
H	-3.635898	-4.261409	0.814890
H	-2.461212	2.767170	3.154267
H	-3.113079	1.276283	3.868449
H	-1.517164	1.891633	4.398566
H	-1.618936	-3.137788	6.913611
H	-0.651270	-1.832006	6.166668
H	-2.431159	-1.670127	6.288300
H	-5.134094	-1.781722	2.954795
H	-5.127504	-0.156861	3.695424
H	-6.316948	-0.546579	2.413043
H	-2.143636	3.928620	-2.890020
H	-3.847961	4.117415	-2.389503
H	-2.534285	4.809647	-1.388328
H	-6.860017	5.888275	1.810619
H	-5.136960	5.851952	1.327794
H	-6.400611	5.303823	0.182524
H	0.735790	-1.390015	-3.543155
H	0.069960	-3.039523	-3.705423
H	-0.838643	-1.643205	-4.352858
H	-4.713779	-5.294115	-5.356277
H	-3.392980	-4.094912	-5.506988
H	-3.352758	-5.313600	-4.195027

H	-6.894106	0.383715	-0.280976
H	-6.928396	-0.244164	-1.956561
H	-6.811763	-1.382859	-0.576438
C	5.921990	0.517501	2.354052
H	5.769748	-0.314495	3.043525
H	6.621305	0.223493	1.561380
H	6.317455	1.383786	2.899169
C	6.315044	5.385690	-1.656413
H	7.298125	5.853259	-1.724111
H	6.012175	5.015289	-2.644426
H	5.581291	6.117807	-1.295797
C	1.610766	3.960830	-2.283276
H	0.555122	3.846721	-2.520024
H	1.832395	5.004019	-2.022934
H	2.213798	3.638673	-3.138657
C	0.841666	-3.712971	0.452875
H	0.323845	-3.645788	1.408116
H	0.239654	-4.285966	-0.258478
H	1.831102	-4.167638	0.586492
C	4.214253	-3.623760	-4.900998
H	4.370399	-4.592421	-5.377509
H	3.693877	-2.950177	-5.593766
H	5.183181	-3.189646	-4.621147
C	4.294698	1.094807	-3.609901
H	4.508115	2.158610	-3.495835
H	5.231645	0.525855	-3.547245
H	3.806043	0.911958	-4.575398
C	5.216147	-2.636360	-0.033728
H	5.641294	-2.352986	-0.997007
H	6.012865	-2.709362	0.718883
H	4.697270	-3.597298	-0.132427
C	1.141584	0.924298	4.423792
H	0.772516	1.943095	4.307888
H	0.497269	0.364146	5.112206
H	2.169186	0.937586	4.805737
C	4.124226	-4.688976	4.303690
H	4.061093	-5.324311	5.188038
H	3.797674	-5.250938	3.419268
H	5.160829	-4.352915	4.166359

85. FLP$_7$ - Linker. Acid R = H. Base R = CN

E_{elec} = -3232.965544 a.u.; **H =** -3232.204004 a.u

C	-4.497070	1.566016	3.739477
C	-5.342186	0.974934	2.799372
C	-4.846126	0.546461	1.565286
C	-3.494959	0.697030	1.210409
C	-2.676249	1.303366	2.178337
C	-3.147685	1.728311	3.419949
B	-2.922007	0.216412	-0.249602
C	-3.863883	-0.940264	-0.906949
C	-5.066978	-0.550965	-1.531291
C	-5.955322	-1.473203	-2.087984
C	-5.666197	-2.837708	-2.045588
C	-4.483531	-3.256056	-1.434418
C	-3.610407	-2.321191	-0.873683

N	-1.387788	-0.440038	0.086799
C	-1.108887	-1.279015	1.117776
O	-2.161782	-1.651430	1.819542
C	-1.918984	-2.313277	3.103163
C	-3.267773	-2.402112	3.785572
N	-0.245328	-0.301099	-0.746865
C	-0.153017	-0.966541	-1.992465
O	-1.289702	-1.359894	-2.468143
C	-1.312957	-1.950961	-3.828080
C	-0.960684	-3.422692	-3.780753
P	1.355470	-0.113490	-0.151129
C	1.337074	0.506750	1.594241
C	0.781969	1.775442	1.881670
C	0.635184	2.224903	3.209289
C	1.087859	1.450210	4.270349
C	1.736726	0.235306	4.001012
C	1.856524	-0.230575	2.693818
C	0.409456	2.747866	0.903236
C	2.584306	-1.454252	2.566889
C	0.921908	1.897409	5.616982
C	2.446764	-1.594772	-0.393649
C	3.819008	-1.462245	-0.738236
C	4.701848	-2.550328	-0.645992
C	4.235097	-3.818000	-0.306048
C	2.856652	-4.009010	-0.188327
C	1.971306	-2.927848	-0.250900
C	4.424954	-0.321021	-1.351228
C	0.602337	-3.340379	-0.296866
C	5.137998	-4.915518	-0.167355
C	2.111524	1.384147	-0.954007
C	1.626237	2.023737	-2.121961
C	2.090670	3.300364	-2.487746
C	3.060042	3.962953	-1.738504
C	3.606898	3.327056	-0.620975
C	3.140267	2.068597	-0.240622
C	0.673230	1.523173	-3.058190
C	3.839717	1.488863	0.865484
C	3.501373	5.268504	-2.115050
O	0.980156	-1.063995	-2.478235
O	0.063860	-1.636647	1.398729
C	-2.741660	1.489653	-1.260328
C	-2.572332	2.803171	-0.789870
C	-2.495722	3.904531	-1.647720
C	-2.616371	3.728157	-3.024959
C	-2.810657	2.440755	-3.526466
C	-2.866635	1.350855	-2.655741
H	5.750146	-2.391298	-0.871744
H	2.451676	-5.004779	-0.049251
H	1.667899	3.769694	-3.368529
H	4.397814	3.797528	-0.048570
H	0.167126	3.186506	3.385563
H	2.150917	-0.354146	4.810832
H	-2.527683	2.981826	0.280454
H	-3.038408	0.364011	-3.072706
H	-2.354315	4.899684	-1.233565

H	-2.911258	2.281273	-4.596990
H	-2.574160	4.581805	-3.697265
H	-5.309280	0.508080	-1.595206
H	-2.698092	-2.695378	-0.422188
H	-6.870713	-1.123640	-2.560636
H	-4.236585	-4.315130	-1.390152
H	-6.350151	-3.561739	-2.481767
H	-5.527863	0.065789	0.869369
H	-1.620103	1.420664	1.957445
H	-6.395778	0.835920	3.031710
H	-2.469541	2.183759	4.140302
H	-4.880361	1.891473	4.703043
H	-1.204779	-1.705717	3.668013
H	-1.473596	-3.291600	2.899763
H	-3.150355	-2.886432	4.760802
H	-3.687549	-1.402797	3.929662
H	-3.964554	-2.990506	3.182317
H	-0.637476	-1.358537	-4.447948
H	-2.347859	-1.782462	-4.126333
H	-1.092933	-3.842560	-4.784118
H	0.079199	-3.570375	-3.478542
H	-1.615821	-3.953004	-3.086242
N	5.871656	-5.809234	-0.056153
N	5.016217	0.482712	-1.947815
N	-0.415487	-3.875121	-0.472708
N	3.227429	-2.421072	2.585971
N	0.782207	2.255618	6.713180
N	0.175880	3.664556	0.227886
N	4.489246	1.063763	1.731394
N	3.863748	6.330105	-2.416449
N	-0.022320	1.263319	-3.952362

86. FLP_8 - Linker. Acid R = H. Base R = CF_3

E_{elec} = -4063.563250 a.u.; **H =** -4062.800833 a.u

C	4.102113	-0.476297	-3.213910
C	3.641795	-1.026451	-2.002227
C	3.362626	-2.405520	-2.030873
C	3.472724	-3.172661	-3.192089
C	3.890830	-2.583291	-4.384249
C	4.217952	-1.226051	-4.385130
B	3.594288	-0.145555	-0.619540
N	2.038238	0.541697	-0.344614
N	0.831021	0.284740	-1.057697
P	-0.695616	0.191875	-0.266427
C	-1.706468	-1.414046	-0.726484
C	-1.508551	-2.296108	-1.848646
C	-2.246975	-3.476642	-1.955961
C	-3.135848	-3.897067	-0.981855
C	-3.344310	-3.086079	0.106310
C	-2.682895	-1.856972	0.217131
C	-0.605699	-2.173161	-3.106186
F	-0.330352	-3.415122	-3.605909
C	-3.832552	-5.229679	-1.124983
F	-4.308341	-5.405489	-2.383084
C	-3.223918	-1.136343	1.449711

F	-4.565081	-1.296379	1.562219
H	3.071350	-2.904992	-1.113952
H	4.392209	0.570388	-3.236766
C	3.984171	-1.054976	0.683206
C	5.013509	-2.012556	0.551051
C	5.491976	-2.760983	1.626900
C	4.960317	-2.575942	2.903612
C	3.948436	-1.633347	3.075222
C	3.477229	-0.897562	1.984444
C	4.604399	1.141256	-0.745851
C	5.907121	1.094637	-0.220988
C	6.802697	2.158544	-0.355760
C	6.422382	3.322623	-1.023692
C	5.134198	3.400473	-1.556507
C	4.253827	2.327411	-1.417755
C	1.799522	1.511042	0.578374
O	0.640389	1.918269	0.844243
O	2.878679	1.957464	1.195961
C	2.713790	2.928513	2.277387
C	4.107774	3.437598	2.582189
C	0.609843	0.746057	-2.361849
O	-0.564349	0.770567	-2.743468
O	1.681087	1.070589	-3.027174
C	1.530135	1.422333	-4.453441
C	2.325903	2.685781	-4.705773
C	-0.287072	-0.375341	1.539012
C	0.308212	-1.654324	1.757517
C	0.636733	-2.104295	3.045418
C	0.429289	-1.319132	4.154657
C	-0.133946	-0.068001	3.977286
C	-0.507369	0.402529	2.718983
C	0.652041	-2.752493	0.752376
F	1.753407	-3.441609	1.115759
C	0.801417	-1.779760	5.542867
F	-0.283859	-1.796077	6.360899
C	-1.196286	1.756184	2.935367
F	-0.331054	2.643063	3.493141
C	-1.825795	1.723645	-0.718017
C	-3.227991	1.566593	-0.970351
C	-4.133132	2.572547	-0.623537
C	-3.699219	3.809468	-0.182834
C	-2.352869	4.087911	-0.321604
C	-1.415220	3.090763	-0.619328
C	-3.939595	0.493042	-1.800740
F	-4.860424	1.104951	-2.600803
C	-4.666263	4.844058	0.332454
F	-4.248653	6.105919	0.065713
C	-0.094098	3.800818	-0.950117
F	0.390711	4.518339	0.090138
F	-0.360838	-3.675077	0.701046
F	0.871192	-2.317416	-0.494538
F	-1.755133	2.354307	1.881969
F	-2.204942	1.590217	3.847170
F	1.336354	-3.020300	5.547666
F	1.707845	-0.938281	6.109070

F	-3.137241	-0.183207	-2.632415
F	-4.650837	-0.403195	-1.060266
F	0.908668	3.073887	-1.462499
F	-0.386927	4.720220	-1.930259
F	-5.901624	4.696938	-0.207708
F	-4.806828	4.746202	1.681888
F	-1.260187	-1.514745	-4.092810
F	0.596525	-1.614362	-2.939935
F	-2.695015	-1.634097	2.598576
F	-3.019429	0.201176	1.437648
F	-2.978311	-6.255504	-0.873985
F	-4.874974	-5.352251	-0.269506
H	-5.193009	2.387980	-0.753169
H	-1.999506	5.104580	-0.194105
H	-2.105629	-4.103120	-2.825429
H	-4.050960	-3.377849	0.872900
H	1.092057	-3.078939	3.155703
H	-0.314983	0.562282	4.840605
H	3.237906	-4.234510	-3.161930
H	4.584893	-0.754742	-5.295240
H	3.984135	-3.175648	-5.291343
H	6.232186	0.210569	0.321059
H	3.255237	2.423499	-1.828949
H	7.801001	2.077947	0.069430
H	4.812836	4.300166	-2.078621
H	7.115335	4.154217	-1.125901
H	5.456225	-2.174269	-0.428559
H	2.689709	-0.175549	2.175409
H	6.285046	-3.488050	1.466818
H	3.531879	-1.461082	4.065293
H	5.330683	-3.150673	3.748703
H	2.260574	2.409475	3.127580
H	2.034324	3.714001	1.939529
H	4.057643	4.175778	3.389664
H	4.756863	2.614672	2.893427
H	4.546572	3.904744	1.696241
H	0.465404	1.535486	-4.664862
H	1.932428	0.558935	-4.988660
H	2.273390	2.929957	-5.772249
H	1.918133	3.525307	-4.135721
H	3.374409	2.547461	-4.430188

87. FLP_{11} - Linker. Acid R = H. Base R = NH_2

E_{elec} = -2900.958576 a.u.; **H =** -2900.037332 a.u

C	-3.462779	-3.740049	2.078771
C	-2.708687	-2.977056	2.969130
C	-2.325906	-1.673181	2.633636
C	-2.647674	-1.080807	1.398279
C	-3.461491	-1.861536	0.547288
C	-3.854320	-3.161742	0.866807
B	-2.167559	0.432611	0.938803
C	-3.470883	1.259973	0.383733
C	-4.721304	1.037440	0.992307
C	-5.870217	1.746232	0.632961
C	-5.812998	2.716666	-0.367468

C	-4.587549	2.969187	-0.986576
C	-3.446728	2.258931	-0.607608
N	-1.017171	0.315189	-0.299782
C	-1.358513	0.348920	-1.630440
O	-2.542327	-0.246618	-1.845459
C	-3.116002	-0.058060	-3.167441
C	-4.514092	-0.641919	-3.110622
N	0.339277	0.711004	-0.092373
C	0.580267	2.065052	0.168945
O	-0.471656	2.823552	-0.144073
C	-0.406525	4.214055	0.293989
C	0.362034	5.065553	-0.703316
P	1.639708	-0.449324	-0.497475
C	0.813072	-1.618624	-1.575705
C	-0.222083	-2.487488	-1.053751
C	-1.059342	-3.190853	-1.922395
C	-0.908306	-3.088221	-3.306534
C	0.138156	-2.326225	-3.835299
C	0.996627	-1.601153	-3.005535
N	-0.375561	-2.726876	0.286543
N	2.044749	-0.943847	-3.610003
N	-1.821674	-3.701740	-4.152797
C	2.990545	0.499368	-1.188743
C	4.349096	0.112598	-0.868644
C	5.372090	1.063222	-0.877616
C	5.131835	2.371872	-1.311764
C	3.880901	2.708058	-1.833507
C	2.823882	1.791693	-1.824451
N	4.701281	-1.189598	-0.631512
N	1.666948	2.132408	-2.449636
N	6.172147	3.292223	-1.346625
C	2.226131	-1.337260	0.953063
C	2.577555	-0.654381	2.172799
C	2.751346	-1.381922	3.362503
C	2.742960	-2.774372	3.363052
C	2.650602	-3.455620	2.137883
C	2.444495	-2.763122	0.951166
N	2.813459	0.684124	2.210978
N	2.623215	-3.469643	-0.240965
N	2.824616	-3.486070	4.548230
O	1.626295	2.510088	0.627978
O	-0.698462	0.830794	-2.560555
C	-1.496547	1.241891	2.192995
C	-0.384777	0.754228	2.905352
C	0.117285	1.388375	4.043028
C	-0.468114	2.565957	4.506522
C	-1.562452	3.088763	3.815711
C	-2.063865	2.431586	2.690494
H	6.385005	0.750668	-0.634244
H	3.721124	3.687822	-2.278158
H	2.945579	-0.835896	4.283000
H	2.768508	-4.536432	2.105794
H	-1.862741	-3.786534	-1.497219
H	0.293541	-2.284886	-4.910982
H	-0.034359	-2.037996	0.945266

H	-1.282009	-3.092981	0.560053
H	-2.353301	-4.467333	-3.758251
H	1.891623	-0.653101	-4.565918
H	2.583689	-0.291181	-3.050787
H	2.059124	-3.144268	-1.026346
H	2.558967	-4.475348	-0.126365
H	3.140957	-4.444842	4.484443
H	3.187053	-2.991921	5.353254
H	2.489679	1.313289	1.465636
H	2.765652	1.115547	3.125137
H	0.802439	1.564497	-2.433819
H	1.547273	3.101990	-2.704936
H	6.931660	3.123562	-0.698977
H	5.897991	4.265978	-1.384653
H	4.033607	-1.953001	-0.609071
H	5.561568	-1.361096	-0.131226
H	0.101529	-0.158961	2.571993
H	-2.927777	2.852463	2.182888
H	0.953117	0.946141	4.583075
H	-2.035611	4.005951	4.160850
H	-0.083717	3.063429	5.393901
H	-4.799686	0.283765	1.772485
H	-2.512929	2.484585	-1.113234
H	-6.813097	1.535140	1.133600
H	-4.518556	3.722408	-1.769808
H	-6.704896	3.266499	-0.658690
H	-1.756788	-1.108260	3.365446
H	-3.782490	-1.437522	-0.395156
H	-2.417839	-3.392370	3.931778
H	-4.473813	-3.725043	0.170741
H	-3.759431	-4.755407	2.330821
H	-2.476076	-0.564072	-3.896249
H	-3.128482	1.013779	-3.388507
H	-5.006116	-0.514089	-4.080990
H	-4.475865	-1.711651	-2.881544
H	-5.107354	-0.133577	-2.344548
H	0.041589	4.237877	1.289757
H	-1.457637	4.501139	0.359267
H	0.320663	6.116112	-0.393942
H	1.409573	4.754374	-0.741470
H	-0.082450	4.984944	-1.701000
H	-1.512454	-3.876294	-5.100522

88. FLP_{12} - Linker. Acid R = CN. Base R = CN

E_{elec} = -4063.563250 a.u.; **H =** -4062.800833 a.u

C	2.431017	-0.300796	2.757955
C	2.284878	0.573119	1.636206
C	2.156990	1.935656	2.029582
C	2.184274	2.377616	3.365388
C	2.304920	1.474504	4.415095
C	2.420903	0.126289	4.097117
B	2.328442	-0.021973	0.054022
N	0.758840	-0.284955	-0.427500
N	-0.386856	-0.619235	0.396815
P	-1.983420	-0.174383	-0.269619

C	-2.977326	0.158447	1.342105
C	-2.571555	-0.020983	2.686511
C	-3.380760	0.370416	3.763449
C	-4.625483	0.964282	3.554060
C	-5.066231	1.152444	2.244959
C	-4.260312	0.754435	1.170666
C	-1.346723	-0.604628	3.135328
C	-5.438685	1.364400	4.658476
C	-4.892199	0.912024	-0.104908
C	1.929906	3.068847	1.184613
N	1.693516	4.099751	0.699586
C	2.294017	1.916064	5.773781
N	2.276111	2.274285	6.878764
C	2.676704	-1.715277	2.726735
N	2.944911	-2.826812	2.941148
C	3.106006	1.010300	-1.053337
C	4.386729	0.724125	-1.625062
C	5.235645	1.710799	-2.154513
C	4.816998	3.038324	-2.251271
C	3.476029	3.305574	-1.981106
C	2.653931	2.298129	-1.453395
C	5.694137	4.059424	-2.727693
N	6.406254	4.890433	-3.118020
C	4.875157	-0.591188	-1.929342
N	5.330969	-1.554655	-2.397860
C	1.267868	2.605948	-1.578798
N	0.195511	2.874629	-1.934685
C	3.236943	-1.392246	0.066318
C	4.519920	-1.346353	0.687834
C	5.374299	-2.450191	0.769645
C	4.993158	-3.664237	0.198602
C	3.775588	-3.741165	-0.482962
C	2.928462	-2.628051	-0.550059
C	5.036635	-0.130771	1.248371
N	5.521378	0.833693	1.681468
C	5.842989	-4.809673	0.285868
N	6.527345	-5.745585	0.359668
C	1.738767	-2.839626	-1.316884
N	0.790550	-3.100107	-1.937306
C	0.330358	-0.203871	-1.698440
O	-0.934037	-0.220800	-1.921997
O	1.218706	-0.093242	-2.637612
C	0.816187	0.316440	-4.017913
C	2.098471	0.673659	-4.732906
C	-0.535875	-1.956687	0.878670
O	-1.682862	-2.364316	1.034399
O	0.558571	-2.591495	1.172875
C	0.350323	-3.943750	1.796307
C	-0.023429	-4.993153	0.771375
C	-2.180207	1.627238	-0.750682
C	-1.822312	2.648303	0.165595
C	-1.970312	4.007958	-0.145994
C	-2.526178	4.394901	-1.364173
C	-2.896211	3.411194	-2.281589
C	-2.706101	2.053754	-1.994278

C	-1.269218	2.423530	1.462204
C	-2.701316	5.778111	-1.675798
C	-3.132221	1.185329	-3.043510
C	-3.126681	-1.471299	-1.056164
C	-4.195310	-2.048179	-0.292030
C	-5.235290	-2.771423	-0.889588
C	-5.240509	-3.024080	-2.260669
C	-4.124121	-2.644124	-2.993569
C	-3.066257	-1.917867	-2.409041
C	-4.347814	-2.088804	1.133714
C	-6.327249	-3.717983	-2.874732
C	-2.001000	-1.828114	-3.361858
H	-6.028958	-3.153110	-0.257733
H	-4.041749	-2.903812	-4.042258
H	-3.019421	0.208341	4.772396
H	-6.039443	1.587084	2.046359
H	-1.637241	4.749902	0.570519
H	-3.331258	3.687748	-3.235121
H	2.082750	3.437325	3.568334
H	2.512321	-0.614608	4.882479
H	6.330548	-2.356261	1.271468
H	3.488445	-4.664683	-0.973753
H	6.215116	1.419076	-2.517047
H	3.050855	4.281256	-2.187349
H	0.131818	1.160247	-3.908289
H	0.303916	-0.538860	-4.455676
H	1.857481	0.925085	-5.770711
H	2.589358	1.535815	-4.273596
H	2.789890	-0.173556	-4.734996
H	-0.413439	-3.805445	2.562719
H	1.319122	-4.122783	2.258032
H	-0.154829	-5.940880	1.305734
H	-0.959019	-4.751344	0.263266
H	0.762278	-5.126327	0.025668
N	-7.214456	-4.281564	-3.369082
N	-4.640784	-2.300604	2.239236
N	-1.297996	-1.983917	-4.273257
N	-3.520499	0.595263	-3.965275
N	-2.848548	6.901721	-1.930615
N	-0.845072	2.377016	2.542670
N	-5.512357	1.030501	-1.081685
N	-6.102834	1.695164	5.552106
N	-0.431755	-1.077616	3.671941

89. FLP_{14} - Linker. Acid R = CN. Base R = H

E_{elec} = -3233.056996 a.u.; **H =** -3232.293376 a.u

C	2.198749	-1.238818	2.362298
C	1.368647	-0.302625	1.693031
C	0.328358	0.224545	2.511018
C	0.043497	-0.233661	3.804741
C	0.829628	-1.235608	4.375169
C	1.934356	-1.703410	3.659716
B	1.492632	0.217951	0.107252
N	0.144329	-0.340184	-0.657341
N	-1.024689	-0.724664	0.077268

P	-2.648255	-0.212950	-0.337142
C	-3.581993	0.485029	1.070298
C	-3.430798	0.067446	2.401066
C	-4.219698	0.618850	3.405838
C	-5.184743	1.579629	3.104388
C	-5.355526	1.989721	1.784038
C	-4.558909	1.454160	0.773976
H	-2.702783	-0.689693	2.651817
H	-5.797917	2.005980	3.893092
H	-4.699351	1.803824	-0.242515
C	1.507878	1.883062	0.198359
C	2.067645	2.560890	1.322592
C	1.954940	3.938885	1.532494
C	1.291398	4.734175	0.596778
C	0.832250	4.138034	-0.577391
C	0.957484	2.753816	-0.772491
C	2.867325	-0.358461	-0.645109
C	4.026508	0.445883	-0.835156
C	5.266287	-0.062129	-1.267821
C	5.406627	-1.405165	-1.601995
C	4.271672	-2.214355	-1.549183
C	3.050783	-1.702477	-1.092199
C	0.029909	-0.752444	-1.985743
O	-0.975968	-1.287774	-2.436802
O	1.124080	-0.489557	-2.704870
C	1.184741	-1.135106	-4.025863
C	0.466810	-0.345215	-5.104016
C	-1.093094	-1.933595	0.796981
O	-2.158162	-2.239417	1.321619
O	0.029406	-2.614294	0.850050
C	-0.015650	-3.880324	1.612459
C	-0.728905	-4.975335	0.841741
C	-2.582326	1.128096	-1.534390
C	-2.388999	2.450859	-1.098743
C	-2.536255	3.500016	-2.001432
C	-2.859625	3.241217	-3.333237
C	-3.034256	1.927255	-3.769649
C	-2.905099	0.869062	-2.875517
H	-2.140652	2.656283	-0.062130
H	-2.967266	4.063397	-4.034821
H	-3.047210	-0.152264	-3.208662
C	-3.659074	-1.567331	-1.014334
C	-5.041567	-1.337775	-1.082253
C	-5.890209	-2.317770	-1.594156
C	-5.370403	-3.538552	-2.021757
C	-3.996768	-3.772961	-1.941688
C	-3.139548	-2.794794	-1.446104
H	-5.464940	-0.401823	-0.733845
H	-6.032623	-4.305617	-2.413192
H	-2.074615	-2.980141	-1.420128
H	-6.957622	-2.124907	-1.647838
H	-3.582982	-4.721909	-2.270157
H	-4.077840	0.290365	4.431546
H	-6.102320	2.736879	1.532037
H	-2.392756	4.521054	-1.660074

H	-3.272348	1.723882	-4.809197
C	-0.480042	1.336453	2.106137
C	3.432623	-1.753239	1.844561
H	-0.776515	0.205712	4.362202
H	2.606287	-2.425020	4.111336
C	0.525429	-1.746304	5.674225
C	4.087856	1.875533	-0.707707
C	2.019137	-2.689591	-1.210505
H	6.109995	0.613470	-1.354774
H	4.329368	-3.253471	-1.852817
C	6.662088	-1.931693	-2.034661
C	2.883611	1.897787	2.297615
C	0.568086	2.351565	-2.088551
H	2.394040	4.384977	2.417707
H	0.386456	4.745386	-1.357090
C	1.115918	6.135461	0.812914
H	0.776409	-2.142487	-3.913886
H	2.259747	-1.193727	-4.212512
H	0.583189	-0.879203	-6.054766
H	-0.597550	-0.262248	-4.875258
H	0.882298	0.658453	-5.203858
H	-0.490452	-3.667494	2.573660
H	1.042430	-4.101616	1.756841
H	-0.642777	-5.909636	1.407802
H	-1.789139	-4.740316	0.723133
H	-0.266165	-5.110887	-0.138067
N	0.290629	2.217922	-3.208561
N	0.955611	7.273437	0.986079
N	3.604943	1.463870	3.100411
N	4.306085	3.018035	-0.727011
N	7.679252	-2.362323	-2.395561
N	1.310312	-3.576949	-1.459722
N	-1.156400	2.262624	1.915303
N	4.466670	-2.223982	1.598126
N	0.259102	-2.171574	6.722524

90. FLP$_{15}$ - Linker. Acid R = CF$_3$. Base R = H
E$_{elec}$ = -5437.283123 a.u.; **H =** -5436.453511 a.u

C	-0.068519	-1.974823	1.190909
C	0.217021	-0.565077	1.201605
C	0.188936	-0.032789	2.544319
C	0.051544	-0.825399	3.685523
C	-0.180733	-2.184637	3.602450
C	-0.275035	-2.743554	2.349655
B	0.744674	0.210487	-0.284534
C	1.444478	1.825429	-0.163609
C	2.857161	2.055472	-0.317843
C	3.540182	3.107446	0.302896
C	2.858789	4.132233	0.929187
C	1.486114	4.155712	0.764309
C	0.801093	3.058847	0.231273
C	2.015063	-0.972622	-0.742108
C	2.892396	-1.507884	0.266265
C	3.670147	-2.662676	0.115967
C	3.702845	-3.349415	-1.077993

C	3.016336	-2.795767	-2.139247
C	2.225126	-1.648643	-1.991932
H	0.106727	-0.366618	4.662351
H	-0.473201	-3.803714	2.256292
H	4.266863	-3.010387	0.950784
H	3.093691	-3.257419	-3.116588
H	4.620850	3.145402	0.239882
H	0.929107	5.031566	1.072301
C	-0.394501	-2.991226	4.852894
C	0.222771	1.446546	2.942395
C	-0.134332	-2.968035	0.022914
C	1.749576	-1.296162	-3.399666
C	4.452298	-4.643792	-1.224443
C	3.237626	-0.922420	1.629573
C	3.790270	1.370483	-1.316180
C	3.571101	5.217810	1.682940
C	-0.636653	3.482842	-0.021730
F	3.598692	4.964490	3.022553
F	4.862005	5.361938	1.286287
F	2.964487	6.427324	1.531017
F	-1.142653	4.313713	0.934739
F	-1.545225	2.495918	-0.160258
F	-0.704543	4.221505	-1.180171
F	4.665463	2.299324	-1.826306
F	3.141746	0.896676	-2.398936
F	4.588204	0.387449	-0.822481
F	2.842300	0.338938	1.848341
F	2.751177	-1.691261	2.654806
F	4.593778	-0.908453	1.808924
F	5.485248	-4.742318	-0.348742
F	4.962896	-4.801686	-2.474120
F	3.642371	-5.718770	-0.996746
F	2.838704	-1.129615	-4.218036
F	1.058189	-2.347388	-3.955062
F	1.003098	-0.204370	-3.570576
F	1.407299	2.075006	2.810670
F	-0.116743	1.632770	4.256681
F	-0.709835	2.142328	2.252861
F	-1.636872	-2.756526	5.382759
F	0.500455	-2.675675	5.822116
F	-0.309943	-4.324948	4.633441
F	-1.243997	-3.774232	0.164976
F	-0.213565	-2.495308	-1.224980
F	0.922419	-3.826368	0.061942
N	-0.591307	0.332940	-1.252657
N	-1.820898	-0.404909	-1.160824
C	-0.723817	1.263658	-2.293824
C	-2.286663	-0.988592	-2.366938
O	-1.741229	1.404221	-2.963350
O	0.383980	1.988786	-2.483387
O	-3.501142	-1.183304	-2.456389
O	-1.389215	-1.348904	-3.247081
C	0.365582	2.876662	-3.645591
C	-1.919880	-1.832147	-4.536472
C	1.626260	3.715050	-3.581264

H	-0.542688	3.480958	-3.600759
H	0.327676	2.246732	-4.539806
C	-2.232541	-0.657764	-5.444503
H	-2.799069	-2.446624	-4.330952
H	-1.108070	-2.449813	-4.916692
H	1.633572	4.415204	-4.423450
H	1.659535	4.290641	-2.652060
H	2.519753	3.090099	-3.641498
H	-2.576658	-1.039904	-6.412262
H	-3.013592	-0.028218	-5.012399
H	-1.339821	-0.047689	-5.602587
P	-3.405426	-0.005562	-0.373085
C	-4.289919	-1.520593	0.155558
C	-3.240389	0.763487	1.265179
C	-4.552922	1.039951	-1.328796
C	-3.968774	-2.819394	-0.245521
C	-5.370422	-1.319508	1.034805
C	-2.899510	-0.068014	2.345537
C	-3.741935	2.047648	1.526088
C	-5.803759	0.503602	-1.671805
C	-4.232461	2.346530	-1.722686
C	-4.695897	-3.903300	0.245416
C	-6.116488	-2.402415	1.489923
C	-3.053280	0.378402	3.653387
C	-3.880573	2.493901	2.837936
C	-6.731711	1.277773	-2.362990
C	-5.165679	3.111478	-2.419534
C	-5.773291	-3.699868	1.105521
H	-4.416171	-4.908462	-0.055670
H	-6.956047	-2.230106	2.156978
C	-3.543385	1.660303	3.903106
H	-2.789268	-0.280012	4.474905
H	-4.262225	3.493484	3.022813
C	-6.418293	2.585798	-2.734111
H	-7.695782	0.849737	-2.622310
H	-4.901823	4.120202	-2.723660
H	-7.141639	3.186742	-3.278121
H	-6.044633	-0.522182	-1.417415
H	-3.254510	2.759331	-1.524371
H	-5.630984	-0.317544	1.361451
H	-6.343141	-4.546929	1.476816
H	-3.153050	-3.000105	-0.927757
H	-3.662195	2.006759	4.925627
H	-4.039412	2.697129	0.712782
H	-2.544016	-1.077287	2.166611

91. FLP_{16} - Linker. Acid R = CN. Base R = NH_2

E_{elec} = -3731.556547 a.u.; **H =** -3730.634232 a.u

C	-4.497985	-3.415922	-1.172448
C	-5.033602	-2.240655	-0.656092
C	-4.224334	-1.103485	-0.470140
C	-2.808654	-1.091166	-0.675180
C	-2.377621	-2.246102	-1.401760
C	-3.178513	-3.375615	-1.620181
B	-1.820107	0.104808	-0.010642

C	-2.749704	1.503532	0.217415
C	-3.482685	1.673655	1.437523
C	-4.163089	2.841326	1.798156
C	-4.201731	3.923360	0.922304
C	-3.618348	3.768686	-0.332971
C	-2.926709	2.592613	-0.679183
N	-0.448685	0.324934	-0.893180
C	-0.490515	0.366334	-2.277427
O	-1.735199	0.175846	-2.734193
C	-1.877479	0.101212	-4.191830
C	-3.332022	-0.227281	-4.457716
N	0.792614	0.836493	-0.354908
C	0.949619	2.239503	-0.360447
O	0.001344	2.881812	-1.035375
C	0.071870	4.350595	-0.970208
C	1.041453	4.897693	-2.000046
P	2.390707	-0.150897	-0.306686
C	2.165447	-1.588762	-1.335186
C	1.453412	-2.728012	-0.808130
C	1.623650	-4.000616	-1.341484
C	2.435100	-4.202571	-2.464148
C	3.070571	-3.107888	-3.050185
C	2.926194	-1.809001	-2.544478
N	0.546965	-2.587727	0.217540
N	3.600090	-0.812743	-3.196210
N	2.620624	-5.478730	-2.965800
C	3.751372	0.932008	-0.758991
C	4.972914	0.939156	0.019801
C	5.850169	2.027419	-0.050052
C	5.639874	3.088938	-0.930971
C	4.559501	3.023510	-1.814138
C	3.645682	1.973060	-1.752285
N	5.346345	-0.084458	0.857215
N	2.676330	1.934747	-2.722655
N	6.549639	4.129986	-1.009178
C	2.656201	-0.767806	1.369581
C	2.494012	0.055961	2.540442
C	2.596155	-0.496389	3.829037
C	2.938823	-1.832761	4.022792
C	3.302299	-2.596689	2.899265
C	3.254725	-2.061572	1.617533
N	2.227177	1.381035	2.455801
N	3.992903	-2.745294	0.645617
N	3.031657	-2.367474	5.289220
O	1.880840	2.802679	0.202543
O	0.466973	0.533897	-3.025515
C	-1.334841	-0.323607	1.536639
C	-1.494716	-1.591268	2.159297
C	-1.133927	-1.852648	3.491206
C	-0.606814	-0.841958	4.291712
C	-0.467200	0.431453	3.747572
C	-0.812147	0.671988	2.412143
H	6.757420	1.997900	0.549787
H	4.428226	3.788749	-2.575683
H	2.437572	0.150382	4.688213

H	3.739634	-3.582886	3.039925
H	1.062379	-4.828603	-0.915472
H	3.652160	-3.246110	-3.958956
H	0.698175	-1.823356	0.867258
H	0.277418	-3.446481	0.681606
H	1.899686	-6.154877	-2.746711
H	2.945347	-5.542519	-3.922390
H	4.020937	-1.086548	-4.074224
H	3.240418	0.140840	-3.206605
H	4.239984	-3.691969	0.910932
H	3.669276	-2.697287	-0.315174
H	3.051490	-3.376279	5.358392
H	2.488382	-1.923074	6.021011
H	2.271671	1.921238	1.585062
H	2.187173	1.914349	3.311222
H	1.803231	1.386096	-2.647292
H	2.574807	2.769132	-3.284495
H	7.121648	4.292717	-0.190509
H	6.223075	4.982489	-1.445493
H	5.080920	-1.045047	0.648817
H	6.276838	0.000150	1.243488
C	-2.096763	-2.751500	1.567772
C	-0.692798	2.059574	2.069431
H	-1.291032	-2.845203	3.897629
H	-0.096743	1.247497	4.356643
C	-0.181836	-1.102620	5.628625
C	-3.708124	0.619322	2.386587
C	-2.492418	2.683353	-2.044809
H	-4.677800	2.886390	2.751118
H	-3.698585	4.564095	-1.065826
C	-4.854950	5.142373	1.279366
C	-5.051569	0.047212	-0.236551
C	-1.172145	-2.354838	-2.161279
H	-6.089808	-2.176130	-0.419685
H	-2.757999	-4.221877	-2.151190
C	-5.291896	-4.591908	-1.329400
H	-1.192480	-0.672328	-4.547857
H	-1.587418	1.071897	-4.599290
H	-3.498072	-0.280768	-5.538608
H	-3.603957	-1.191616	-4.018090
H	-3.984470	0.547420	-4.045276
H	0.338950	4.625364	0.050632
H	-0.952489	4.656806	-1.180495
H	0.988765	5.992199	-1.989490
H	2.064999	4.598101	-1.759174
H	0.770770	4.551287	-3.002311
N	-2.525152	-3.800968	1.305675
N	0.220545	-1.312488	6.699107
N	-0.603327	3.214531	1.965629
N	-5.375663	6.139775	1.570394
N	-4.033700	-0.160839	3.186256
N	-2.265695	2.990496	-3.143341
N	-5.932083	-5.552545	-1.464221
N	-5.879794	0.863853	-0.192913
N	-0.371599	-2.565982	-2.979549
